# A Profile of the In Vitro Anti-Tumor Activity and In Silico ADME Predictions of Novel Benzothiazole Amide-Functionalized Imidazolium Ionic Liquids

**DOI:** 10.3390/ijms20122865

**Published:** 2019-06-12

**Authors:** Fawzia Al-blewi, Nadjet Rezki, Arshi Naqvi, Husna Qutb Uddin, Salsabeel Al-Sodies, Mouslim Messali, Mohamed Reda Aouad, Sanaa Bardaweel

**Affiliations:** 1 Department of Chemistry, Faculty of Science, Taibah University, Al-Madinah Al-Munawarah 30002, Saudi Arabia; ffs.chem334@gmail.com (F.A.-b.); arshi_84@yahoo.com (A.N.); mo-019-you@hotmail.com (H.Q.U.); s7l_88@hotmail.com (S.A.-S.); aboutasnim@yahoo.fr (M.M.); aouadmohamedreda@yahoo.fr (M.R.A.); 2 Department of Chemistry, Faculty of Sciences, University of Sciences and Technology Mohamed Boudiaf, Laboratoire de Chimie et Electrochimie des Complexes Metalliques (LCECM) USTO-MB, P.O. Box 1505, El M‘nouar, Oran 31000, Algeria; 3 Department of Pharmaceutical Sciences, School of Pharmacy, University of Jordan, Amman 11942, Jordan

**Keywords:** ionic liquids, imidazole, benzothiazole, anticancer, ionic liquids, ADME

## Abstract

A focused array of green imidazolium ionic liquids (ILs) encompassing benzothiazole ring and amide linkage were designed and synthesized using quaternization and metathesis protocols. The synthesized ILs have been fully characterized by usual spectroscopic methods and screened for their anticancer activities against human cancer cell lines originating from breast and colon cancers. Collectively, our biological data demonstrate that the newly synthesized series has variable anticancer activities in the examined cancer types. The synthesized ILs **8**, **10** and **21–29** comprising the methyl and methyl sulfonyl benzothiazole ring emerged as the most potent compounds with promising antiproliferative activities relative to their benzothiazole ring counterparts. Furthermore, the mechanism underlying the observed anticancer activity was investigated. The most active compound **22** appears to exert its anticancer effect through apoptosis dependent pathway in breast cancer cells. Interestingly, compound **22** has also shown good in silico absorption (81.75%) along with high gastro-intestinal absorption as per ADME predictions.

## 1. Introduction

Cancer is one of the leading causes of death, globally, and thus poses a significant impact on worldwide health. Nowadays, major emphasis of drug discovery has been shifted towards the development of novel anticancer agents that reduce toxicity related to the currently available chemotherapies and those targeted at evading tumor resistance mechanisms [1].

Recently, ionic liquids (ILs) have emerged as a new class of compounds with distinctive features supporting the investigation into their potential biological activities [2]. Owing to their nonvolatile, nonflammable, and good water solubility characters, ILs are considered as environmentally friendly, green-chemicals, and thus innovative therapies involving ILs as a drug delivery platform are being explored [3]. In contrast to the typical molecular compounds, the physiochemical properties of ILs can be skillfully adjusted to tune their toxicity while maintaining essential properties required for the desired therapeutic application [4,5].

Nowadays, enormously increasing challenges are facing the pharmaceutical industry concerning the discovery of novel, safe and effective chemical entities for subsequent therapeutic applications [6,7]. Nearly about half of the currently used medicines are administered as salts posing many disadvantages that are basically related to the polymorphism phenomena of drugs [8].

A promising avenue for the pharmaceutical industry has arisen with the development of ILs as druggable moieties in the liquid state. This could assist resolving the dilemma related to the use of drug in its solid form and possibly will enhance the sustainability of drug synthesis [9]. Additionally, due to the good aqueous solubility of ILs, an increase in bioavailability and thus enhancement of their therapeutic activity is anticipated [7,8].

For the foretasted reasons, and over the last few years, a growing consideration has been granted to ILs as potential anticancer agents with desirable features [6,7,8,10]. Interestingly, several reported studies on the activity of ILs as anticancer agents have proposed new perspectives to the potential use of ILs [5,11,12,13,14,15]. Notably, imidazolium based-ionic liquids were found to be the most relevant class of ionic liquids showing fascinating anticancer activity against different cancer cells [16,17,18,19].

As continuation of our interest on the synthesis of bioactive ionic liquids [20,21,22,23,24,25,26,27,28], we report the anti-cancer activity evaluation of a series of imidazolium ionic liquids based amide and benzothiazole conjugate against two model cancer cell lines, HCT 116 and T47D characterizing colon cancer and breast cancer, respectively. The cancer types that were investigated represent the most prevalent types of cancer worldwide [29]. In addition, in silico physicochemical and pharmacokinetic/ADME (absorption, distribution, metabolism and excretion) studies were undertaken in order to theoretically predict their behavior to be a drug candidate. To the best of our knowledge, this is the first report in literature that explores the anticancer activity of such imidazolium ionic liquids.

## 2. Results and Discussion

### 2.1. Chemistry

**Scheme 1:**
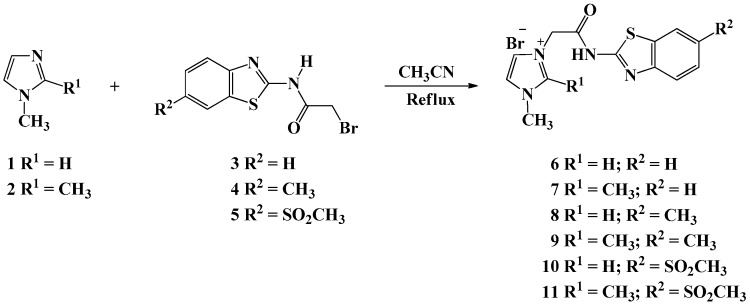
Synthesis of halogenated imidazolium ionic liquids tethered benzothiazole moieties **6–11.**

The designing of the target imidazolium ionic liquids (ILs) involved first the quaternization of imidazolyl nitrogen (=N-) to lead the first generation of ILs. Thus, the alkylation of 1-methyl and/or 1,2-dimethylimidazole **1–2** with appropriate un/substituted *N*-(benzo[*d*]thiazol-2-yl)-2-bromoacetamides **3–5** in refluxing acetonitrile for 30 hr furnished on the formation of new halogenated ionic liquids **6–11** in good yields (80–85%) (Scheme 1). It should be noted that the precursors *N*-(benzo[*d*]thiazol-2-yl)-2-bromoacetamides **3–5** were prepared according to our reported procedures [30,31] via base catalyzed nucleophilic acylation of the appropriate un/substituted 2-aminobenzo[*d*]thiazoles with bromoacetyl bromide in the presence of triethylamine at room temperature. 

Initial formation of new generation of the desired ILs **6–11** was unambiguously confirmed by their spectroscopic analysis (Supplementary materials: Figures S1–S109 can be found at www.mdpi.com/xxx/s1). Compound **10** was taken as model to confirm the formation of such compounds. Its IR spectrum showed clearly the appearance of two strong absorption bands at 1690 and 3320 cm^−1^ characteristic of the carbonyl (C=O) and the amide (NH) group, respectively. In the ^1^H NMR spectrum of compound **10**, two diagnostic singlets were recorded near 5.40 and 12.88 ppm belonging to the C**H_2_** and N**H** groups, respectively (Figure 1). Three extra methyl protons of SO_2_C**H_3_** were observed around 2.42 ppm, which is a good evidence of success of the alkylation of the imidazole ring with *N*-(benzo[*d*]thiazol-2-yl)-2-bromoacetamide (**5**).

**Figure 2:**
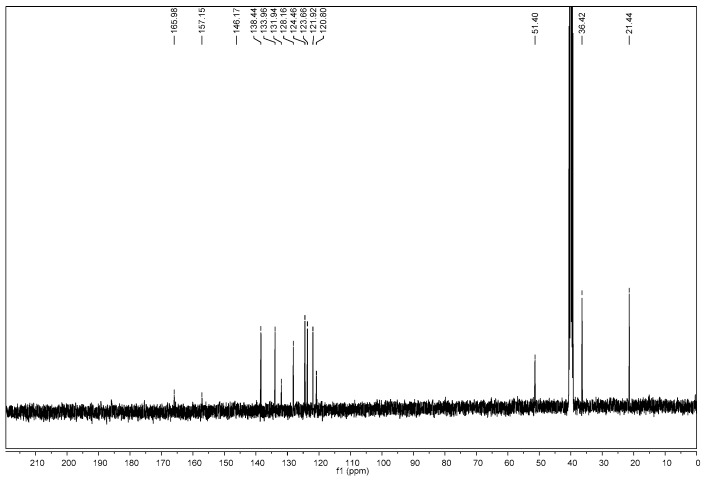
^13^C NMR spectrum of compound **10**.

In addition, the ^13^C NMR spectrum of compound **10** revealed the presence of new signals at 51.40 and 165.98 ppm attributed to the methylene and carbonyl acetamide linkage, respectively (Figure 2), which is another piece of evidence of the incorporation of the acetyl moiety in the imidazole ring. The remaining carbons were observed in their respected area (See experimental part).

**Scheme 2:**
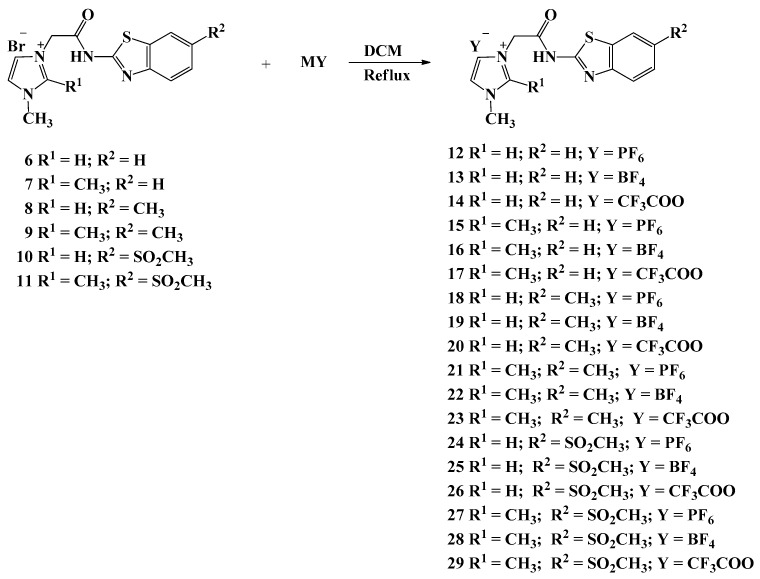
Synthesis of task-specific imidazolium ionic liquids tethered benzothiazole moieties and fluorinated counter anions **12–29**.

The metathesis strategy was also investigated in this work. Thus, the bromide anions in the synthesized ILs **6–11** were exchanged by specific fluorinated anions (BF_4_^−^, PF_6_^−^, CF_3_COO^−^) via their treatment with the appropriate metal salts in boiling dichloromethane (DCM) for 72 h and furnished on the formation of a library of task specific ionic liquids **12–29** in good yields (70–94%) incorporating imidazole ring and un/substituted benzothiazoles linked with an acetamide linkage (Scheme 2). 

The structures of the newly designed task specific ILs **12–29** were established based on their spectroscopic data (^1^H, ^13^C, ^31^P, B, ^19^F NMR and MS). No changes were observed on their ^1^H and ^13^C NMR spectra compared to their corresponding halogenated IL precursors **6–11**. Thus, the significant peaks corresponding to the major anions were obvious in the ^11^B NMR, ^31^P NMR and ^19^F NMR spectra. 

**Figure 3:**
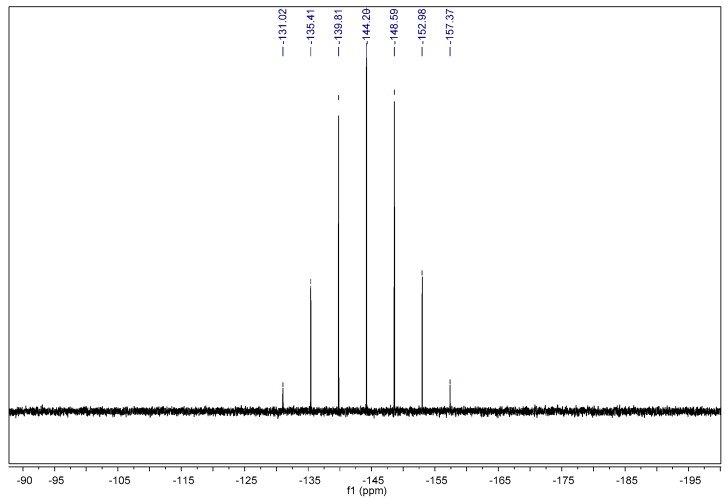
^31^P NMR spectrum of compound **24**.

**Figure 4:**
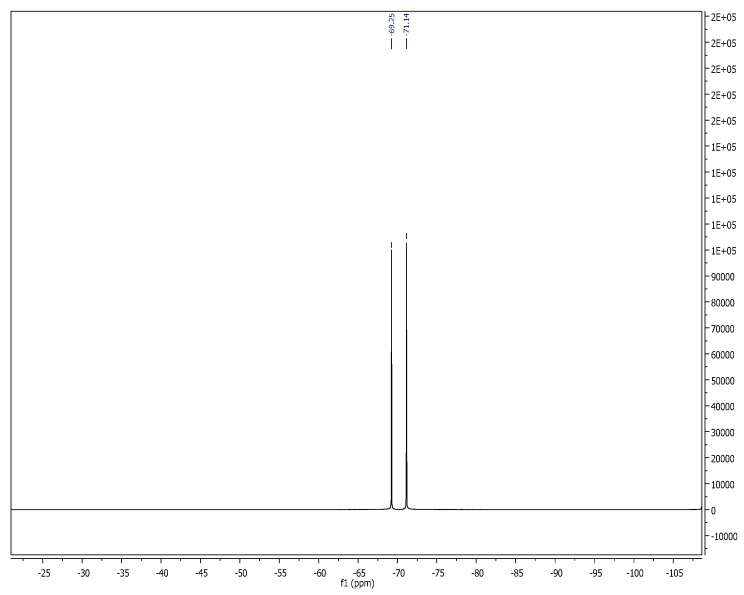
^19^F NMR spectrum of compound **24**.

The ^31^P NMR of IL **24** exhibited a multiplet signal ranging from δ_p_ −157.37 to −131.02 ppm, which clearly confirmed the presence of phosphorus anion in its PF_6_^−^ form (Figure 3). On other hand, in the ^19^F NMR spectrum, the fluorine atoms of PF_6_^−^anion were resonated as a distinct doublet at δ_F_ −69.13 ppm (Figure 4).

**Figure 5:**
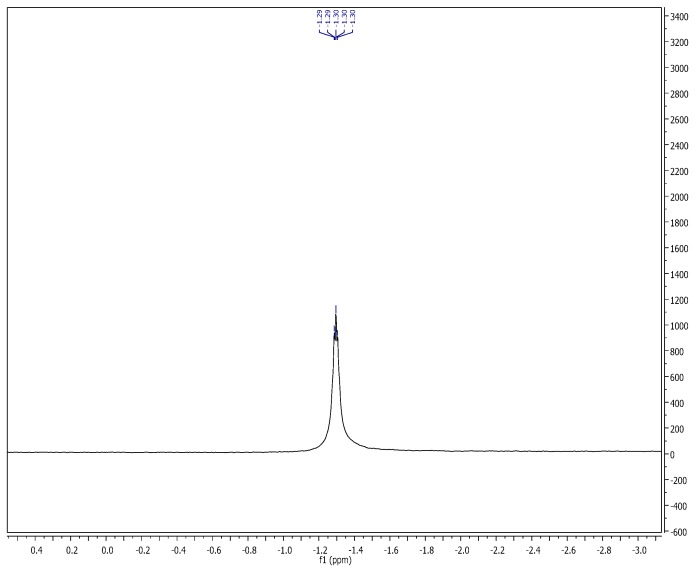
^11^B NMR spectrum of compound **25**.

**Figure 6:**
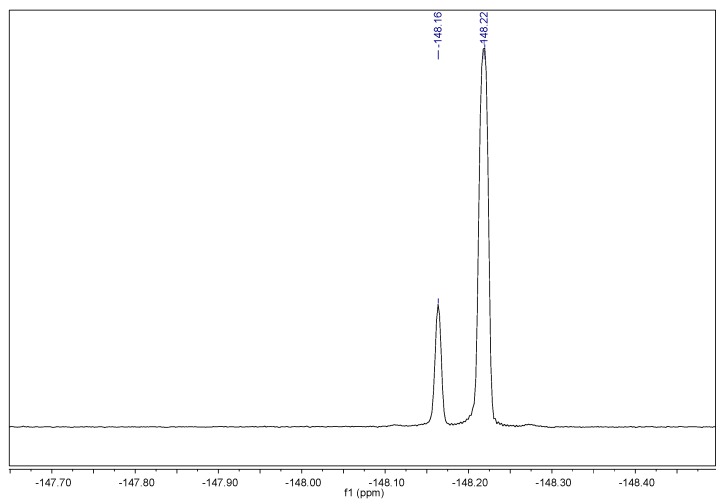
^19^F NMR spectrum of compound **25**.

The anion exchange carried out with the tetrafluoroboron anion (BF_4_^−^) was confirmed by the ^11^B and ^19^F NMR spectra of IL **25** which proved the success of the metathesis reaction (Figure 5). The boron spectrum showed clearly a multiplet between δ_B_ −1.31 to −1.29 ppm, while the fluorine atoms resonated as two doublets at δ_F_ −148.23 and −148.17 ppm in its fluorine spectrum, respectively (Figure 6).

**Figure 7:**
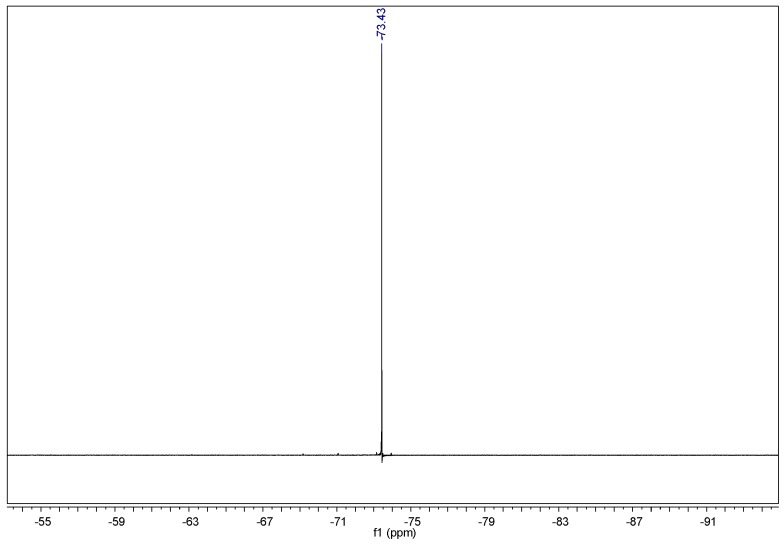
^19^F NMR spectrum of compound **26**.

On the other hand, the presence of trifluoroacetate anion was confirmed by the ^19^F NMR spectrum of IL **26**, its structure was in accordance with its NMR analysis. The spectrum showed the presence of a diagnostic singlet at δ_F_ −73.43 ppm attributed to the fluorine atoms in the C**F**_3_COO^−^ anion (Figure 7).

### 2.2. In Silico Predictions

#### 2.2.1. Physicochemical Properties

Molecular physicochemical parameters of a drug candidate play a significant role in deciding its pharmacokinetic behavior [32]. Therefore, their calculation and measurement aids in prioritizing compounds for screening as efficient drug candidates, and enable premature decisions in drug discovery [33]. The synthesized imidazolium ionic liquids tethered benzothiazole moieties were subjected to in silico physicochemical studies and the results are tabulated in Table 1. 

Various physicochemical features such as the count of specific atom types, number of rotatable bonds, molecular refractivity, lipophilicity and water solubility were recorded. A very functional physiochemical variable, i.e., TPSA (topological polar surface area) is gauged for auditing drug transport properties. Calculations were undertaken to predict % absorption for all the targeted compounds by the reported (%ABS = 109 − (0.345 × TPSA)) [34].

The % absorption ranges from 53.22% to 81.75% in the selected compounds. Most of the compounds demonstrated moderate to good in silico absorption with the highest being 81.75%.

#### 2.2.2. Pharmacokinetic/ADME and Drug Likeness Properties

Pharmacokinetic behavior of the drug compound determines the fate of a therapeutic drug agent in the organism body and it is exploited to predict ADME (absorption, distribution, metabolism and excretion) properties of that drug molecule [35]. Prior assessment of ADME in the drug discovery juncture, drastically foreshorten the fragment of pharmacokinetics-related abortion in the clinical phases [36]. In silico pharmacokinetic predictions of the targeted ILs **6–29** are investigated in this study and recorded in Table 2.

**Figure 8:**
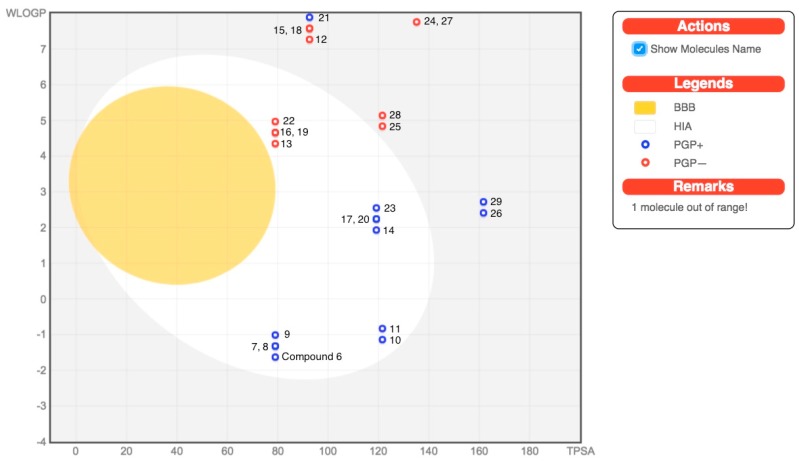
BOILED-Egg diagram of the selected compounds **6–29**.

Most of the tested ILs **6–29** showed high gastro intestinal (GI) absorption and are P-gp (p-glycoprotein) inhibitors. None of the targeted compound was able to permeate through the blood-brain barrier (BBB). The predictions for passive HIA (human gastrointestinal absorption), BBB permeations and P-gp substrates are ventured in an intuitive graphical classification model viz. BOILED-Egg diagram as shown in Figure 8. On the other hand, some of the tested compounds inhibits the Cytochrome P450 isomers while the rest are non-inhibitors. The values for skin permeability coefficient (log Kp; with Kp in cm/s) reckon that the tested compounds possess less skin permeation. 

In addition, drug likeness is another attribute, which furnishes the base for the molecule to be an efficient drug nominee. The Lipinski [37], Ghose [38], Veber [39], Egan [40] and Muegge [41] rules were applied to assess drug likeness to predict whether a compound is likely to be a bioactive according to some important parameters such as molecular weight, LogP, number of HPA and HBD. The number of violations to these rules is documented in Table 3. The Lipinski (Pfizer) filter is the trailblazer rule-of-five (RO5) and except compound **27,** which has one Lipinski’s violation, the rest of all the tested compounds are drug like with bioavailability score of 0.55. The screening process with Ghoserule showed that 10 compounds (**6–12, 15, 18** and **21**) were rejected with one violation and compound **24** and **27** were rejected with two violations while the rest of the compounds abide with the rule. According to the evaluation process with Veber rules, all compounds were drug like except compound **26** and **29** with one violation. Moreover, the evaluation process with Egan rules showed that six compounds (**12, 15, 18, 21, 26** and **29**) were rejected with one violation and two compounds **24** and **27** rejected with two violations while the rest of the sixteen compounds follow the rule. However, in the screening process with Muegge rules, all the compounds meet the criteria of drug likeness evaluation except six compounds (**12, 15, 18, 21, 26** and **29**) with one violation (Table 3).

### 2.3. Biological Study

#### 2.3.1. Anticancer Activity

In consideration of any potential antiproliferative activity that might be associated with the synthesized series, growth inhibition of colon cancer cell lines (HCT-116 and Caco-2) and breast cancer cell lines (T47D and MCF-7) induced by the synthesized imidazolium ILs treatment was evaluated. The results demonstrated that the ILs harboring methyl and methylsulfonyl substituted benzothiazole rings **8**, **10** and **21–29** are relatively more potent than their analogues harboring the unsubstituted benzothiazole ring. IC_50_ values (micromolar) are summarized in Table 4. Relative to the standard anticancer drug Doxorubicin, which demonstrates IC_50_ values between 1–10 uM against the examined cell lines, the synthesized compounds appear to have moderate to weak anticancer activities that need further optimization. Numerous studies have frequently reported that the harmful effects of ILs to cells are connected to the alkyl side chain length, where ILs with C-1 to C-18 alkyl side chains conjugated with different anions (halides) demonstrate augmented toxic outcomes with the increasing alkyl side chain length [2].

#### 2.3.2. The Effects of ILs on cell Apoptosis 

**Figure 9:**
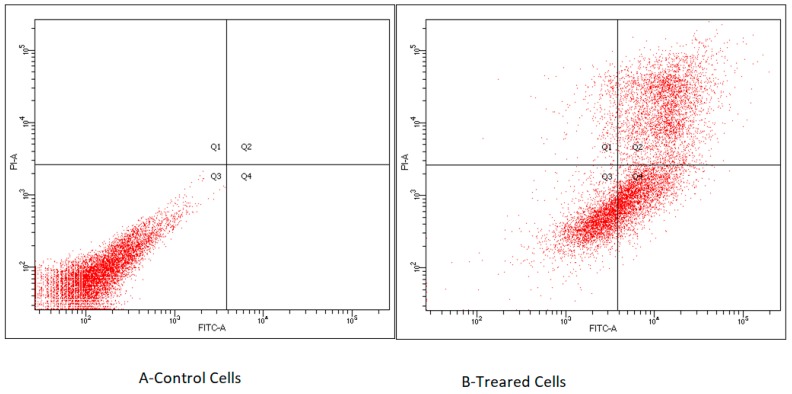
ILs induce apoptosis in T47D breast cancer cells. (**a**) Control cells, (**b**) T47D cells treated with 300µM of **22** for 48 h.

Recently, there have been significant improvements in apprehending the molecular basis of direct cell-mediated cytotoxicity of chemical weapons, which was assisted by both the in vitro and in vivo studies. In this perspective, few literature reports revealed that ionic liquids induced cytotoxicity might be mediated by increasing oxidative stress in the cellular compartment [2] as demonstrated in the QGY-7701 (human hepatocarcinoma) cell line where ILs treatment increased intracellular reactive oxygen species (ROS) generation and diminished the activities of antioxidant enzymes [14]. In this study, the investigation of the underlying mechanism of the observed ILs-induced cytotoxicity, the flow cytometric analysis was carried out. The number of apoptotic cells (T47D) was examined after 48 h incubation period with 300 µM, the results revealed that the IL **22** was the most potent compound. Interestingly, a substantial increase in the number of apoptotic cells after 48 h treatment period was detected (Figure 2). Statistical analysis indicates that the percentages of early apoptotic (29%) and late apoptotic/necrotic (36%) were significantly increased over the control cells suggesting that ILs induced cytotoxicity might be promoted by apoptotic mediated pathways (Figure 9).

## 3. Experimental Section 

### 3.1. Chemical Characterization and Synthesis

Melting points were measured on a Stuart Scientific SMP1 and are uncorrected. TLC was performed on aluminum plates silica gel (Kieselgel, 0.25 mm, 60 F254, Merck, Germany), and spots were visualized by ultraviolet (UV) light absorption using a developing solvent system of ethyl acetate/hexane. SHIMADZU FTIR-8400S spectrometer was used for identification of functional groups in the range of 4000–400 cm^−1^. The NMR spectra were determined on an Advance Bruker (Fällanden, Switzerland) NMR spectrometer at 400 MHz using tetramethylsilane (TMS) as internal standard. High-resolution mass spectroscopy (HRMS) was determined on a Thermo Finnegan MAT 95XP mass spectrometer impact II.

#### 3.1.1. General Procedure for the Quaternization of Imidazoles

To a mixture of un/substituted imidazole **1**, **2** (1 mmol) in acetonitrile (30 mL) was added to the *α-*bromoacetamidebenzothiazole derivatives **3–5** (1 mmol) under stirring. Then, the reaction mixture was heated under reflux for 30 h. After completion of the reaction (as indicated by TLC), the precipitate formed was collected by filtration, washed with water, dried and recrystallized from ethanol to lead the desired imidazolium bromides **6–11**.

#### 3.1.2. 3-(2-(Benzo[*d*]thiazol-2-ylamino)-2-oxoethyl)-1-methyl-1*H*-imidazol-3-ium bromide (**6**). 

Brown crystals in 87%, Mp: 75–76 °C. IR (υ_max_, cm^−1^): 3330 (N-H), 3055 (C-H Ar), 2960 (C-H Al), 1700 (C=O), 1615 (C=N), 1570 (C=C). ^1^H NMR (400 MHz, DMSO-*d*_6_): δ_H_ = 3.94 (s, 3H, NC**H_3_**), 5.42 (s, 2H, NC**H_2_**), 7.34 (t, 1H, *J* =8.0 Hz, Ar-**H**), 7.47 (t, 1H, *J* = 8.0 Hz, Ar-**H**), 7.76–7.80 (m, 3H, Ar-**H**), 8.00 (d, 1H, *J* = 8.0 Hz, Ar-**H**), 9.14 (s, 1H, Ar-**H**), 13.03 (s, 1H, CON**H**). ^13^C NMR (100 MHz, DMSO-*d*_6_): δ_C_ = 36.39 (N**C**H_3_), 51.39 (N**C**H_2_), 121.17; 122.33; 123.66; 124.45; 126.89; 131.78; 138.42; 148.65 (Ar-**C**), 157.95 (**C**=N), 166.09 (**C**=O). HRMS (ESI): 351.9756 [M^+^].

#### 3.1.3. 3-(2-(Benzo[*d*]thiazol-2-ylamino)-2-oxoethyl)-1,2-dimethyl-1*H*-imidazol-3-ium bromide (**7**).

Brown crystals in 85%, Mp: 89–90 °C. IR (υ_max_, cm^−1^): 3365 (N-H), 3045 (C-H Ar), 2920 (C-H Al), 1705 (C=O), 1605 (C=N), 1560 (C=C). ^1^H NMR (400 MHz, DMSO-*d*_6_): δ_H_ = 2.59 (s, 3H, N=CC**H_3_**), 3.83 (s, 3H, NC**H_3_**), 5.02 (s, 2H, NC**H_2_**), 7.19 (t, 2H, *J* = 8.0 Hz, Ar-**H**), 7.61–7.70 (m, 4H, Ar-**H**), 10.77 (s, 1H, CON**H**). ^13^C NMR (100 MHz, DMSO-*d*_6_): δ_C_ = 9.71 (N=C**C**H_3_), 35.40 (N**C**H_3_), 50.54 (N**C**H_2_), 115.92; 116.14; 121.48; 121.56; 122.56; 122.81; 135.15; 135.18; 146.32; 157.56; 159.73 (Ar-**C**, **C**=N), 163.89 (**C**=O). HRMS (ESI): 366.0458 [M^+^].

#### 3.1.4. 1-Methyl-3-(2-((6-methylbenzo[*d*]thiazol-2-yl)amino)-2-oxoethyl)-1*H*-imidazol-3-ium bromide (**8**).

Brown crystals in 85%, Mp: 175–176 °C. IR (υ_max_, cm^−1^): 3350 (N-H), 3080 (C-H Ar), 2980 (C-H Al), 1695 (C=O), 1620 (C=N), 1555 (C=C). ^1^H NMR (400 MHz, DMSO-*d*_6_): δ_H_ = 2.41 (s, 3H, C**H_3_**), 3.88 (s, 0.60H, NC**H_3_**), 3.95 (s, 2.40H, NC**H_3_**), 5.28 (s, 0.25H, NC**H_2_**), 5.41 (s, 1.75H, NC**H_2_**), 7.29 (d, 2H, *J* = 8.0 Hz, Ar-**H**), 7.59–7.79 (m, 4H, Ar-**H**), 9.15 (s, 1H, Ar-**H**), 12.88 (s, 1H, CON**H**). ^13^C NMR (100 MHz, DMSO-*d*_6_): δ_C_ = 21.38; 21.44 (**C**H_3_), 36.42 (N**C**H_3_), 51.40 (N**C**H_2_), 120.80; 121.92; 123.66; 124.46; 128.16; 131.95; 133.95; 138.45; 146.63; 157.07 (Ar-**C**, **C**=N), 165.95 (**C**=O). HRMS (ESI): 366.0546 [M^+^].

#### 3.1.5. 1,2-Dimethyl-3-(2-((6-methylbenzo[*d*]thiazol-2-yl)amino)-2-oxoethyl)-1*H*-imidazol-3-ium bromide (**9**). 

Colorless crystals in 83%, Mp: 88–89 °C. IR (υ_max_, cm^−1^): 3380 (N-H), 3020 (C-H Ar), 2910 (C-H Al), 1700 (C=O), 1610 (C=N), 1565 (C=C). ^1^H NMR (400 MHz, DMSO-*d*_6_): δ_H_ = 2.42 (s, 3H, C**H_3_**), 2.54 (s, 1.60H, N=CC**H_3_**), 2.60 (s, 1.40H, N=CC**H_3_**), 3.72 (s, 1.40H, NC**H_3_**), 3.84 (s, 1.60H, NC**H_3_**), 5.23 (s, 0.60H, NC**H_2_**), 5.34 (s, 0.80H, NC**H_2_**), 5.36 (s, 0.60H, NC**H_2_**), 7.28–7.80 (m, 5H, Ar-**H**), 12.83 (s, 1H, CON**H**). ^13^C NMR (100 MHz, DMSO-*d*_6_): δ_C_ = 9.70; 9.83 (N=C**C**H_3_), 21.17; 21.44 (**C**H_3_), 34.25; 35.40 (N**C**H_3_), 50.43 (N**C**H_2_), 118.58; 120.84; 121.90; 122.69; 122.83; 123.29; 125.32; 128.16; 133.97; 134.87; 146.61, 146.55; 155.34; 159.90 (Ar-**C**, **C**=N), 168.08 (**C**=O). HRMS (ESI): 380.0249 [M^+^].

#### 3.1.6. 1-Methyl-(2-((6-(methylsulfonyl)benzo[*d*]thiazol-2-yl)amino)-2-oxoethyl)-1*H*-imidazol-3-ium bromide (**10**). 

Colorless crystals in 80%, Mp: 82–83 °C. IR (υ_max_, cm^−1^): 3320 (N-H), 3060 (C-H Ar), 2960 (C-H Al), 1690 (C=O), 1605 (C=N), 1570 (C=C). ^1^H NMR (400MHz, DMSO-*d*_6_): δ_H_ = 2.42 (s, 3H, SO_2_C**H_3_**), 3.94 (s, 3H, NC**H_3_**), 5.40 (s, 2H, NC**H_2_**), 7.29 (d, 2H, *J* = 8.0 Hz,Ar-**H**), 7.68 (d, 1H, *J* = 8.0 Hz,Ar-**H**), 7.76–7.79 (m, 3H, Ar-**H**), 9.13 (s, 1H, Ar-**H**), 12.88 (s, 1H, CON**H**). ^13^C NMR (100 MHz, DMSO-*d*_6_): δ_C_ = 21.44 (SO_2_**C**H_3_), 36.42 (N**C**H_3_), 51.40 (N**C**H_2_), 120.80; 121.92; 123.66; 124.46; 128.16; 131.94; 133.96; 138.44; 146.17; 157.15 (Ar-**C**, **C**=N), 165.98 (**C**=O). HRMS (ESI): 429.9741 [M^+^]. 

#### 3.1.7. 1,2-Dimethyl-3-(2-((6-(methylsulfonyl)benzo[*d*]thiazol-2-yl)amino)-2-oxoethyl)-1*H*-imidazol-3-ium bromide (**11**). 

It was obtained as colorless crystals in 82%, Mp: 98–99 °C. IR (υ_max_, cm^−1^): 3345 (N-H), 3060 (C-H Ar), 2950 (C-H Al), 1700 (C=O), 1610 (C=N), 1565 (C=C). ^1^H NMR (400 MHz, DMSO-*d*_6_): δ_H_ = 2.41 (s, 3H, SO_2_C**H_3_**), 2.55 (s, 1.60H, N=CC**H_3_**), 2.61 (s, 1.40H, N=CC**H_3_**), 3.78 (s, 1.60H, NC**H_3_**), 3.84 (s, 1.40H, NC**H_3_**), 5.24 (s, 0.60H, NC**H_2_**), 5.37 (s, 1.40H, NC**H_2_**), 7.29–7.79 (m, 5H, Ar-**H**), 12.85 (s, 1H, CON**H**). ^13^C NMR (100 MHz, DMSO-*d*_6_): δ_C_ = 9.75; 9.84 (N=C**C**H_3_), 21.16; 21.44 (SO_2_**C**H_3_), 35.40; 35.43 (N**C**H_3_), 50.44; 53.12 (N**C**H_2_), 120.77; 121.89; 122.69; 123.35; 125.31; 128.17; 131.91; 133.97; 144.86; 145.77; 146.58; 157.14 (Ar-**C**, **C**=N), 165.71 (**C**=O). HRMS (ESI): 443.9919 [M^+^].

### 3.2. General Procedure for the Metathesis Reaction

To a solution of imidazolium bromides **6–11** (1 mmol) dissolved in acetonitrile (25 mL), appropriate fluorinated metal salts (potassium hexafluorophosphate, sodium tetrafluoroborate and sodium trifluoroacetate) (1.2 mmol) were added under stirring. Then, the reaction mixture was heated under reflux for 72 h. The solid formed was collected by filtration and/or extracted by dichloromethane to lead the desired ionic liquids **12–29**.

#### 3.2.1. 3-(2-(Benzo[*d*]thiazol-2-ylamino)-2-oxoethyl)-1-methyl-1*H*-imidazol-3-ium hexafluorophosphate (**12**). 

Colorless oil in 83%. ^1^H NMR (400 MHz, DMSO-*d*_6_): δ_H_ = 3. 93 (s, 3H, NC**H_3_**), 5.35 (s, 2H, NC**H_2_**), 7.34 (t, 1H, *J* = 8.0 Hz, Ar-**H**), 7.48 (t, 1H, *J* = 8.0 Hz, Ar-**H**), 7.75 (d, 2H, *J* = 12.0 Hz, Ar-**H**), 7.80 (d, 1H, *J* = 8.0 Hz, Ar-**H**), 8.01 (d, 1H, *J* = 8.0 Hz, Ar-**H**), 9.09 (s, 1H, Ar-**H**), 12.94 (s, 1H, CON**H**). ^13^C NMR (100 MHz, DMSO-*d*_6_): δ_C_ = 36.39 (N**C**H_3_), 51.39 (N**C**H_2_), 121.17; 122.33; 123.66; 124.45; 126.89; 131.78; 138.42; 148.65; 157.95 (Ar-**C**, **C**=N), 166.09 (**C**=O). ^31^P NMR (162 MHz, DMSO-*d*_6_) δ_P_ = −157.39 to −131.06 (m, 1P, **P**F_6_). ^19^F NMR (377 MHz, DMSO-*d*_6_): δ_F_ = −69.16 (d, 6F, P**F_6_**). HRMS (ESI): 418.0358 [M^+^].

#### 3.2.2. 3-(2-(Benzo[*d*]thiazol-2-ylamino)-2-oxoethyl)-1-methyl-1*H*-imidazol-3-ium tetrafluoroborate (**13**). 

Colorless oil in 94%. ^1^H NMR (400 MHz, DMSO-*d*_6_): δ_H_ = 3.94 (s, 3H, NC**H_3_**), 5.41 (s, 2H, NC**H_2_**), 7.34 (t, 1H, *J* = 8.0 Hz, Ar-**H**), 7.47 (t, 1H, *J* = 8.0 Hz, Ar-**H**), 7.75–7.81 (m, 3H, Ar-**H**), 8.00 (d, 1H, *J* = 8.0 Hz, Ar-**H**), 9.13 (s, 1H, Ar-**H**), 12.97 (s, 1H, CON**H**). ^13^C NMR (100 MHz, DMSO-*d*_6_): δ_C_ = 36.42 (N**C**H_3_), 51.41 (NCH_2_), 121.20; 122.34; 123.67; 124.44; 126.87; 131.79; 138.42; 149.16; 158.16 (Ar-**C**, **C**=N), 166.04 (**C**=O). ^11^B NMR (128 MHz, DMSO-*d*_6_): δ_B_ = −1.30 to −1.29 (m, 1B, **B**F_4_). ^19^F NMR (377 MHz, DMSO-*d*_6_): δ_F_ = −148.19 and −148.14 (2d, 4F, B**F_4_**). HRMS (ESI): 360.1211 [M^+^].

#### 3.2.3. 3-(2-Benzo[*d*]thiazol-2-ylamino)-2-oxoethyl)-1-methyl-1*H*-imidazol-3-ium 2,2,2-trifluoroacetate (**14**). 

Colorless oil in 93%. ^1^H NMR (400 MHz, DMSO-*d*_6_): δ_H_ = 3. 94 (s, 3H, NC**H_3_**), 5.43 (s, 2H, NC**H_2_**), 7.34 (t, 1H, *J* = 8.0 Hz, Ar-**H**), 7.47 (t, 1H, *J* = 8.0 Hz, Ar-**H**), 7.76–7.79 (m, 3H, Ar-**H**), 8.00 (d, 1H, *J* = 8.0 Hz, Ar-**H**), 9.15 (s, 1H, Ar-**H**), 12.94 (s, 1H, CON**H**). ^13^C NMR (100 MHz, DMSO-*d*_6_): δ_C_ = 36.43 (NCH_3_), 51.42 (NCH_2_), 116.49; 121.19; 122.34; 123.67; 124.44; 126.87; 131.81; 138.42; 145.99; 157.99 (Ar-**C**, **C**=N), 166.05 (**C**=O). ^19^F NMR (377 MHz, DMSO-*d*_6_): δ_F_ = −73.50 (s, 3F, C**F_3_**COO). HRMS (ESI): 386.0589 [M^+^].

#### 3.2.4. 3-(2-(Benzo[*d*]thiazol-2-ylamino)-2-oxoethyl)-1,2-dimethyl-1*H*-imidazol-3-ium hexafluorophosphate (**15**). 

Pale yellow oil in 74%. ^1^H NMR (400 MHz, DMSO-*d*_6_): δ_H_ = 2.59 (s, 3H, C**H_3_**), 3.83 (s, 3H, NC**H_3_**), 5.02 (d, 2H, NC**H_2_**), 7.19 (t, 2H, *J* = 8.0 Hz, Ar-**H**), 7.61–7.70 (m, 4H, Ar-**H**), 10.77 (s, 1H, CON**H**). ^13^C NMR (100 MHz, DMSO-*d*_6_): δ_C_ = 9.71 (N=C**C**H_3_), 35.40 (N**C**H_3_), 50.54 (N**C**H_2_), 115.92; 116.14; 121.48; 121.56; 122.56; 122.81; 135.15; 135.18; 146.32; 157.56 (Ar-**C**, **C**=N), 163.89 (**C**=O).^31^P NMR (162 MHz, DMSO-*d*_6_) δ_P_ = −157.38 to −131.04 (m, 1P, **P**F_6_). ^19^F NMR (377 MHz, DMSO-*d*_6_): δ_F_ = −69.16 (d, 6F, P**F_6_**). HRMS (ESI): 432.1000 [M^+^].

#### 3.2.5. 3-(2-(Benzo[*d*]thiazol-2-ylamino)-2-oxoethyl)-1,2-dimethyl-1*H*-imidazol-3-ium tetrafluoroborate (**16**). 

Yellow oil in 74%. ^1^H NMR (400 MHz, DMSO-*d*_6_): δ_H_ = 2.59 (s, 3H, C**H_3_**), 3.83 (s, 3H, NC**H_3_**), 5.02 (d, 2H, NC**H_2_**), 7.19 (t, 2H, *J* = 8.0 Hz, Ar-**H**), 7.61–7.70 (m, 4H, Ar-**H**), 10.77 (s, 1H, CON**H**). ^13^C NMR (100 MHz, DMSO-*d*_6_): δ_C_ = 9.71 (N=C**C**H_3_), 35.40 (N**C**H_3_), 50.54 (N**C**H_2_), 115.92; 116.14; 121.48; 121.56; 122.56; 122.81; 135.15; 135.18; 146.32; 157.56 (Ar-**C**, **C**=N), 163.89 (**C**=O). ^11^B NMR (128 MHz, DMSO-*d*_6_): δ_B_ = −1.30 to −1.29 (m, 1B, **B**F_4_). ^19^F NMR (377 MHz, DMSO-*d*_6_): δ_F_ = −148.20 and −148.15 (2d, 4F, B**F_4_**). HRMS (ESI): 374.1367 [M^+^].

#### 3.2.6. 3-(2-Benzo[*d*]thiazol-2-ylamino)-2-oxoethyl)-1,2-dimethyl-1*H*-imidazol-3-ium 2,2,2-trifluoroacetate (**17**). 

Colorless oil in 70%. ^1^H NMR (400 MHz, DMSO-*d*_6_): δ_H_ = 2.59 (s, 3H, C**H_3_**), 3.83 (s, 3H, NC**H_3_**), 5.02 (d, 2H, NC**H_2_**), 7.19 (t, 2H, *J* = 8.0 Hz, Ar-**H**), 7.61–7.70 (m, 4H, Ar-**H**), 10.77 (s, 1H, CON**H**). ^13^C NMR (100 MHz, DMSO-*d*_6_): δ_C_ = 9.71 (N=C**C**H_3_), 35.40 (N**C**H_3_), 50.54 (N**C**H_2_), 115.92; 116.14; 121.48; 121.56; 122.56; 122.81; 135.15; 135.18; 146.32; 157.56 (Ar-**C**, **C**=N), 163.89 (**C**=O). ^19^F NMR (377 MHz, DMSO-*d*_6_): δ_F_ = −73.50 (s, 3F, C**F_3_**COO). HRMS (ESI): 400.1013 [M^+^].

#### 3.2.7. 1-Methyl-3-(2-((6-methylbenzo[*d*]thiazol-2-yl)amino)-2-oxoethyl)-1*H*-imidazol-3-ium hexafluorophosphate (**18**). 

Colorless oil in 83%. ^1^H NMR (400 MHz, DMSO-*d*_6_): δ_H_ = 2.42 (t, 3H, C**H_3_**), 3.88 (s, 0.40H, NC**H_3_**), 3.95 (s, 2.60H, NC**H_3_**), 5.28 (s, 0.25H, NC**H_2_**), 5.39 (s, 1.75H, NC**H_2_**), 7.29 (d, 2H, *J* = 8.0 Hz, Ar-**H**), 7.67–7.79 (m, 4H, Ar-**H**), 9.12 (s, 1H, Ar-**H**), 12.88 (s, 1H, CON**H**). ^13^C NMR (100 MHz, DMSO-*d*_6_): δ_C_ = 21.15; 21.43 (**C**H_3_), 36.25; 36.42 (N**C**H_3_), 46.22; 51.39 (N**C**H_2_), 120.80; 121.92; 123.66; 124.46; 128.16; 131.95; 133.95; 138.45; 146.63; 156.93 (Ar-**C**, **C**=N), 165.94 (**C**=O). ^31^P NMR (162 MHz, DMSO-*d*_6_): δ_P_ = −157.36 to −131.02 (m, 1P, **P**F_6_). ^19^F NMR (377 MHz, DMSO-*d*_6_): δ_F_ = −69.22 (d, 6F, P**F_6_**). HRMS (ESI): 432.0870 [M^+^].

#### 3.2.8. 1-Methyl-3-(2-((6-methylbenzo[*d*]thiazol-2-yl)amino)-2-oxoethyl)-1*H*-imidazol-3-ium tetrafluoroborate (**19**). 

Yellow oil in 94%. ^1^H NMR (400 MHz, DMSO-d_6_): δ_H_ = 2.41 (t, 3H, CH_3_), 3.88 (s, 0.40H, NCH_3_), 3.95 (s, 2.60H, NCH_3_), 5.28 (s, 0.25H, NCH_2_), 5.41 (s, 1.75H, NCH_2_), 7.30 (d, 2H, *J* = 8.0 Hz, Ar-H), 7.61–7.79 (m, 4H, Ar-H), 9.15 (s, 1H, Ar-H), 12.88 (s, 1H, CONH). ^13^C NMR (100 MHz, DMSO-d_6_): δ_C_ = 21.09; 21.44 (CH_3_), 36.32; 36.42 (NCH_3_), 51.40 (NCH_2_), 120.80; 121.92; 123.66; 124.46; 128.16; 131.95; 133.95; 138.45; 146.63; 157.07 (Ar-C, C=N), 165.95 (C=O). ^11^B NMR (128 MHz, DMSO-d_6_): δ_B_ = −1.31 to −1.29 (m, 1B, BF_4_). ^19^F NMR (377 MHz, DMSO-d_6_): δ_F_ = −148.17 and −148.12 (2d, 4F, BF_4_). HRMS (ESI): 374.1364 [M^+^].

#### 3.2.9. 1-Methyl-3-(2-((6-methylbenzo[*d*]thiazol-2-yl)amino)-2-oxoethyl)-1*H*-imidazol-3-ium 2,2,2-tri-fluoroacetate (**20**). 

Colorless oil in 93%. ^1^H NMR (400 MHz, DMSO-d_6_): δ_H_ = 2.41 (s, 3H, CH_3_), 3.88 (s, 0.40H, NCH_3_), 3.95 (s, 2.60H, NCH_3_), 5.28 (s, 0.25H, NCH_2_), 5.41 (s, 1.75H, NCH_2_), 7.30 (d, 2H, *J* = 8.0 Hz, Ar-H), 7.61–7.79 (m, 4H, Ar-H), 9.15 (s, 1H, Ar-H), 12.88 (s, 1H, CONH). ^13^C NMR (100 MHz, DMSO-d_6_): δ_C_ = 21.39; 21.44 (CH_3_), 36.37; 36.42 (NCH_3_), 51.40 (NCH_2_), 113.37; 120.80; 121.92; 123.66; 124.46; 128.16; 131.95; 133.95; 138.45; 146.63; 157.07 (Ar-C, C=N), 165.95 (C=O). ^19^F NMR (377 MHz, DMSO-d_6_): δ_F_ = −73.50 (s, 3F, CF_3_COO). HRMS (ESI): 400.0999 [M^+^].

#### 3.2.10. 1,2-Dimethyl-3-(2-((6-methylbenzo[*d*]thiazol-2-yl)amino)-2-oxoethyl)-1*H*-imidazol-3-ium hexafluorophosphate (**21**). 

Colorless oil in 74%. ^1^H NMR (400 MHz, DMSO-d_6_): δ_H_ = 2.42 (s, 3H, CH_3_), 2.54 (s, 1.60H, N=CCH_3_), 2.60 (s, 1.40H, N=CCH_3_), 3.72 (s, 1.40H, NCH_3_), 3.84 (s, 1.60H, NCH_3_), 5.23 (s, 0.60H, NCH_2_), 5.34 (s, 0.80H, NCH_2_), 5.36 (s, 0.60H, NCH_2_), 7.28–7.80 (m, 5H, Ar-H), 12.83 (s, 1H, CONH). ^13^C NMR (100 MHz, DMSO-d_6_): δ_C_ = 9.70; 10.21 (N=CCH_3_), 21.16; 21.44 (CH_3_), 35.33; 35.37 (NCH_3_), 50.40; 53.20 (NCH_2_), 121.90; 122.69; 122.84; 123.44; 125.32; 128.17; 133.98; 134.98; 146.55; 157.35 (Ar-C, C=N), 168.24 (C=O).^31^P NMR (162 MHz, DMSO-d_6_): δ_P_ = −157.38 to −131.03 (m, 1P, PF_6_). ^19^F NMR (377 MHz, DMSO- d_6_): δ_F_ = −69.25 (d, 6F, PF_6_). HRMS (ESI): 446.0657 [M^+^].

#### 3.2.11. 1,2-Dimethyl-3-(2-((6-methylbenzo[*d*]thiazol-2-yl)amino)-2-oxoethyl)-1*H*-imidazol-3-ium tetrafluoroborate (**22**). 

Colorless oil in 74%. ^1^H NMR (400 MHz, DMSO-d_6_): δ_H_ = 2.42 (s, 3H, CH_3_), 2.54 (s, 1.60H, N=CCH_3_), 2.60 (s, 1.40H, N=CCH_3_), 3.72 (s, 1.40H, NCH_3_), 3.84 (s, 1.60H, NCH_3_), 5.23 (s, 0.60H, NCH_2_), 5.34 (s, 0.80H, NCH_2_), 5.36 (s, 0.80H, NCH_2_), 7.28–7.80 (m, 5H, Ar-H), 12.83 (s, 1H, CONH). ^13^C NMR (100 MHz, DMSO-d_6_): δ_C_ = 9.67; 9.87 (N=CCH_3_), 21.16; 21.44 (CH_3_), 35.33; 35.39 (NCH_3_), 50.43; 53.13 (NCH_2_), 120.80; 121.90; 122.43; 122.57; 123.46; 125.32; 128.15; 129.06; 131.93; 133.89; 133.95; 134.95; 135.02; 145.79; 146.56; 157.13 (Ar-C, C=N), 168.08 (C=O). ^11^B NMR (128 MHz, DMSO-d_6_): δ_B_ = −1.30 to −1.29 (m, 1B, BF_4_). ^19^F NMR (377 MHz, DMSO-d_6_): δ_F_ = −148.17 and −148.12 (2d, 4F, BF_4_). HRMS (ESI): 388.1078 [M^+^].

#### 3.2.12. 1,2-Dimethyl-3-(2-((6-methylbenzo[*d*]thiazol-2-yl)amino)-2-oxoethyl)-1*H*-imidazol-3-ium 2,2,2-trifluoroacetate (23). 

Yellow oil in 70%. ^1^H NMR (400 MHz, DMSO-d_6_): δ_H_ = 2.42 (s, 3H, CH_3_), 2.51 (s, 2H, N=CCH_3_), 2.61 (s, 1H, N=CCH_3_), 3.37 (bs, 1H, NCH_3_overlapped with DMSO-d_6_), 3.79 (s, 1H, NCH_3_), 3.85 (s, 1H, NCH_3_), 5.26 (s, 0.60H, NCH_2_), 5.36 (s, 0.62H, NCH_2_), 5.37 (s, 0.80H, NCH_2_), 7.28–7.80 (m, 5H, Ar-H), 12.88 (s, 1H, CONH). ^13^C NMR (100 MHz, DMSO-d_6_): δ_C_ = 9.70; 9.94 (N=CCH_3_), 21.16; 21.44 (CH_3_), 35.36; 35.40 (NCH_3_), 50.43; 53.18 (NCH_2_), 113.31; 121.89; 122.70; 122.84; 125.32; 128.14; 133.93; 135.02; 146.55; 157.34 (Ar-C, C=N), 165.08 (C=O). ^19^F NMR (377 MHz, DMSO-d_6_): δ_F_ = −73.43 (s, 3F, CF_3_COO). HRMS (ESI): 414.0992 [M^+^].

#### 3.2.13. 1-Methyl-3-(2-((6-(methylsulfonyl)benzo[*d*]thiazol-2-yl)amino)-2-oxoethyl)-1*H*-imidazol-3-ium hexafluorophosphate (**24**). 

Yellow crystals in 85%, Mp: 78–80 °C. ^1^H NMR (400 MHz, DMSO-d_6_): δ_H_ = 2.42 (s, 3H, SO_2_CH_3_), 3.95 (s, 3H, NCH_3_), 5.39 (s, 2H, NCH_2_), 7.29 (d, 1H, *J* = 8.0 Hz, Ar-H), 7.68 (d, 1H, J =8.0 Hz, Ar-H), 7.76–7.79 (m, 3H, Ar-H), 9.12 (s, 1H, Ar-H), 12.88 (s, 1H, CONH). ^13^C NMR (100 MHz, DMSO-d_6_): δ_C_ = 21.44 (SO_2_CH_3_), 36.41 (NCH_3_), 51.40 (NCH_2_), 120.82; 121.92; 123.67; 124.47; 128.16; 131.97; 133.95; 138.45; 146.42; 156.66 (Ar-C, C=N), 165.94 (C=O). ^31^P NMR (162 MHz, DMSO-d_6_): δ_P_ = −157.37 to −131.02 (m, 1P, PF_6_). ^19^F NMR (377 MHz, DMSO-d_6_): δ_F_ = −69.13 (d, 6F, PF_6_). HRMS (ESI): 496.0195[M^+^].

#### 3.2.14. 1-Methyl-3-(2-((6-(methylsulfonyl)benzo[*d*]thiazol-2-yl)amino)-2-oxoethyl)-1*H*-imidazol-3-ium tetrafluoroborate (**25**). 

Colorless crystals in 78%, Mp: 88–89 °C. ^1^H NMR (400 MHz, DMSO-d_6_): δ_H_ = 2.42 (s, 3H, SO_2_CH_3_), 3.95 (s, 3H, NCH_3_), 5.41 (s, 2H, NCH_2_), 7.29 (d, 1H, *J* = 8.0 Hz, Ar-H), 7.68 (d, 1H, *J* = 8.0 Hz, Ar-H), 7.77–7.79 (m, 3H, Ar-H), 9.14 (s, 1H, Ar-H), 12.88 (s, 1H, CONH). ^13^C NMR (100 MHz, DMSO-d_6_): δ_C_ = 21.44 (SO_2_CH_3_), 36.42 (NCH_3_), 51.40 (NCH_2_), 120.84; 121.92; 123.67; 124.46; 128.15; 131.99; 133.95; 138.45; 146.84; 156.94 (Ar-C, C=N), 165.92 (C=O). ^11^B NMR (128 MHz, DMSO-d_6_): δ_B_ = −1.31 to −1.29 (m,1B, BF_4_). ^19^F NMR (377 MHz, DMSO-d_6_): δ_F_ = −148.23 and −148.17 (2d, 4F, BF_4_). HRMS (ESI): 438.0596[M^+^].

#### 3.2.15. 1-Methyl-3-(2-((6-(methylsulfonyl)benzo[*d*]thiazol-2-yl)amino)-2-oxoethyl)-1*H*-imidazol-3-ium 2,2,2-trifluoroacetate (**26**). 

Yellow crystals in 70%, Mp: 85–86 °C. ^1^H NMR (400 MHz, DMSO-d_6_): δ_H_ = 2.41 (s, 3H, SO_2_CH_3_), 3.95 (s, 3H, NCH_3_), 5.44 (s, 2H, NCH_2_), 7.28 (d, 1H, *J* = 8.0 Hz, Ar-H), 7.68 (d, 1H, *J* = 8.0 Hz, Ar-H), 7.79–7.81 (m, 3H, Ar-H), 9.18 (s, 1H, Ar-H), 12.89 (s, 1H, CONH). ^13^C NMR (100 MHz, DMSO-d_6_): δ_C_ = 21.44 (SO_2_CH_3_), 36.43 (NCH_3_), 51.42 (NCH_2_), 114.69; 120.81; 121.92; 123.66; 124.45; 128.14; 131.98; 133.93; 138.44; 147.28; 157.07 (Ar-C, C=N), 165.89 (C=O). ^19^F NMR (377 MHz, DMSO-d_6_): δ_F_ = −73.43 (s, 3F, CF_3_COO). HRMS (ESI): 464.0736 [M^+^].

#### 3.2.16. 1,2-Dimethyl-3-(2-((6-(methylsulfonyl)benzo[*d*]thiazol-2-yl)amino)-2-oxoethyl)-1*H*-imidazol-3-ium hexafluorophosphate (**27**). 

Pale yellow crystals in 86%, Mp: 92–94 °C. ^1^H NMR (400 MHz, DMSO-d_6_): δ_H_ = 2.43 (s, 3H, SO_2_CH_3_), 2.51 (s, 1.60H, N=CCH_3_), 2.61 (s, 1.40H, N=CCH_3_), 3.79–3.85 (m, 3H, NCH_3_), 5.24 (s, 0.60H, NCH_2_), 5.37 (s, 1.40H, NCH_2_), 7.29–7.79 (m, 5H, Ar-H), 12.85 (bs, 1H, CONH). ^13^C NMR (100 MHz, DMSO-d_6_): δ_C_ = 9.66; 9.80 (N=CCH_3_), 21.16; 21.44 (SO_2_CH_3_), 35.37 (NCH_3_), 50.42; 53.27 (NCH_2_), 120.80; 121.90; 122.69; 122.84; 125.35; 128.17; 129.08; 131.95; 133.98; 145.79; 146.58; 157.19 (Ar-C, C=N), 165.71 (C=O). ^31^P NMR (162 MHz, DMSO-d_6_): δ_P_ = −157.32 to −130.99 (m, 1P, PF_6_). ^19^F NMR (377 MHz, DMSO-d_6_): δ_F_ = −69.14 (d, 6F, PF_6_). HRMS (ESI): 510.0570 [M^+^].

#### 3.2.17. 1,2-Dimethyl-3-(2-((6-(methylsulfonyl)benzo[*d*]thiazol-2-yl)amino)-2-oxoethyl)-1*H*-imidazol-3- ium tetrafluoroborate (**28**). 

Colorless crystals in 83%, Mp: 100–102 °C. ^1^H NMR (400 MHz, DMSO-d_6_): 2.42 (s, 3H, SO_2_CH_3_), 2.51 (s, 1.60H, N=CCH_3_), 2.61 (s, 1.40H, N=CCH_3_), 3.78–3.84 (m, 3H, NCH_3_), 5.24 (s, 0.60H, NCH_2_), 5.35 (s, 0.80H, NCH_2_), 5.37 (s, 0.60H, NCH_2_), 7.29–7.79 (m, 5H, Ar-H), 12.86 (s, 1H, CONH).^13^C NMR (100 MHz, DMSO-d_6_): δ_C_ = 9.62; 9.87 (N=CCH_3_), 21.16; 21.44 (SO_2_CH_3_), 35.39 (NCH_3_), 50.43; 53.24 (NCH_2_), 120.80; 121.90; 122.69; 122.84; 125.35; 128.17; 129.08; 131.95; 133.98; 145.79; 146.58; 157.19 (Ar-C, C=N), 168.08 (C=O). ^11^B NMR (128 MHz, DMSO-d_6_): δ_B_ = −1.30 to −1.28 (m, 1B, BF_4_). ^19^F NMR (377 MHz, DMSO-d_6_): δ_F_ = -148.21 and -148.15 (2d, 4F, BF_4_). HRMS (ESI): 452.0534[M^+^].

#### 3.2.18. -1,2-Dimethyl-3-(2-((6-(methylsulfonyl)benzo[*d*]thiazol-2-yl)amino)-2-oxoethyl)-1*H*-imidazol-3-ium 2,2,2-trifluoroacetate (**29**). 

Yellow crystals in 75%, Mp: 86–88 °C. ^1^H NMR (400 MHz, DMSO-d_6_): δ_H_ = 2.41 (s, 3H, SO_2_CH_3_), 2.51 (s, 2.10H, N=CCH_3_), 2.59 (s, 0.90H, N=CCH_3_), 3.77 (s, 1.70H, NCH_3_), 3.88 (s, 1.30H, NCH_3_), 5.22 (s, 0.80H, NCH_2_), 5.36 (s, 1.20H, NCH_2_), 7.29–7.79 (m, 5H, Ar-H), 12.59 (s, 1H, CONH).^13^C NMR (100 MHz, DMSO-d_6_): δ_C_ = 9.68; 9.86(N=CCH_3_), 21.15; 21.43 (SO_2_CH_3_), 35.34; 35.38 (NCH_3_), 50.50; 53.41 (NCH_2_), 113.26; 121.91; 122.55; 123.46; 125.31; 128.16; 129.09; 131.96; 133.91; 134.98; 145.77; 157.19 (Ar-C, C=N), 168.10 (C=O). ^19^F NMR (377 MHz, DMSO-d_6_): δ_F_ = −73.50 (s, 3F, CF_3_COO). HRMS (ESI): 478.0708[M^+^].

### 3.3. Method of Computation: In Silico Study

Physicochemical, pharmacokinetic/ADME and drug likeness properties of the targeted compounds **6–29** were analyzed using SwissADME web interface which is developed and maintained by the Molecular Modeling Group of the Swiss Institute of Bioinformatics (http://www.sib.swiss) [42]. 2D structural models were drawn directly into the Marvin JS sketcher window and then converted to the SMILES format. SwissADME utilizes the SMILES format to predict these properties.

### 3.4. Biological Study 

#### 3.4.1. Anticancer Activity

##### Cell Line and Culture Conditions

The HCT-116 human colon carcinoma cell line and the T47D human ductal breast epithelial tumor cell line were purchased from the American Type Culture Collection (ATCC, Rockville, MD, USA). Cells were cultured in Dulbecco’s modified eagle medium (DMEM) (Invitrogen, USA) containing 10% heat inactivated fetal bovine serum (HI-FBS) (Invitrogen), 2 mmol L^–1^ of L-glutamine, 50 U mL^–1^ of penicillin and 50 μg mL^–1^ of streptomycin. Cells were cultured in an atmosphere of 5% CO_2_ and 95% relative humidity at 37 °C. 

##### In Vitro Cytotoxicity

In vitro appraisal of the antiproliferative activities linked to the synthesized compounds were accomplished in accordance to the protocol described in the ISO 10993-5 guide [43]. Concisely, cells were seeded at seeding density of 1 × 10^4^ cells per well in 96-well plates and incubated to allow adhesion for 24 h. The examined compounds were dissolved in DMSO and subsequently diluted in culture media. The final concentration of DMSO was kept constant in all treatment groups within a given experiment and never exceeded 1%. Three triplicates of each concentration for all tested compounds were performed in three independent assays for a total of nine triplicates. 

DMEM samples were employed as negative controls, while Doxorubicin was utilized as a positive control. Primarily, the culture medium in each well was replaced with 100 µL of either test or control solutions, followed by incubation for 48 h at 37 °C in a 5% CO_2_ incubator. When the exposure period ended, the MTT assay was carried out as previously described [44]. Briefly, viable cell count was resolved using the 3-(4,5-dimethylthiazol-2yl)-2,5-diphenyl tetrazolium bromide (MTT) colorimetric assay. The yellow tetrazolium dye [MTT, 3-(4,5-dimethylthiazol-2-yl)-5-(3-carboxymethoxyphenyl)-2-(4-sulfophenyl)-2*H*-tetrazolium, inner salt] was reduced by metabolically active cells into an intracellular purple formazan product. The number of viable cells in the culture is directly proportional to the quantity of the formazan product, as determined by the absorbance at 490 nm. Cell viability was calculated relying on the measured absorbance relative to the absorbance of the cells exposed to the negative controls, which represented 100% cell viability.

##### Flow Cytometry Analysis:

The Annexin V-FITC Apoptosis Detection Kit was used according to the manufacturer’s protocols with minor modifications, as previously described [44]. T47D cells were seeded into 6-well plates at 5 × 10^5^ cells/dish density and treated for 48 h with 300 µM of AZ6. Doxorubicin was used as a positive control. Cells were maintained at 37 °C in a 5% CO_2_ incubator. For the apoptosis analysis, the cells were treated with trypsin for 7 min, washed with cold PBS and then resuspended in 500 µL cold Binding Buffer. Afterward, cells were stained using the Annexin V-FITC reaction reagent (5 μL of Annexin V-FITC, 5 μL of propidium iodide) at 37 °C for 30 min protected from light. The stained cells were analyzed using the flow cytometry analysis.

## 4. Conclusions

A focused library of new library of imidazolium ionic liquids appended substituted benzothiazole were designed, synthesized and well characterized using full spectroscopic analysis. The resulted compounds were evaluated for their in vitro antiproliferative activities. Compound **22** emerged as the most active against all examined cell lines with IC_50_ values of two digits micromolar. The antiproliferative activity associated with the most potent compound appeared to be mediated through an apoptotic dependent pathway in breast cancer cells.

These findings were further endorsed by in silico studies as most of the compounds revealed moderate to good in silico absorption. The ADME predictions unveiled that most of the targeted compounds have manifested high GI absorption and are P-gp inhibitors. They came out to be non-BBB and non-skin permeable. Few of the examined compounds were forecasted to be inhibitors of Cytochrome P450 isomers. Generally, the compounds containing BF_4_^−^ and CF_3_COO^−^ counter anions were predicted to be drug like except **26** and **29** which displayed one violation each for the Veber, Egan and Muegge rule.

## Figures and Tables

**Figure 1:**
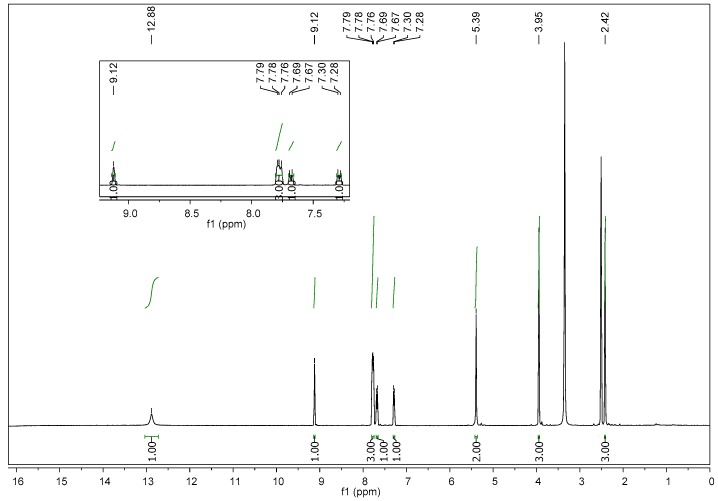
^1^H NMR spectrum of compound **10**.

**Table 1:** Physicochemical properties of the selected compounds **6–29**.

Comp.No.	Fraction Csp3 ^a^	No. of Rotatable Bonds	HBA ^b^	HBD ^c^	iLogP ^d^	Molar Refractivity	Log *S* ^e^	TPSA ^f^	In-Silico % Absorption
**6**	0.15	4	2	1	−5.18	85.22	S	79.04	81.75
**7**	0.21	4	2	1	−2.34	90.19	S	79.04	81.75
**8**	0.21	4	2	1	−4.45	90.19	S	79.04	81.75
**9**	0.27	4	2	1	−2.88	95.16	MS	79.04	81.75
**10**	0.21	5	4	1	−5.71	98.32	S	121.56	67.06
**11**	0.27	5	4	1	−4.77	103.28	MS	121.56	67.06
**12**	0.15	4	8	1	0	89.86	S	92.63	77.04
**13**	0.15	4	6	1	0	86.48	S	79.04	81.75
**14**	0.2	5	7	1	−4.3	88.05	S	119.17	67.89
**15**	0.21	4	8	1	0	94.83	S	92.63	77.04
**16**	0.21	4	6	1	0	91.45	S	79.04	81.75
**17**	0.25	5	7	1	−3.36	93.01	S	119.17	67.89
**18**	0.21	4	8	1	0	94.83	S	92.63	77.04
**19**	0.21	4	6	1	0	91.45	S	79.04	81.75
**20**	0.25	5	7	1	−3.57	93.01	S	119.17	67.89
**21**	0.27	4	8	1	0	99.8	MS	92.63	77.04
**22**	0.27	4	6	1	0	96.41	MS	79.04	81.75
**23**	0.29	5	7	1	−4.17	97.98	MS	119.17	67.89
**24**	0.21	5	10	1	0	102.96	S	135.15	62.37
**25**	0.21	5	8	1	0	99.58	S	121.56	67.06
**26**	0.25	6	9	1	−5.2	101.14	S	161.69	53.22
**27**	0.27	5	10	1	0	107.92	MS	135.15	62.37
**28**	0.27	5	8	1	0	104.54	MS	121.56	67.06
**29**	0.29	6	9	1	−4.83	106.11	MS	161.69	53.22

^a^ The ratio of sp^3^ hybridized carbons over the total carbon count of the molecule; ^b^ number of hydrogen bond acceptors; ^c^ number of hydrogen bond donors; ^d^ lipophilicity; ^e^ water solubility (SILICOS-IT[ S = Soluble, MS = Moderately Soluble]); ^f^ topological polar surface area (Å^2^).

**Table 2:** Pharmacokinetic/ADME (absorption, distribution, metabolism and excretion) properties of the selected compounds **6–29**.

Comp. No	Pharmacokinetic/ADME Properties								
	GI Abs ^a^	BBB Permeant ^b^	P-Gpsubstrate ^c^	CYP1A2 Inhibitor ^d^	CYP2C19 Inhibitor ^e^	CYP2C9 Inhibitor ^f^	CYP2D6 Inhibitor ^g^	CYP3A4 Inhibitor ^h^	Log K_p_^i^
**6**	High	No	Yes	No	No	No	No	No	−6.39
**7**	High	No	Yes	No	No	No	No	No	−6.19
**8**	High	No	Yes	No	No	No	No	No	−6.22
**9**	High	No	Yes	No	No	No	No	No	−6.02
**10**	High	No	Yes	No	No	No	No	No	−7.4
**11**	High	No	Yes	No	No	No	No	No	−7.21
**12**	Low	No	No	No	Yes	No	No	Yes	−5.19
**13**	Low	No	No	No	Yes	No	No	Yes	−4.99
**14**	High	No	No	No	No	No	No	No	−5.54
**15**	High	No	No	No	No	No	No	No	−5.34
**16**	High	No	Yes	No	No	No	No	No	−6.64
**17**	High	No	Yes	No	No	No	No	No	−6.44
**18**	Low	No	No	No	Yes	No	No	Yes	−5.02
**19**	High	No	No	No	No	No	No	No	−5.37
**20**	High	No	Yes	No	No	No	No	No	−6.46
**21**	Low	No	Yes	No	Yes	No	No	Yes	−4.82
**22**	High	No	No	No	No	No	No	No	−5.17
**23**	High	No	Yes	No	No	No	No	No	−6.26
**24**	Low	No	No	No	No	No	No	Yes	−6.21
**25**	Low	No	No	No	No	No	No	No	−6.56
**26**	Low	No	Yes	No	No	No	No	No	−7.66
**27**	Low	No	No	No	No	No	No	Yes	−6.01
**28**	Low	No	No	No	No	No	No	No	−6.36
**29**	Low	No	Yes	No	No	No	No	No	−7.46

^a^ Gastro Intestinal absorption, ^b^ Blood Brain Barrier permeant, ^c^ P-glycoprotein substrate, ^d^ CYP1A2: Cytochrome P450 family 1 subfamily A member 2 (PDB:2HI4), ^e^ CYP2C19: Cytochrome P450 family 2 subfamily C member 19 (PDB:4GQS), ^f^ CYP2C9: Cytochrome P450 family 2 subfamily C member 9 (PDB:1OG2), ^g^ CYP2D6: Cytochrome P450 family 2 subfamily D member 6 (PDB:5TFT), ^h^ CYP3A4: Cytochrome P450 family 3 subfamily A member 4 (PDB:4K9T), ^I^ Skin permeation in cm/s.

**Table 3:** Drug likeness predictions of the selected compounds **6–29**.

Comp. No.	Lipinski Violations	Ghose Violations	Veber Violations	Egan Violations	Muegge Violations	Bioavailability Score
**6**	0	1	0	0	0	0.55
**7**	0	1	0	0	0	0.55
**8**	0	1	0	0	0	0.55
**9**	0	1	0	0	0	0.55
**10**	0	1	0	0	0	0.55
**11**	0	1	0	0	0	0.55
**12**	0	1	0	1	1	0.55
**13**	0	0	0	0	0	0.55
**14**	0	0	0	0	0	0.55
**15**	0	1	0	1	1	0.55
**16**	0	0	0	0	0	0.55
**17**	0	0	0	0	0	0.55
**18**	0	1	0	1	1	0.55
**19**	0	0	0	0	0	0.55
**20**	0	0	0	0	0	0.55
**21**	0	1	0	1	1	0.55
**22**	0	0	0	0	0	0.55
**23**	0	0	0	0	0	0.55
**24**	0	2	0	2	0	0.55
**25**	0	0	0	0	0	0.55
**26**	0	0	1	1	1	0.55
**27**	1	2	0	2	0	0.55
**28**	0	0	0	0	0	0.55
**29**	0	0	1	1	1	0.55

**Table 4:** Growth inhibition IC_50_ (µM) of HCT-116 and T47D cell lines after 48 h exposure time. Data are expressed as mean ±SD of three independent experiments.

Comp. N	HCT	Caco-2	T47D	MCF-7
**6**	>500	>500	>500	>500
**7**	>500	>500	>500	>500
**8**	143 ± 5	122 ± 3	162 ± 6	153 ± 8
**9**	210 ± 8	189 ± 7	230 ± 11	194 ± 6
**10**	246 ± 12	229 ± 8	237 ± 12	241 ± 7
**11**	187 ± 8	178 ± 11	198 ± 7	193 ± 6
**12**	>500	>500	>500	>500
**13**	>500	>500	>500	>500
**14**	>500	>500	>500	>500
**15**	>500	>500	>500	>500
**16**	>500	>500	>500	>500
**17**	>500	>500	>500	>500
**18**	183 ± 7	179 ± 5	188 ± 9	177 ± 4
**19**	132 ± 9	112 ± 5	139 ± 7	125 ± 6
**20**	190 ± 11	181 ± 9	187 ± 9	188 ± 6
**21**	168 ± 7	156 ± 3	167 ± 8	143 ± 7
**22**	98 ± 9	88 ± 4	115 ± 5	89 ± 3
**23**	149 ± 6	137 ± 5	154 ± 7	155 ± 4
**24**	246 ± 9	260 ± 11	293 ± 5	287 ± 10
**25**	211 ± 5	199 ± 4	200 ± 8	205 ± 6
**26**	210 ± 9	219 ± 3	213 ± 7	218 ± 4
**27**	239 ± 11	248 ± 8	241 ± 9	251 ± 7
**28**	186 ± 7	179 ± 7	178 ± 4	168 ± 6
**29**	174 ± 10	169 ± 7	167 ± 8	171 ± 9

## Supplementary Materials

Supplementary materials can be found at https://www.mdpi.com/1422-0067/20/12/2865/s1.

## Abbreviations

ILs	Ionic Liquids
ADME	Absorption, Distribution, Metabolism and Excretion
DCM	Dichloromethane
TPSA	Topological Polar Surface Area
ABS	Absorption
HBA	Number of hydrogen bond acceptor
HBD	Number of hydrogen bond donor
GI	Gastro intestinal
P-gp	P-glycoprotein
BBB	Blood-brain barrier
HIA	Human gastrointestinal absorption
RO5	Rule-of-five
ROS	Reactive oxygen species
HRMS	High resolution mass spectroscopy
TMS	Tetramethylsilane
DMSO	Dimethylsufoxide
S	Singlet
D	Doublet
T	Triplet
M	Multiplet
