# Supplementary material for: A Profile of the In Vitro Anti-Tumor Activity and In Silico ADME Predictions of Novel Benzothiazole Amide-Functionalized Imidazolium Ionic Liquids
Source: Int J Mol Sci. 2019 Jun 12;20(12):2865. doi: 10.3390/ijms20122865 (PMC6627815; doi:10.3390/ijms20122865)
Supplement: Supplementary file 1 [file ijms-20-02865-s001.pdf]

A Profile of the *in vitro* Anti-tumor Activity Against Breast and Colon Cancer Cell Lines of novel specific ionic liquids based imidazole, benzothiazole conjugates

Fawzia F. Al-blewi, Nadjet Rezki,* Arshi Naqvi, Husna A. Qutb eddn, Salsabeel A. Al-Sodies, Mouslim Messali, Mohamed R. Aouad, Sanaa K. Bardaweel*

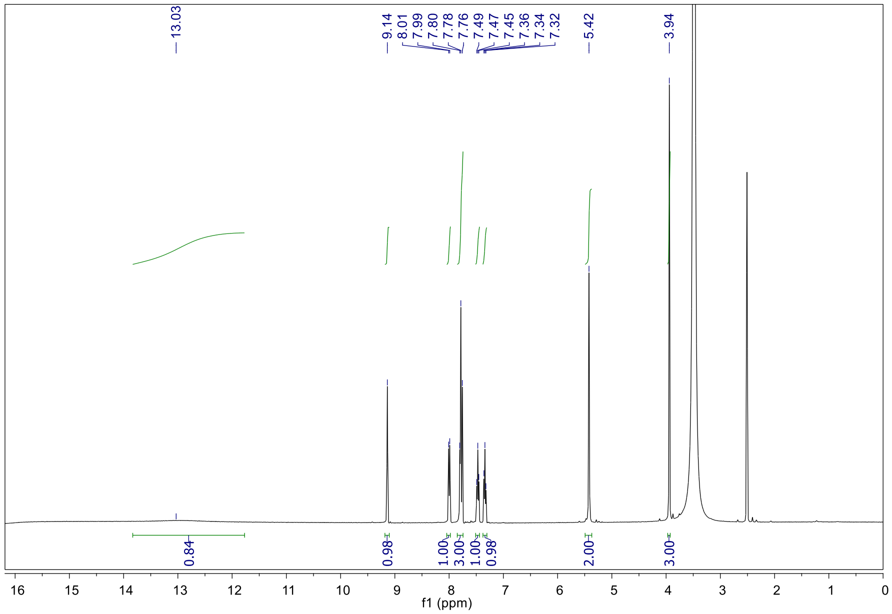

Figure S1. ^{1}H NMR of Compound **6**.

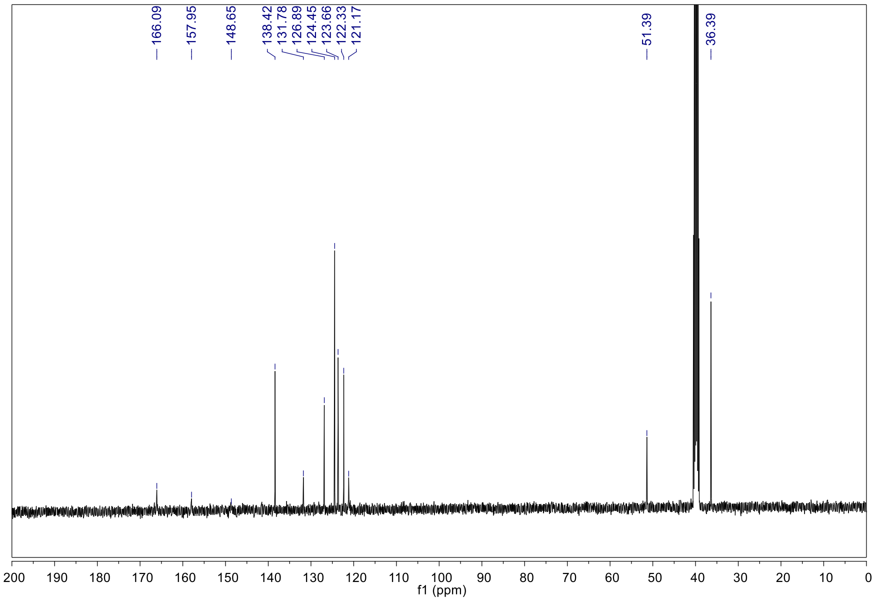

Figure S2. ^{13}C NMR of Compound **6.**

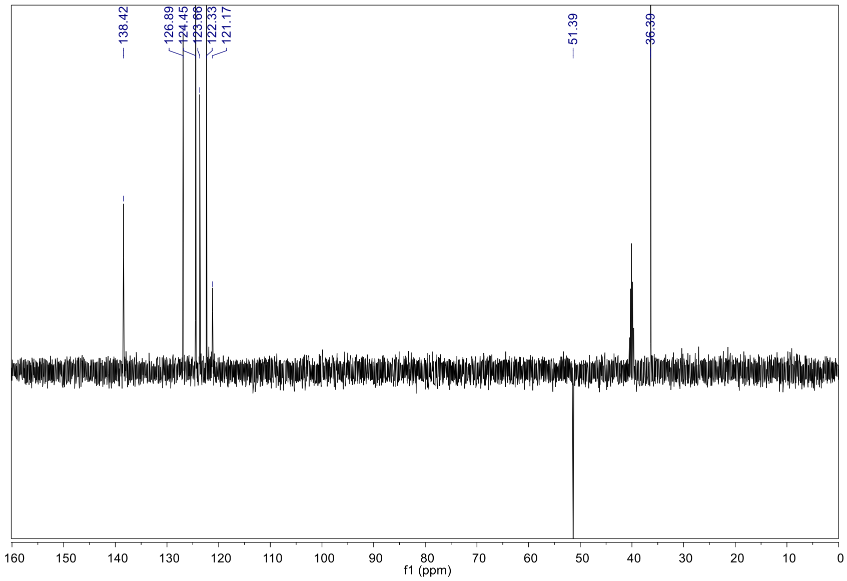

Figure S3. Dept-135 NMR of Compound **6.**

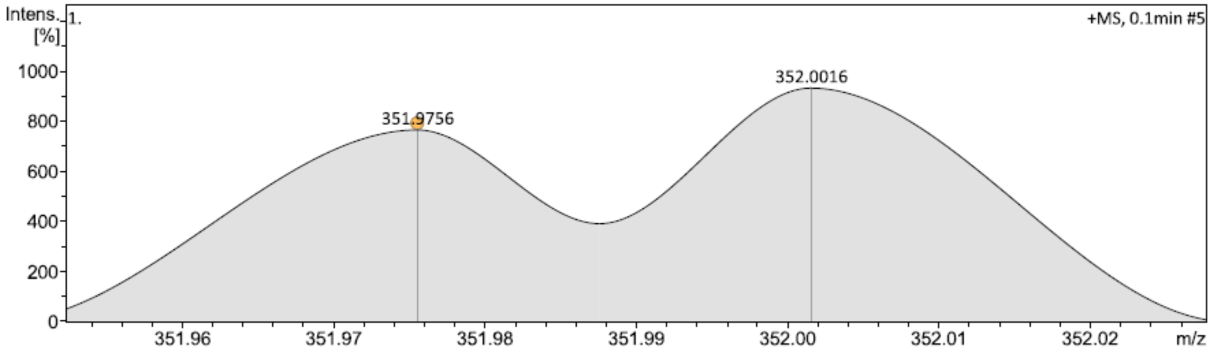

Figure S4. HRMS (ESI) of Compound **6**.

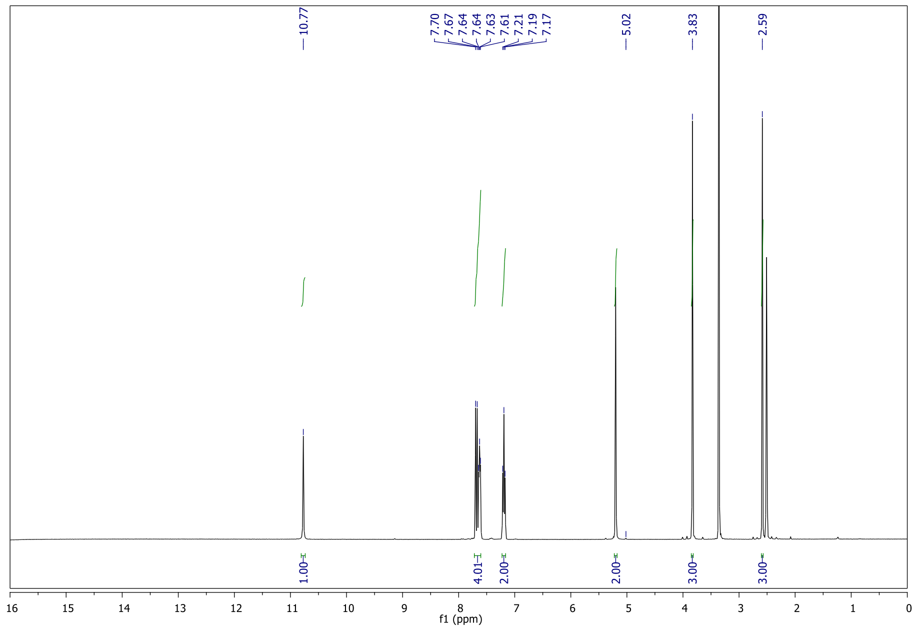

Figure S5. ^{1}H NMR of Compound **7**.

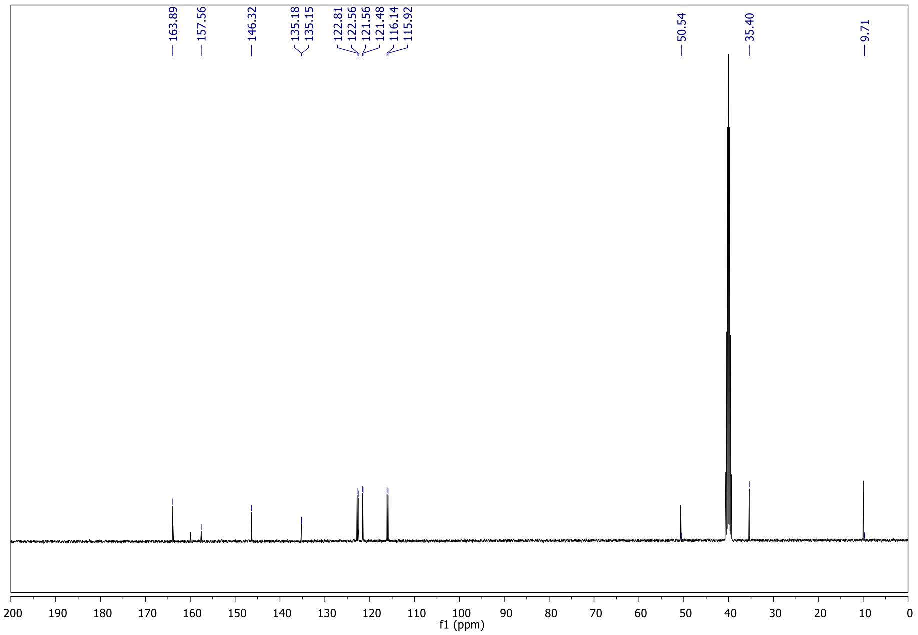

Figure S6. ^{13}C NMR of Compound **7**.

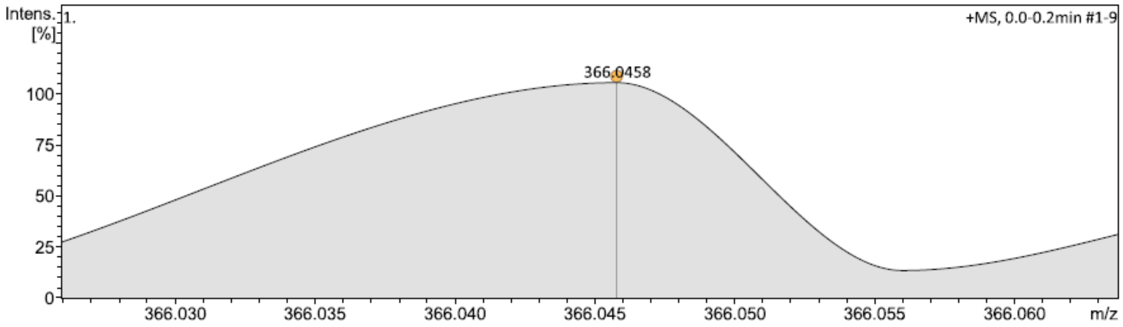

Figure S7. HRMS (ESI) of Compound **7**.

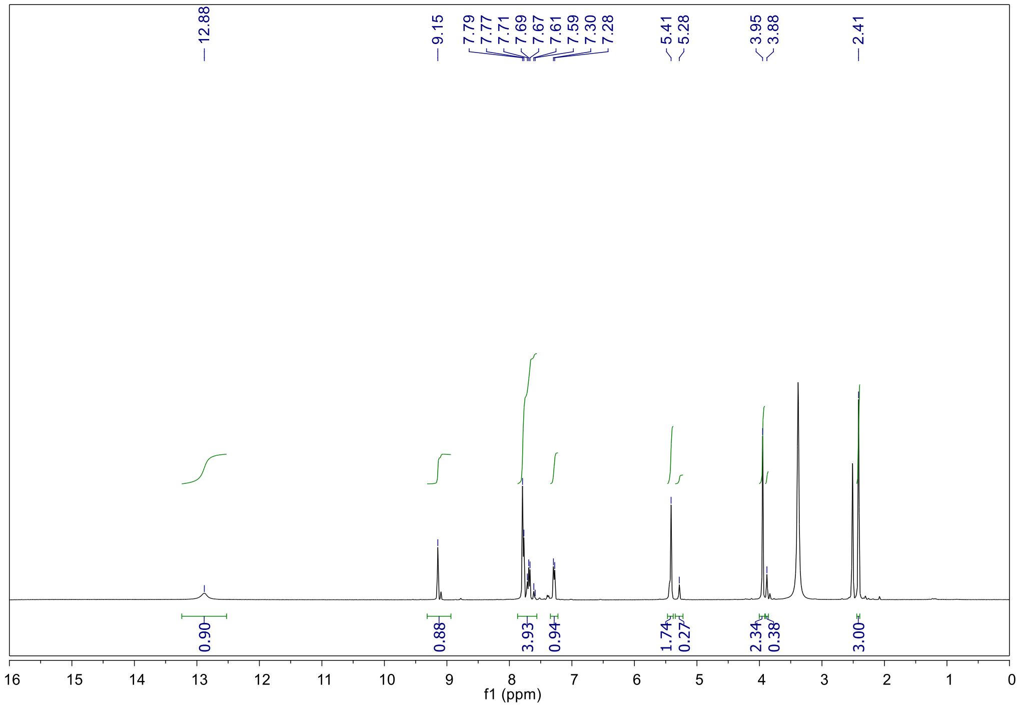

Figure S8. ^{1}H NMR of Compound **8**.

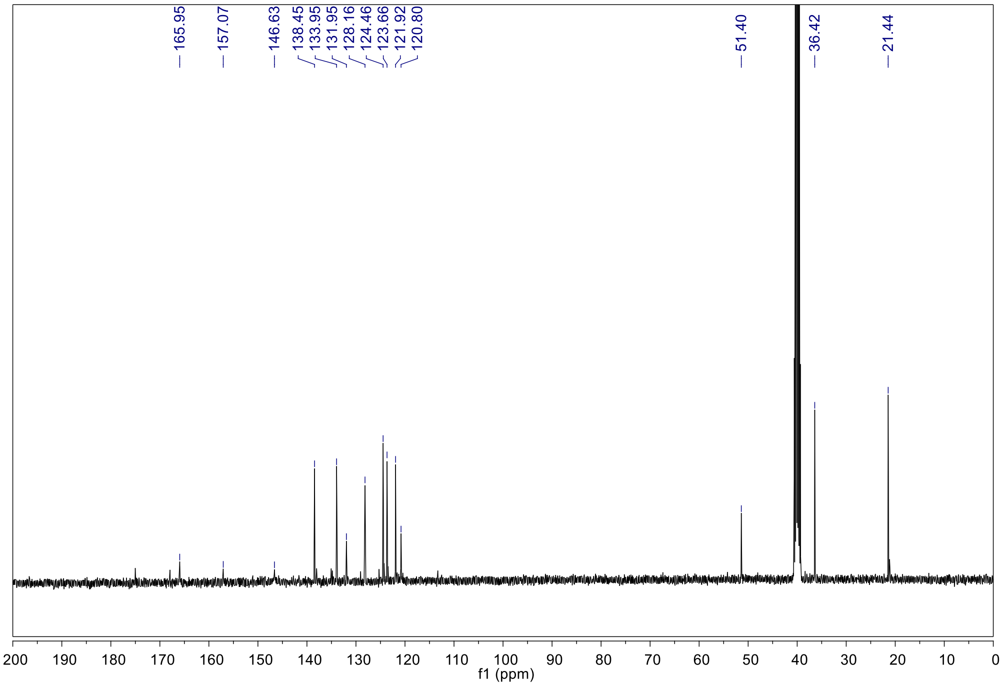

Figure S9. ^{13}C NMR of Compound **8**.

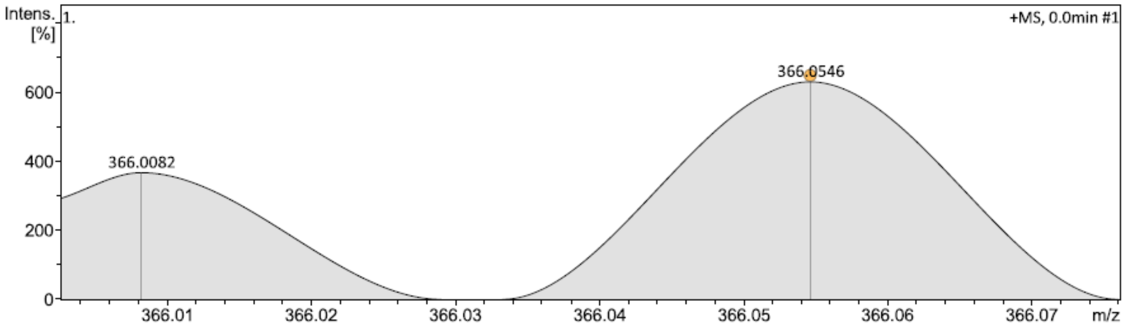

Figure S10. HRMS (ESI) of Compound **8**.

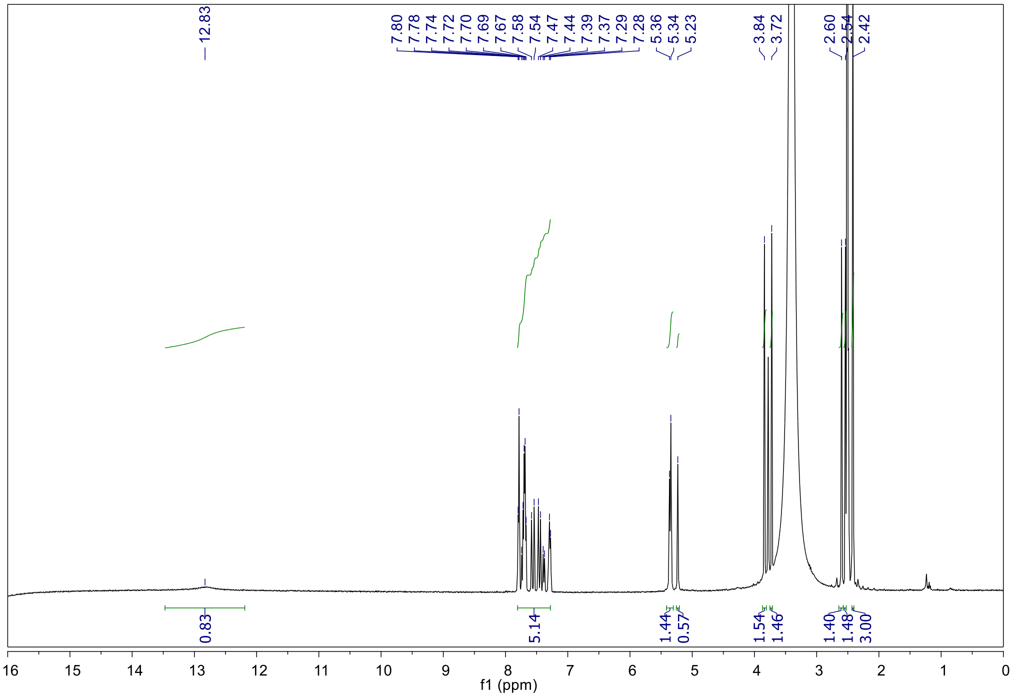

Figure S11. ^{1}H NMR of Compound **9**.

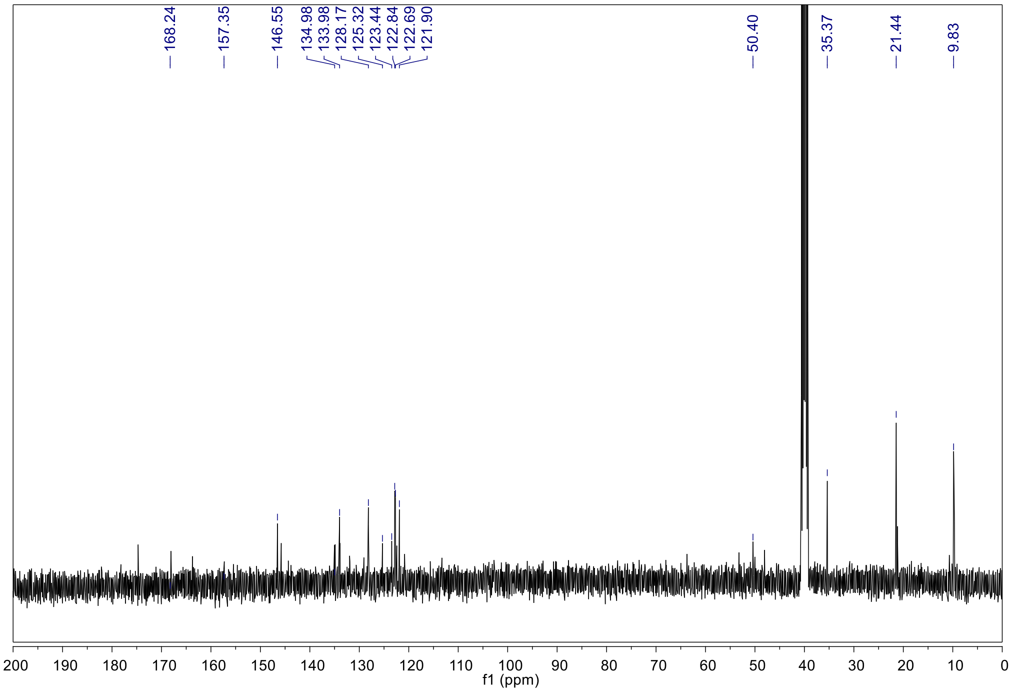

Figure S12. ^{13}C NMR of Compound **9**.

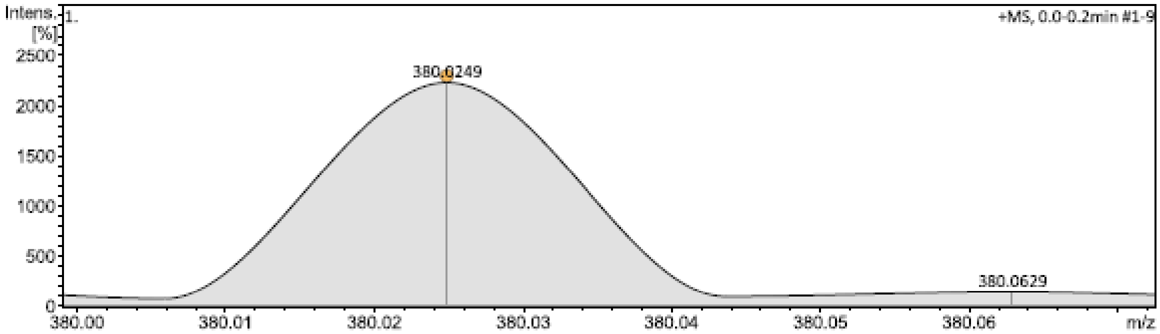

Figure S13. HRMS (ESI) of Compound **9**.

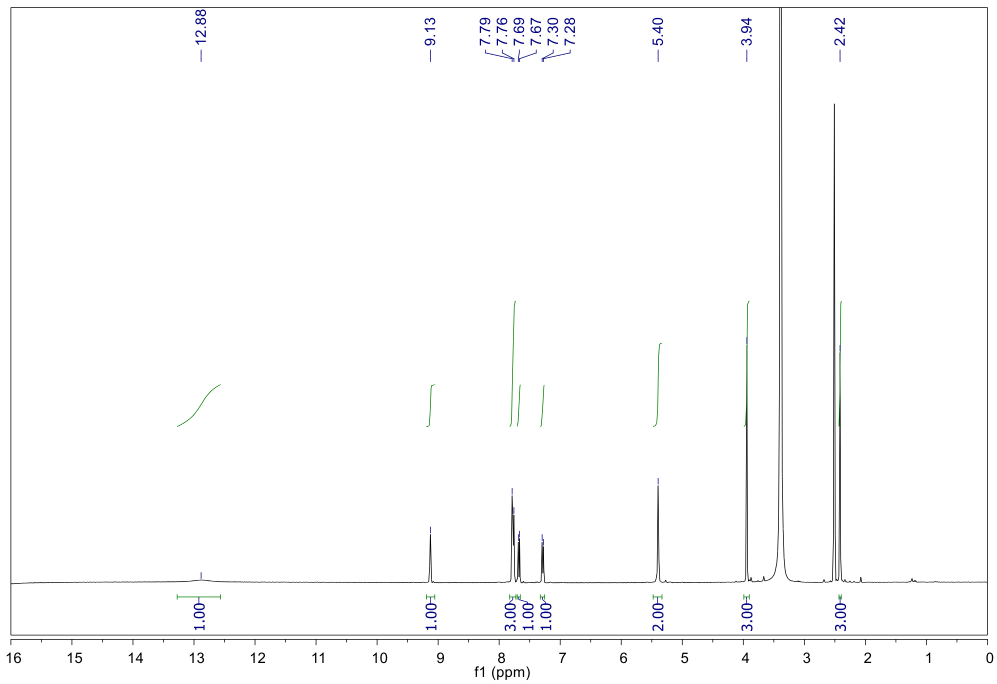

Figure S14. ^{1}H NMR of Compound **10**.

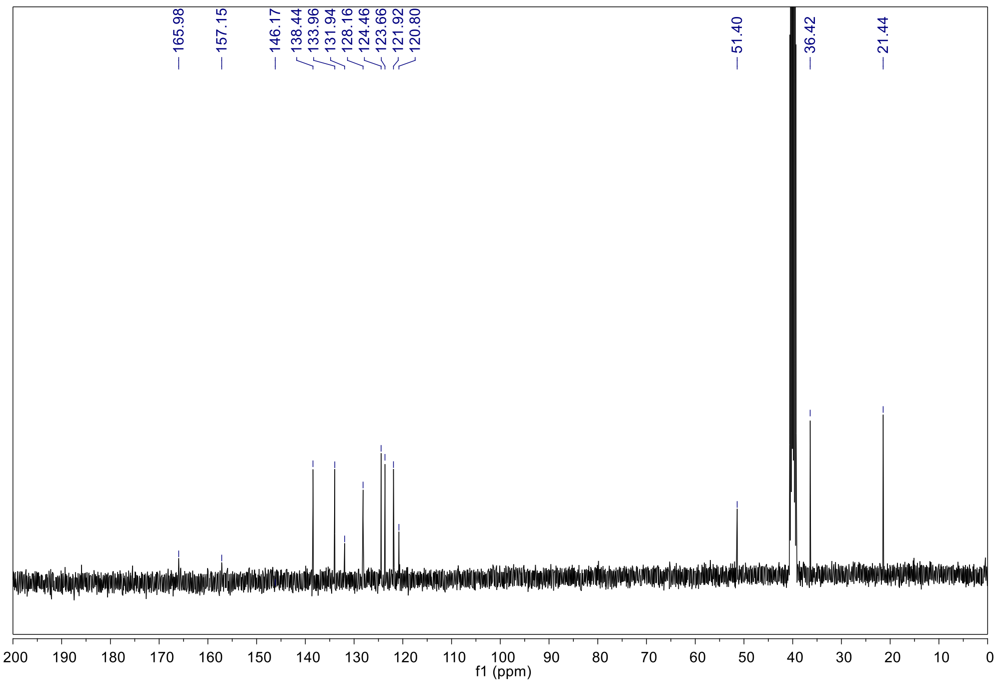

Figure S15. ^{13}C NMR of Compound **10**.

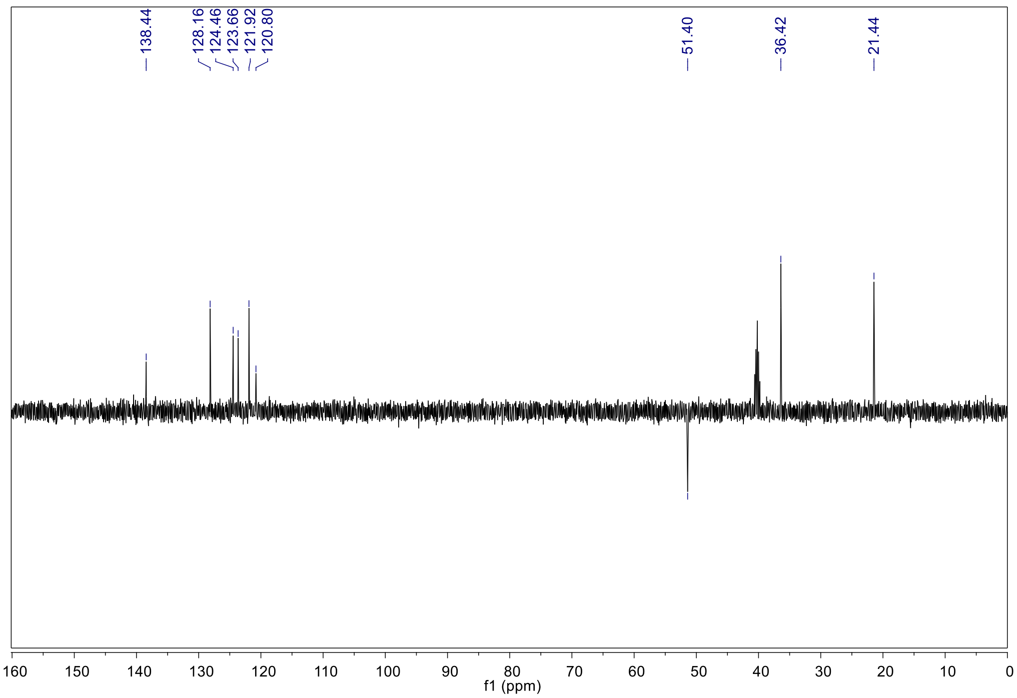

Figure S16. Dept-135 NMR of Compound **10.**

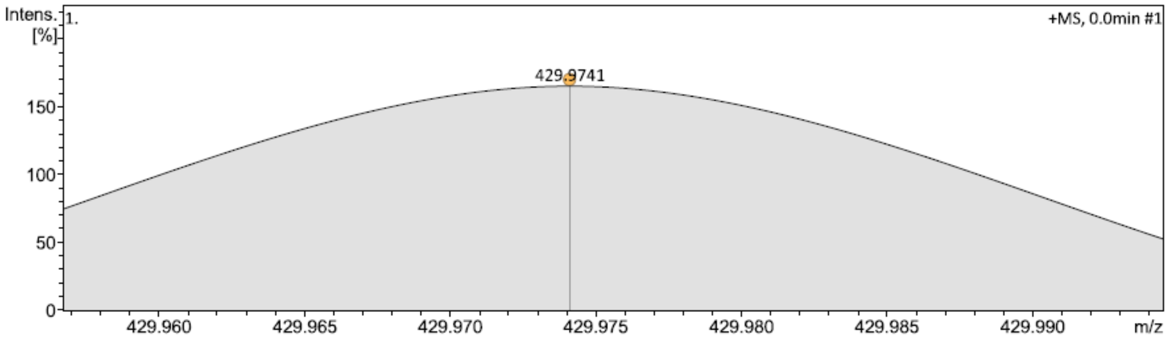

Figure S17. HRMS (ESI) of Compound **10**.

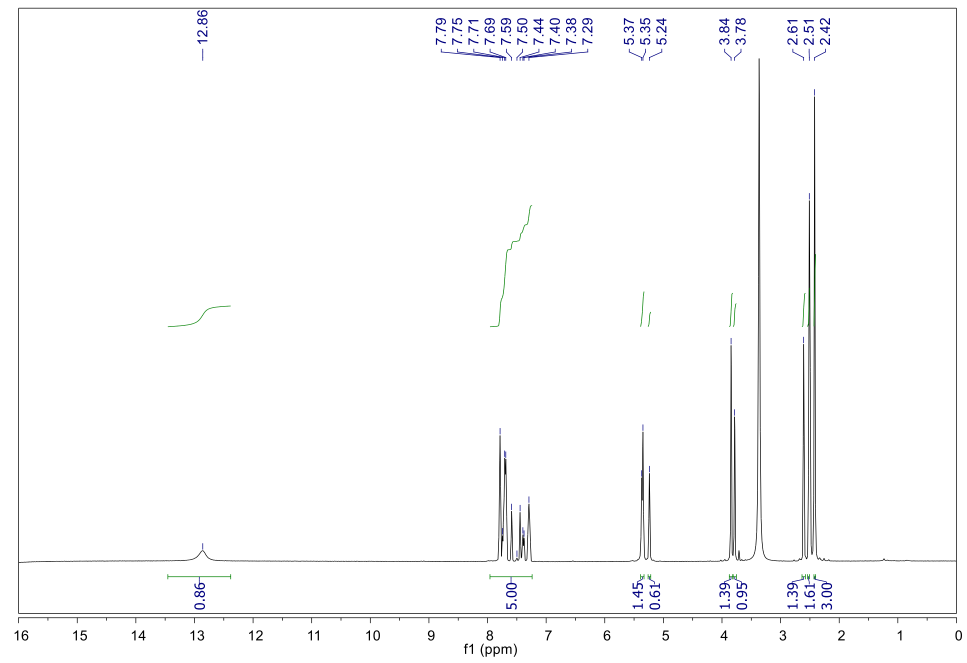

Figure S18. ^{1}H NMR of Compound **11**.

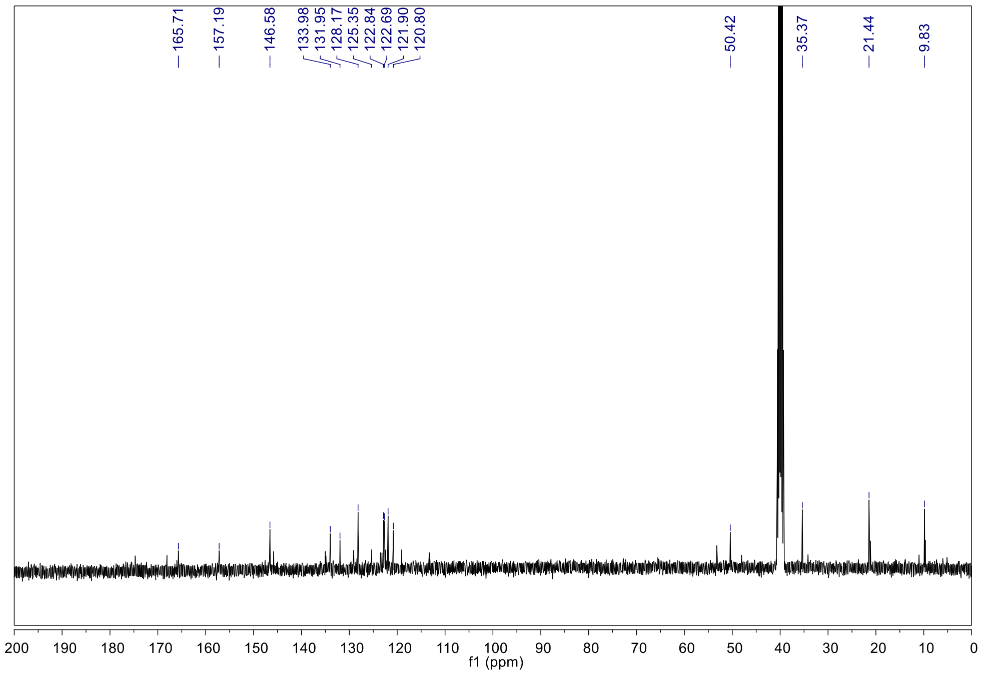

Figure S19. ^{13}C NMR of Compound **11**.

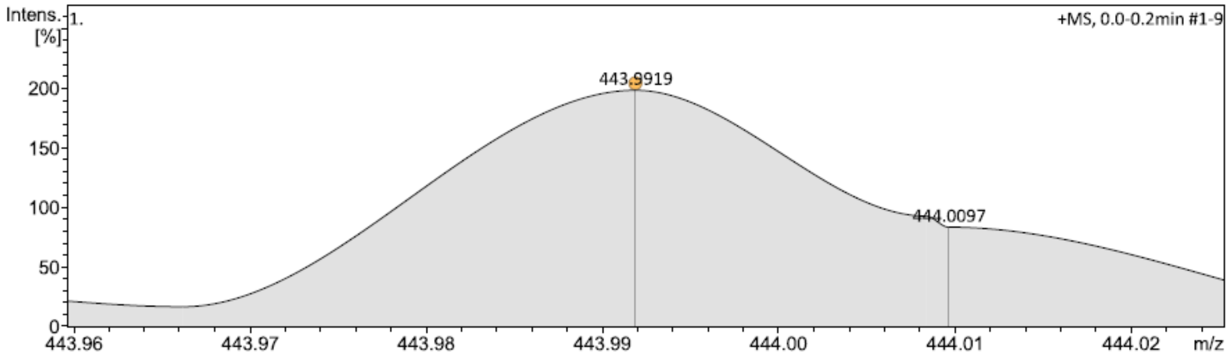

Figure S20. HRMS (ESI) of Compound **11**.

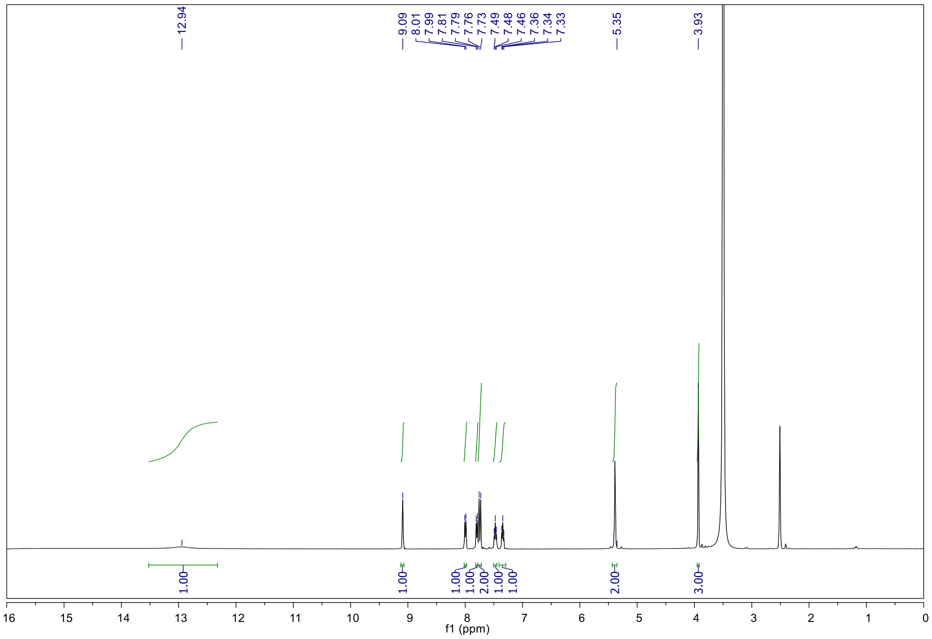

Figure S21. ^{1}H NMR of Compound **12**.

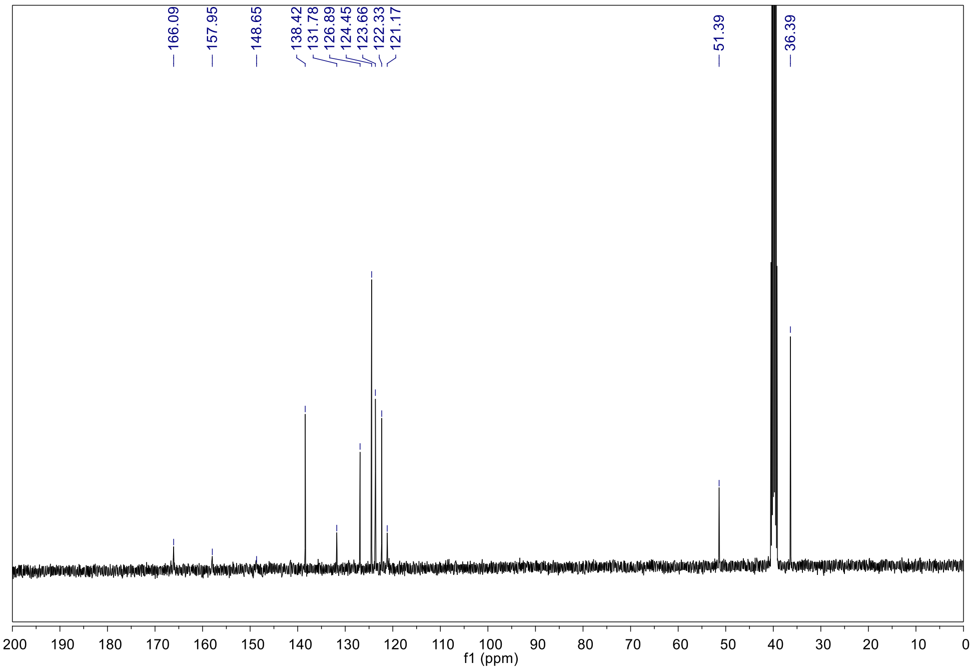

Figure S22. ^{13}C NMR of Compound **12.**

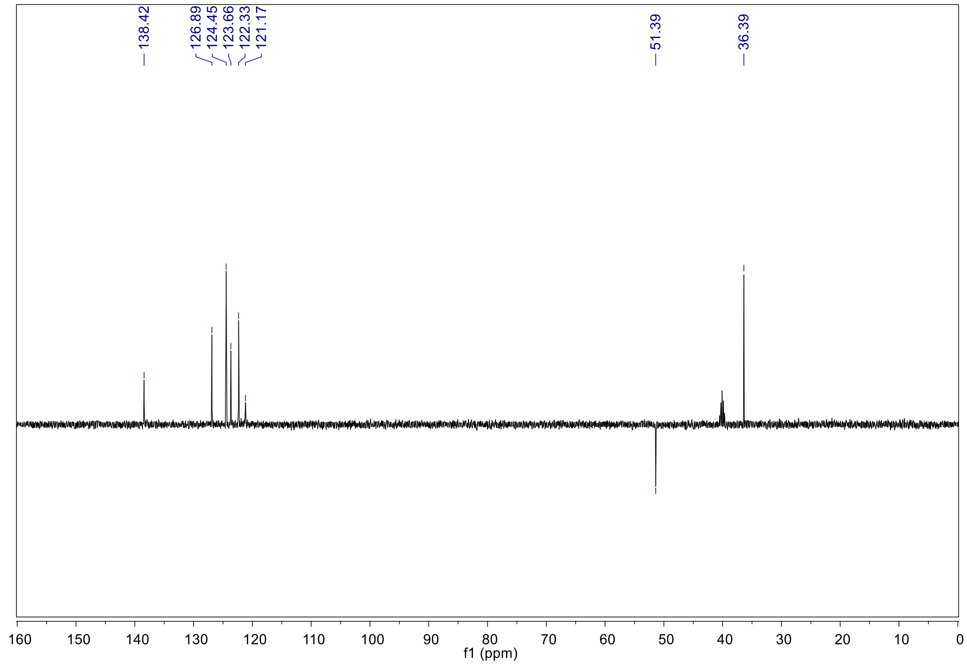

Figure S23. Dept-135 NMR of Compound **12.**

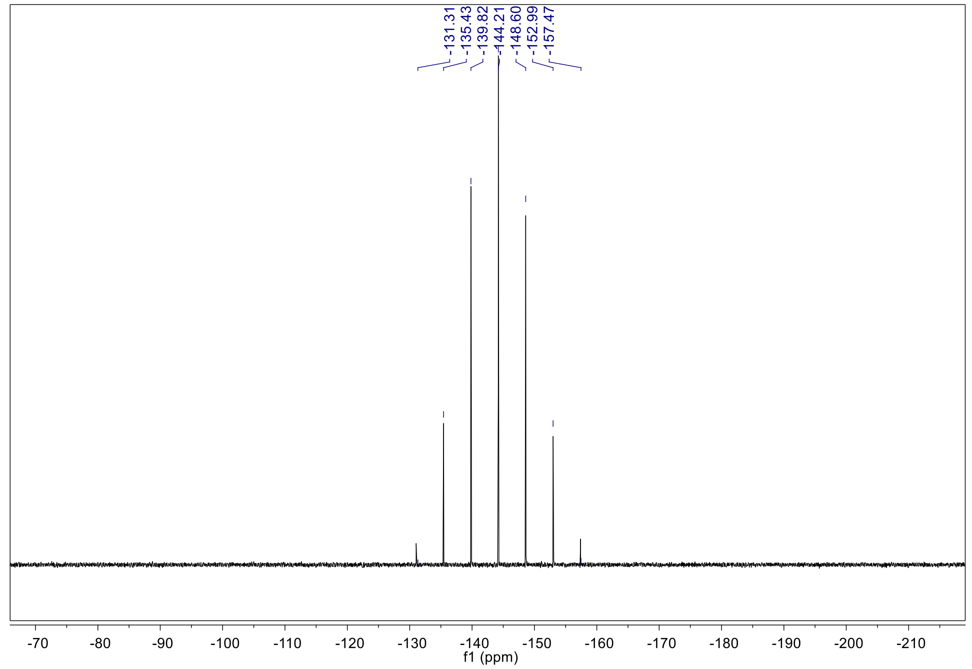

Figure S24. ^{31}PNMR of Compound **12**.

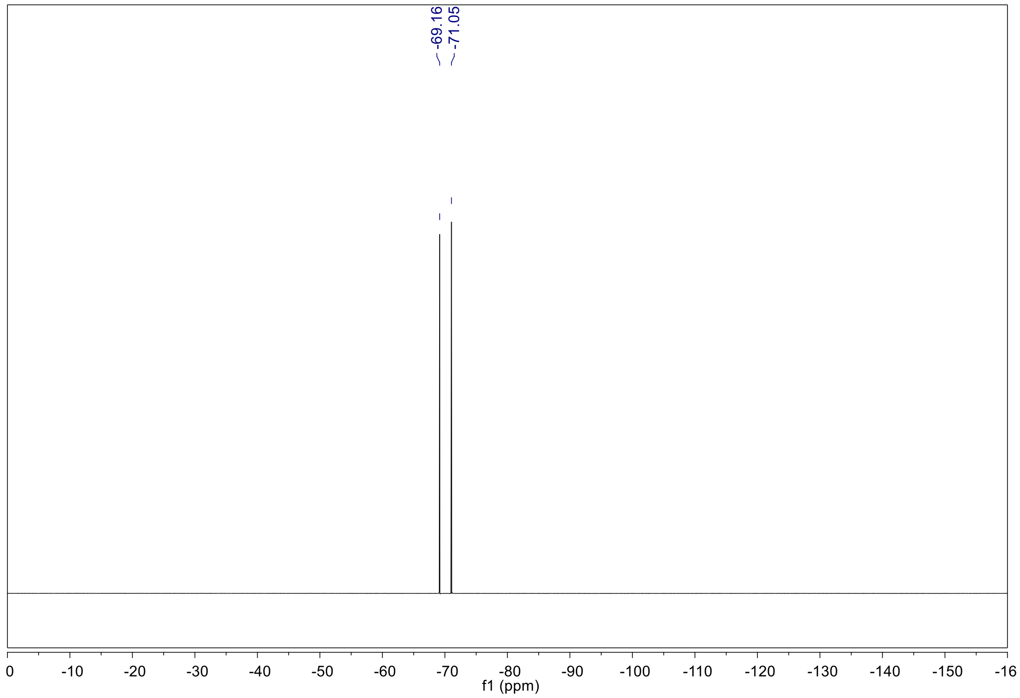

Figure S25. ^{19}FNMR of Compound **12**.

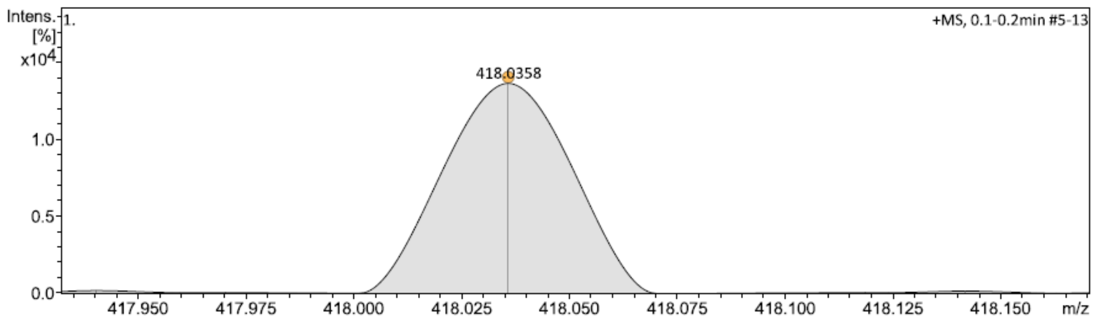

Figure S26. HRMS (ESI) of Compound **12**.

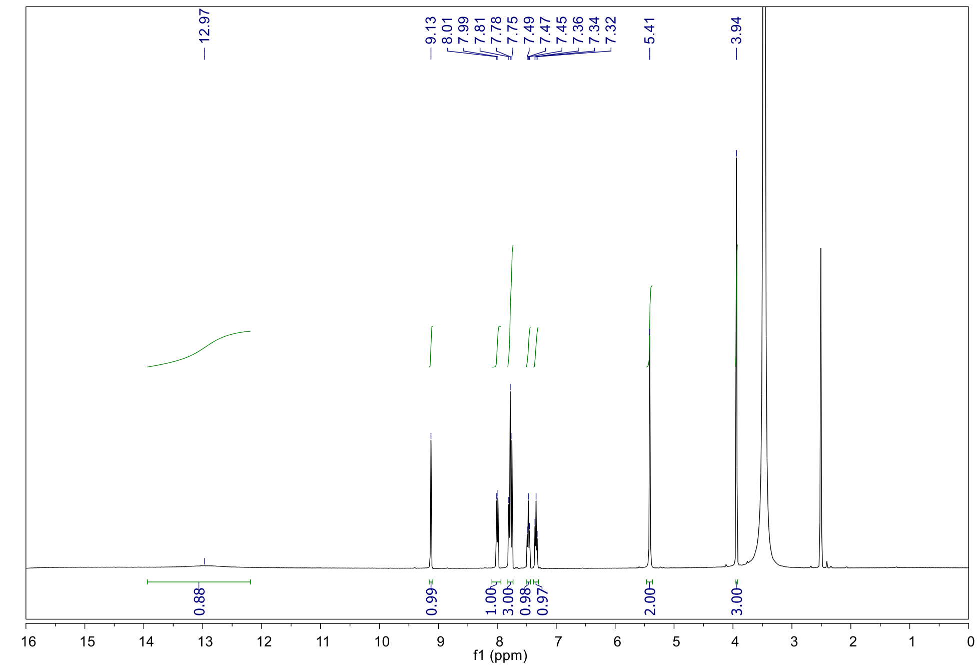

Figure S27. ^{1}H NMR of Compound **13**.

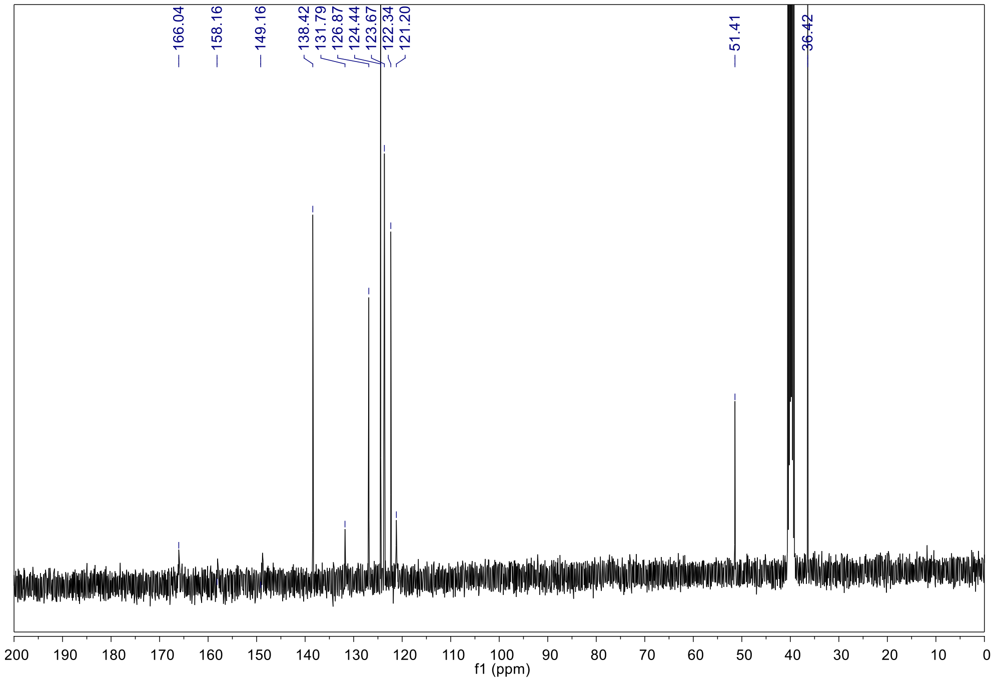

Figure S28. ^{13}C NMR of Compound **13**.

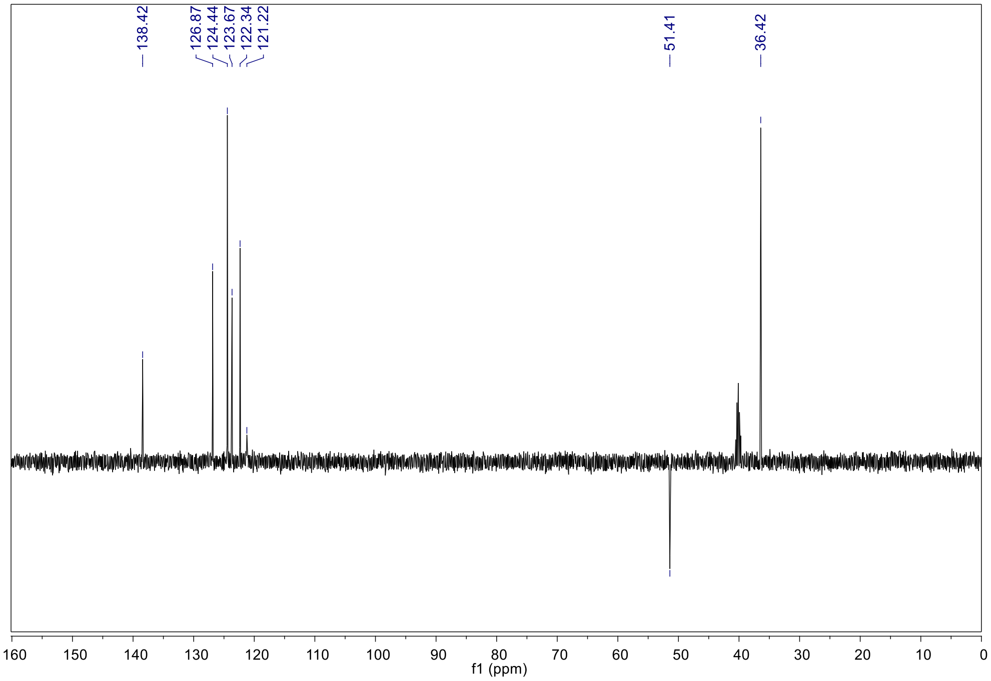

Figure S29. Dept-135 NMR of Compound **13.**

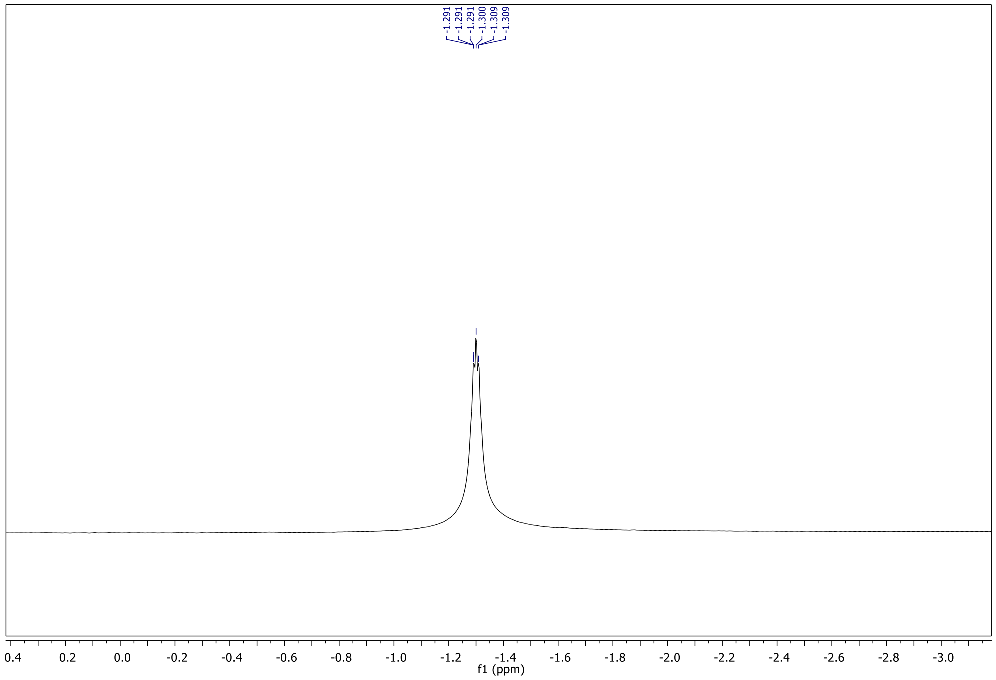

Figure S30. ^{11}B NMR of Compound **13**.

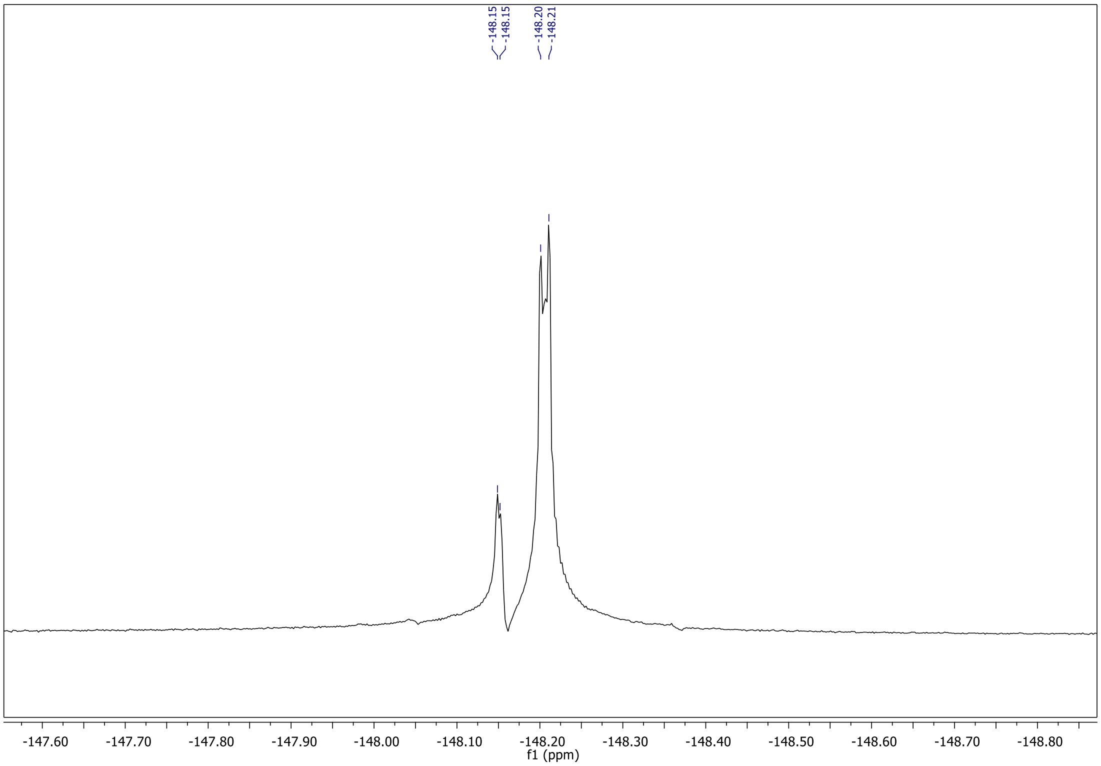

Figure S31. ^{19}FNMR of Compound **13**.

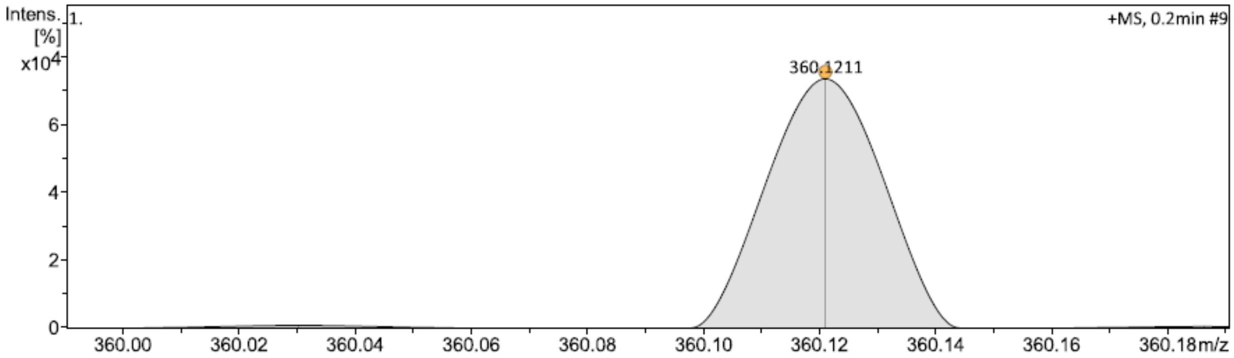

Figure S32. HRMS (ESI) of Compound **13**.

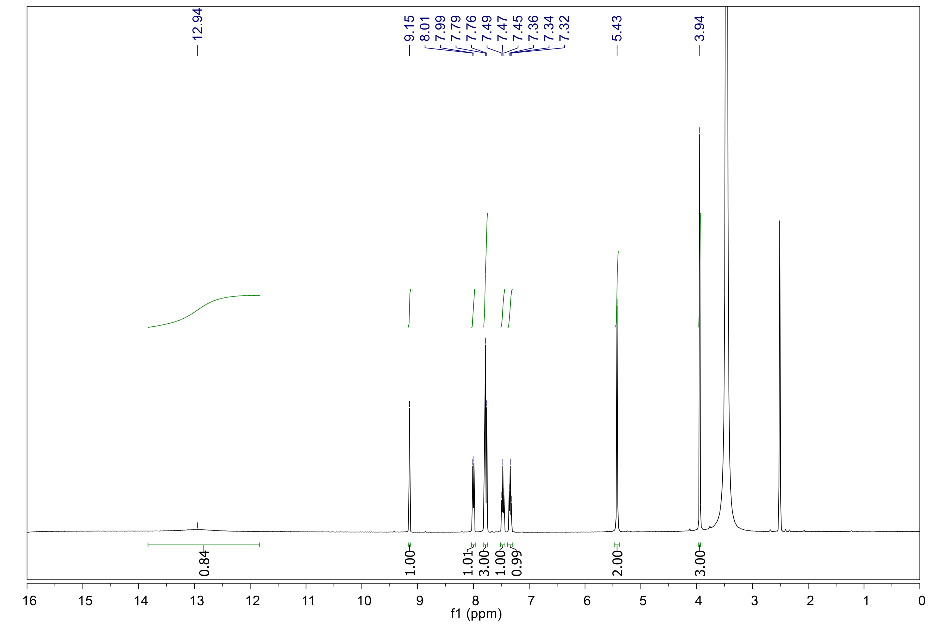

Figure S33. ^{1}H NMR of Compound **14**.

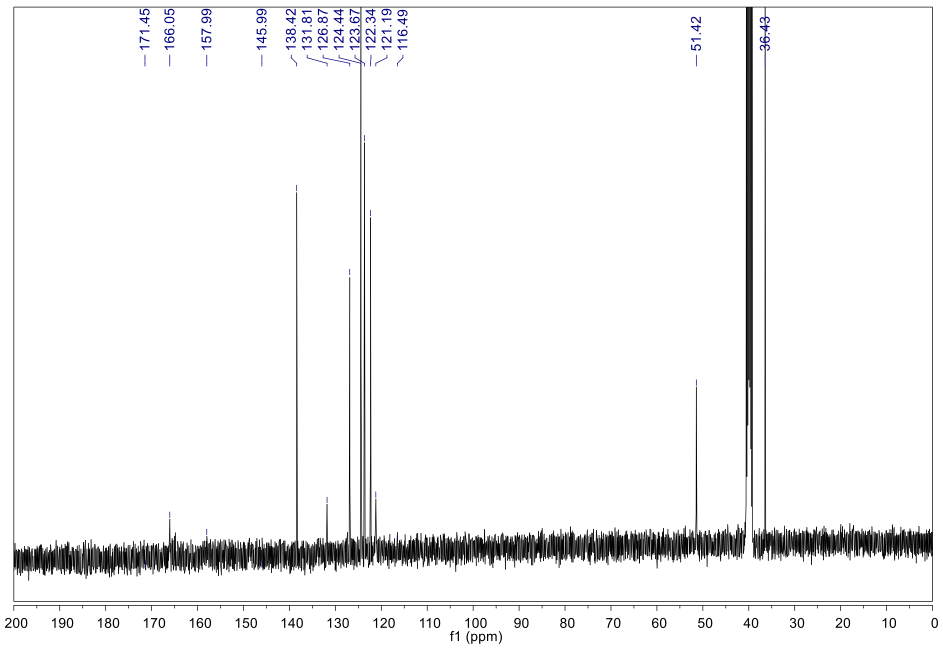

Figure S34. ^{13}C NMR of Compound **14**.

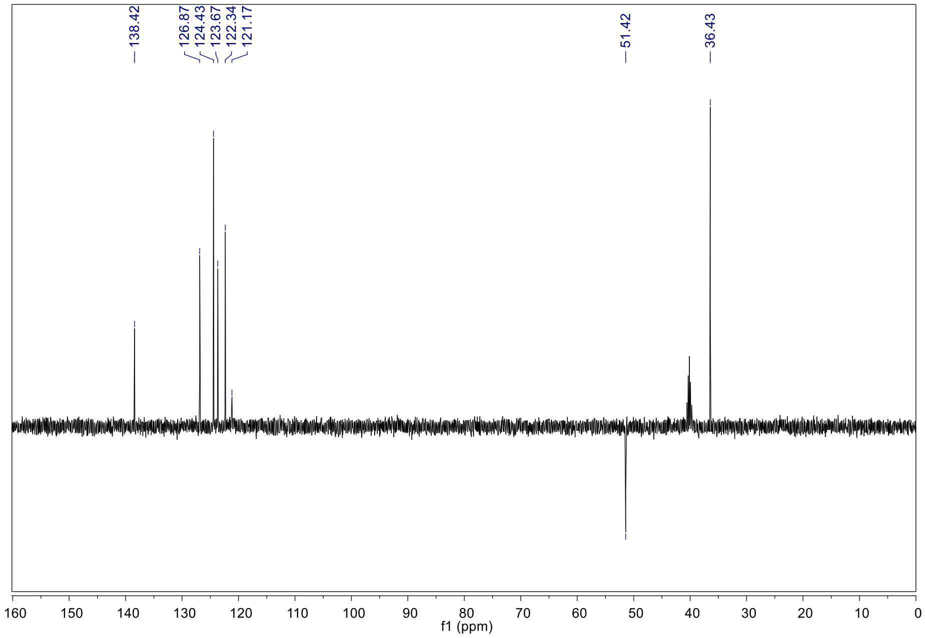

Figure S35. Dept-135 NMR of Compound **14**.

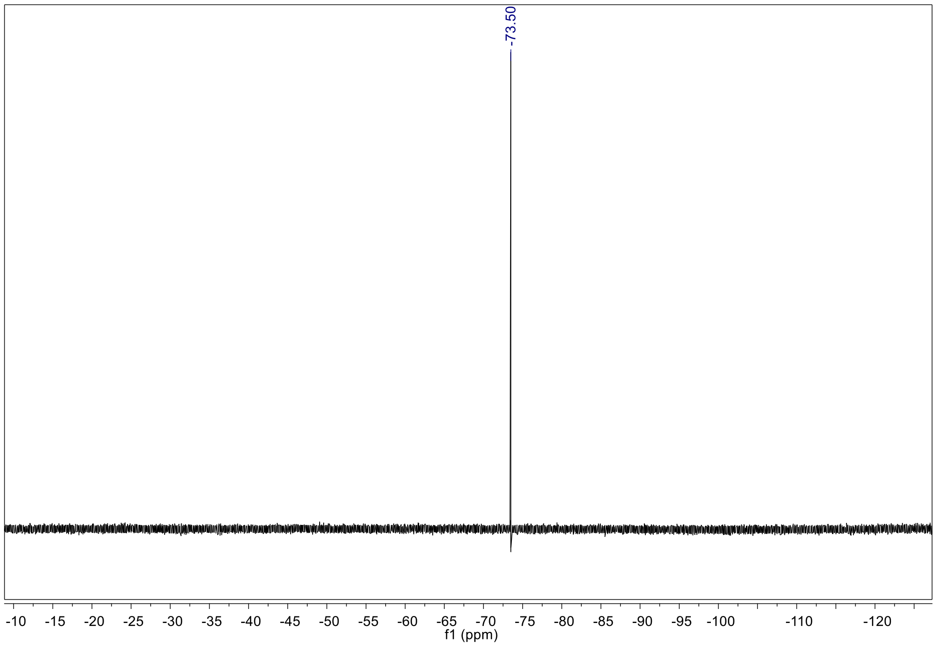

Figure S36. ^{19}FNMR of Compound **14**.

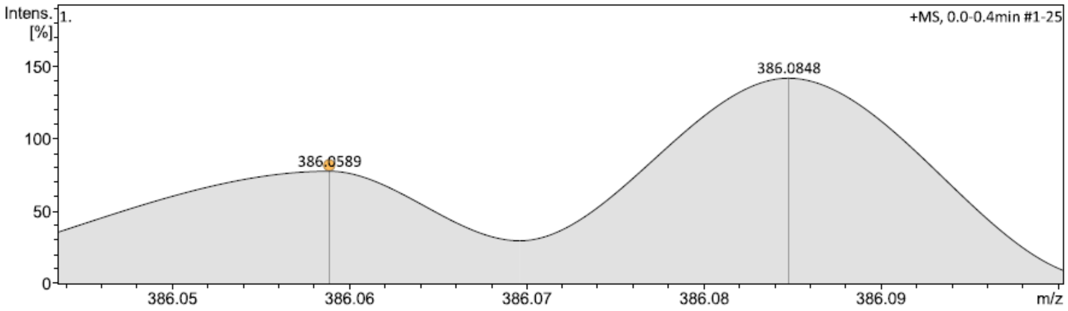

Figure S37. HRMS (ESI) of Compound **14**.

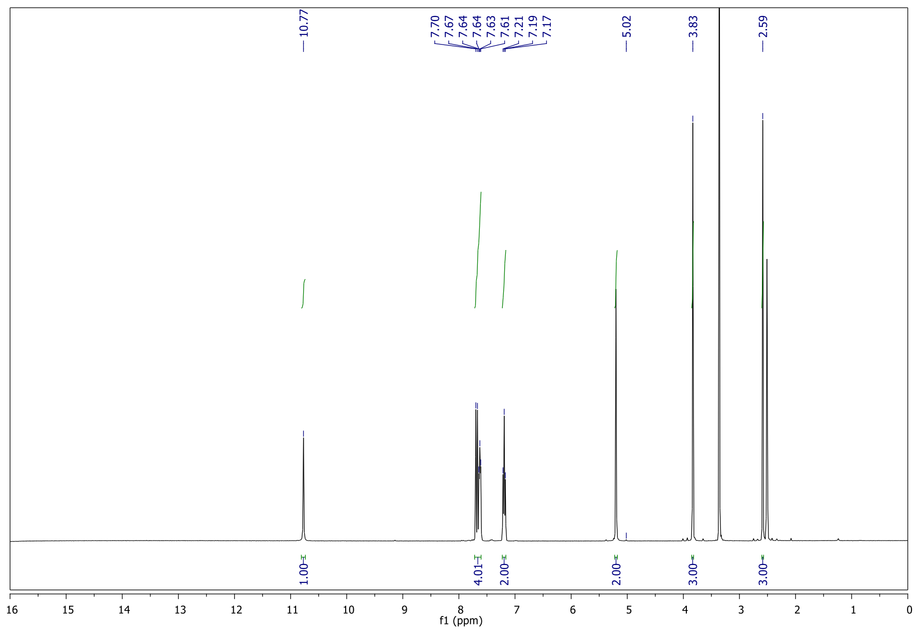

Figure S38. ^{1}H NMR of Compound **15**.

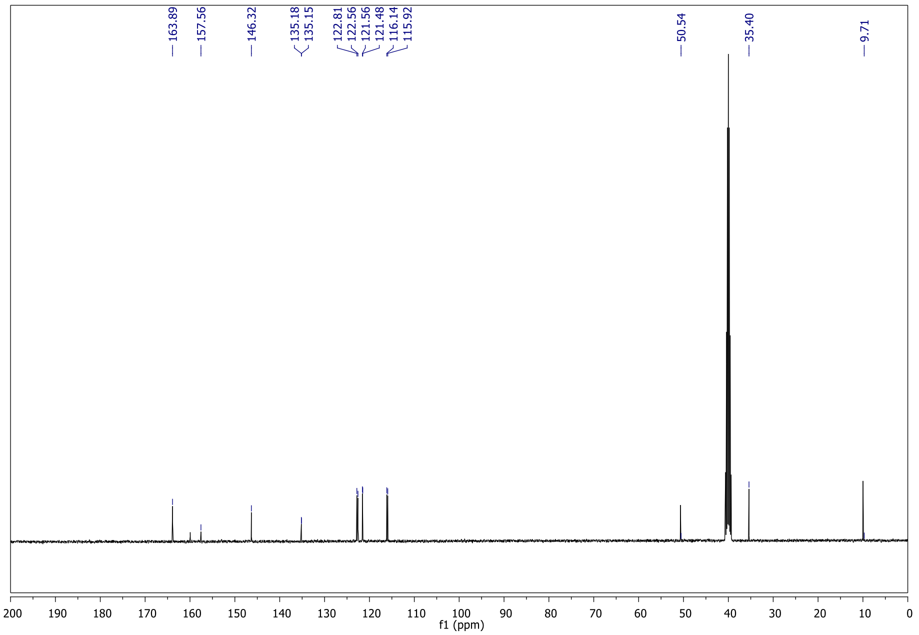

Figure S39. ^{13}C NMR of Compound **15**.

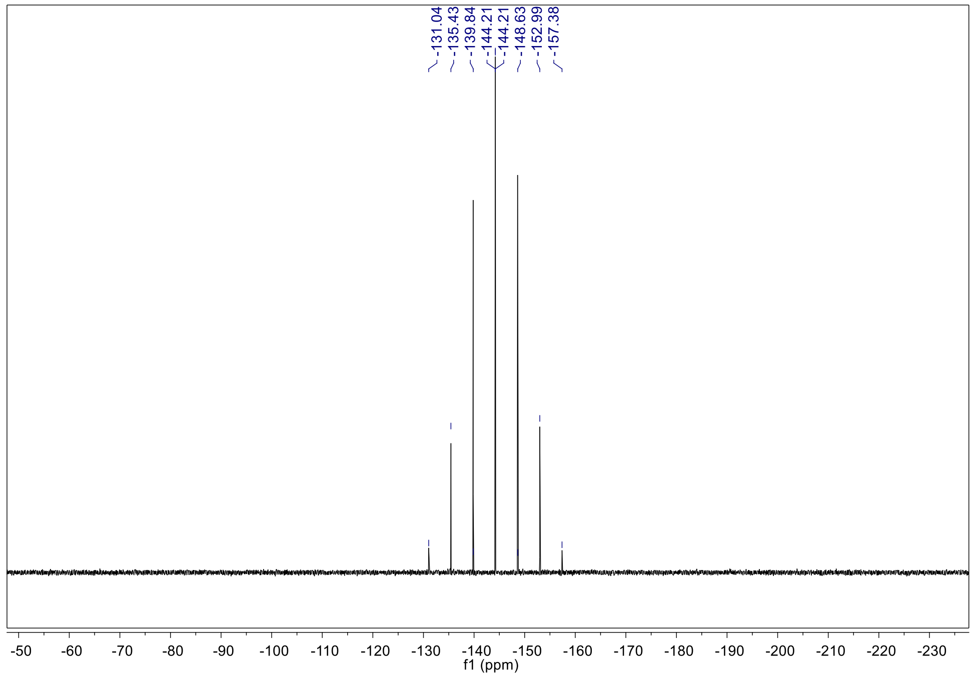

Figure S40. ^{31}PNMR of Compound **15**.

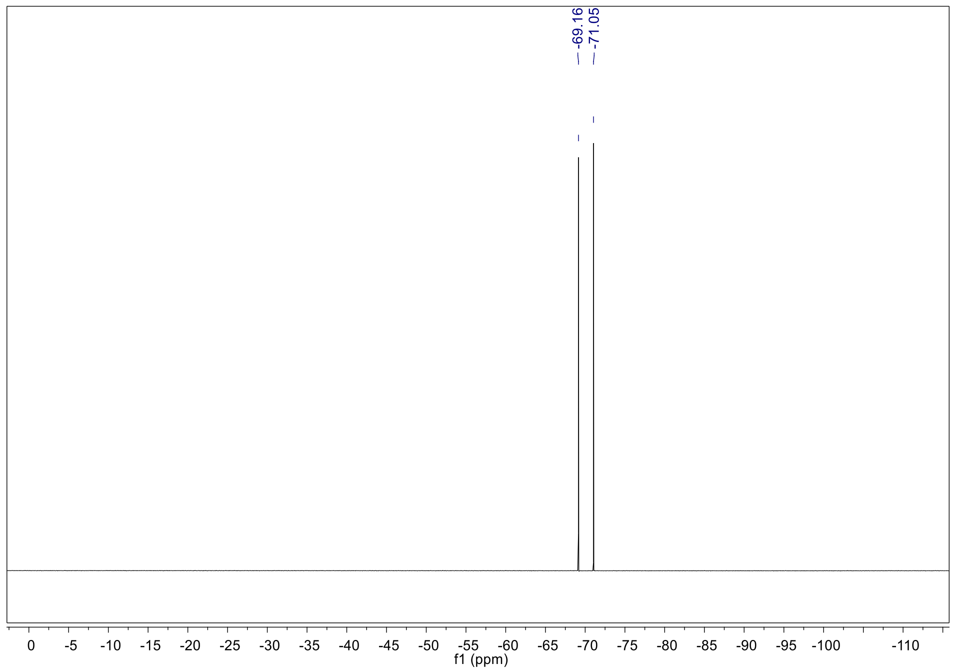

Figure S41. ^{19}FNMR of Compound **15**.

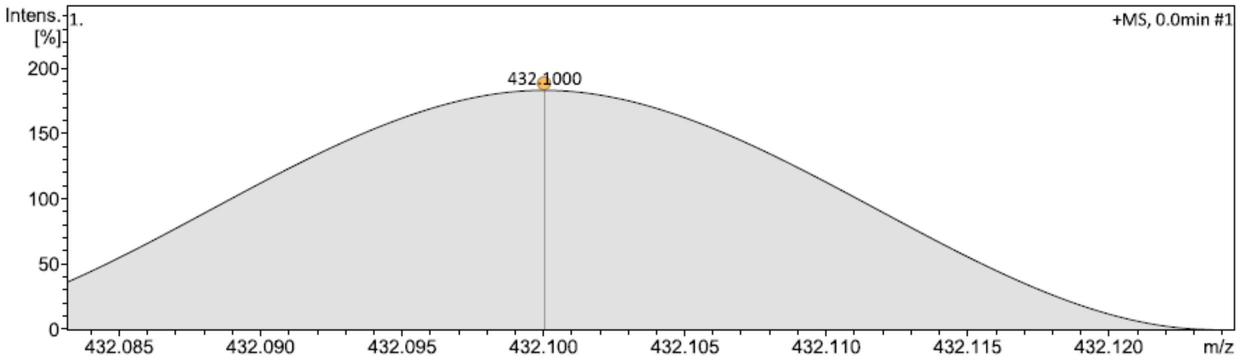

Figure S42. HRMS (ESI) of Compound **15**.

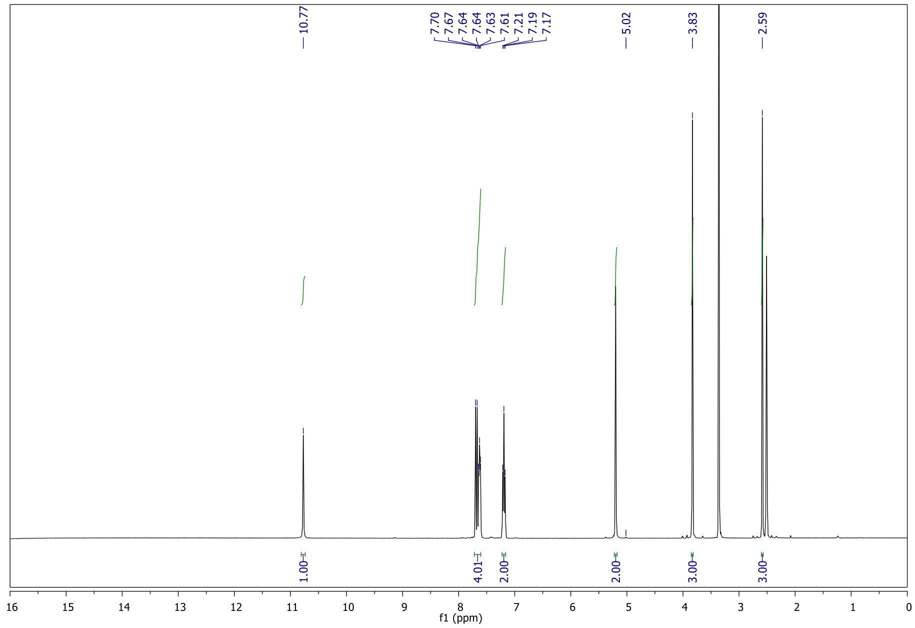

Figure S43. ^{1}H NMR of Compound **16**.

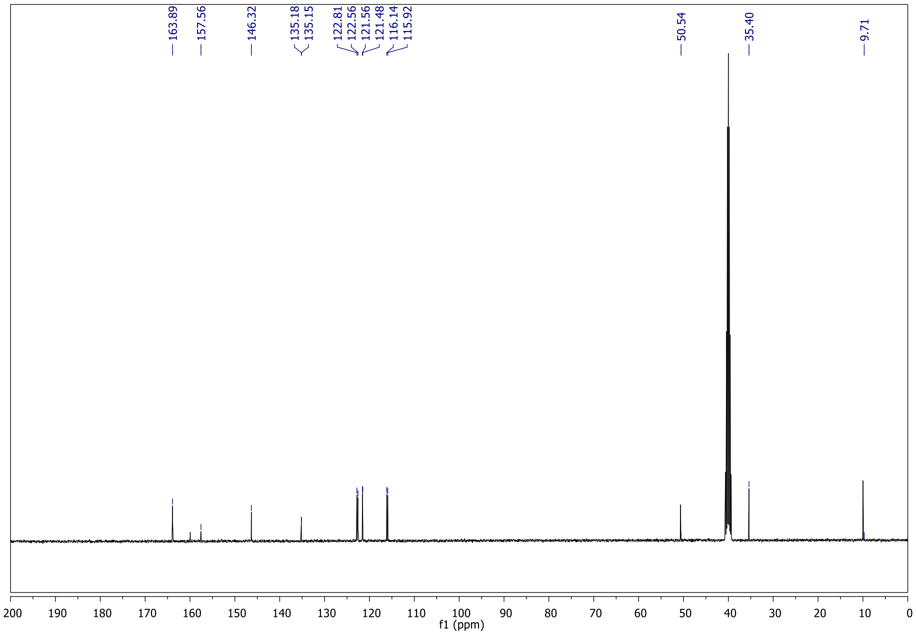

Figure S44. ^{13}C NMR of Compound **16**.

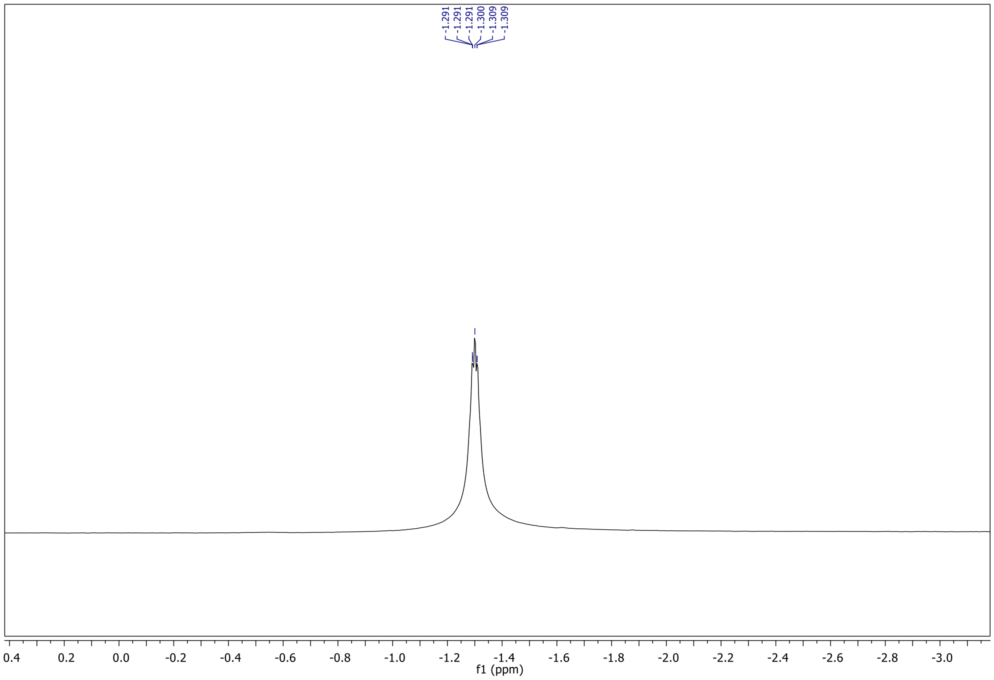

Figure S45. ^{11}B NMR of Compound **16**.

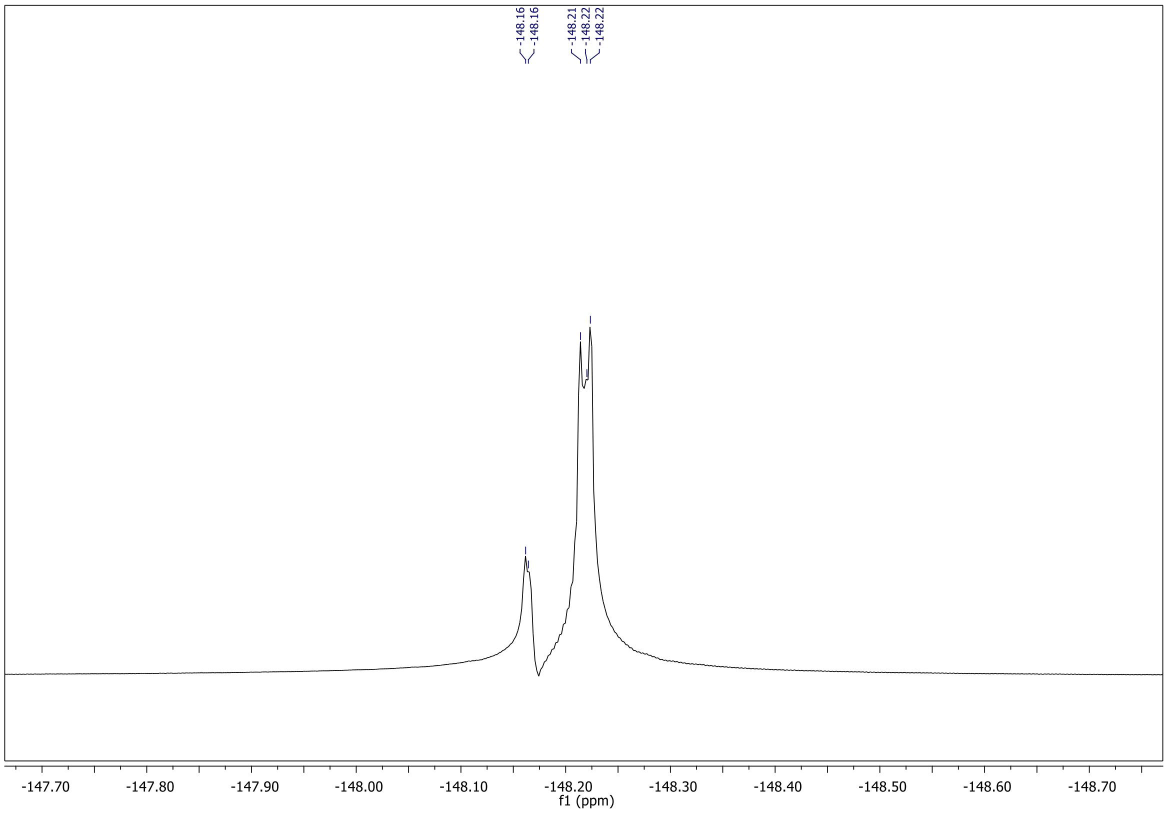

Figure S46. ^{19}FNMR of Compound **16**.

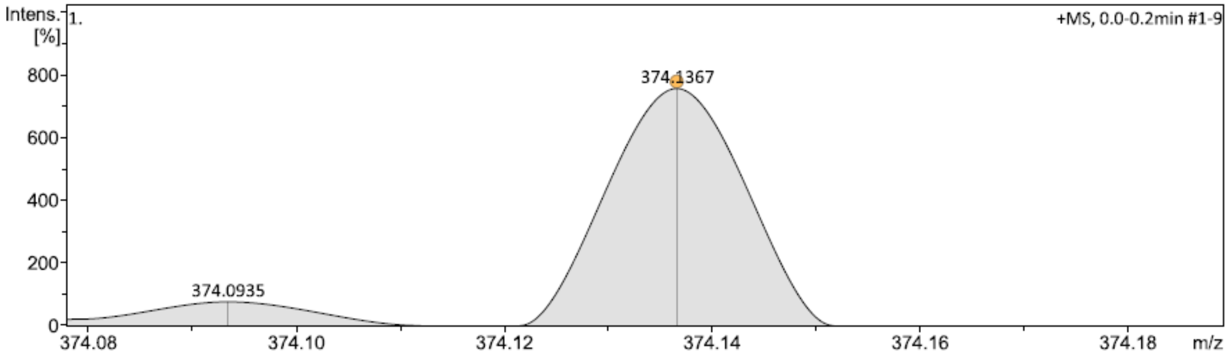

Figure S47. HRMS (ESI) of Compound **16**.

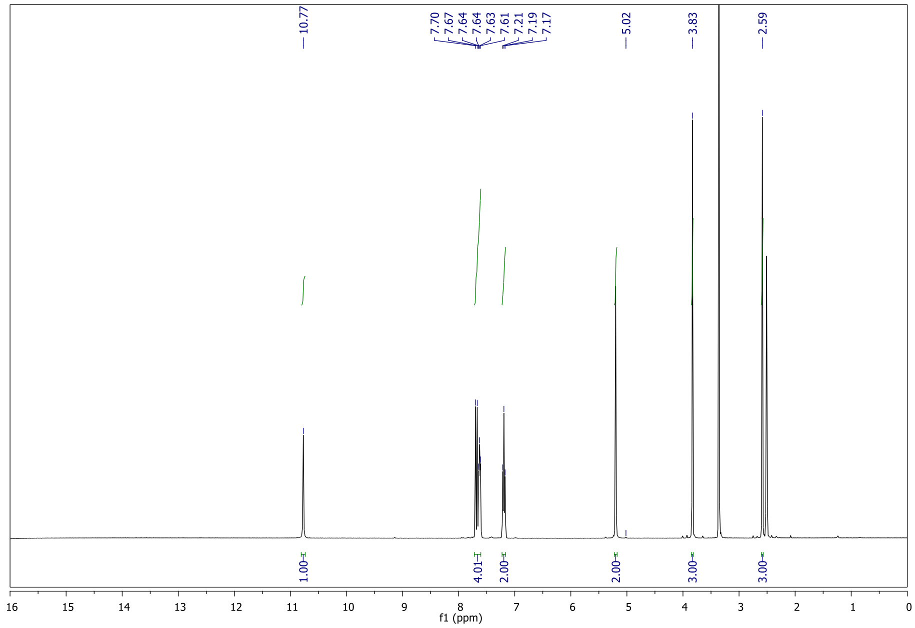

Figure S48. ^{1}H NMR of Compound **17**.

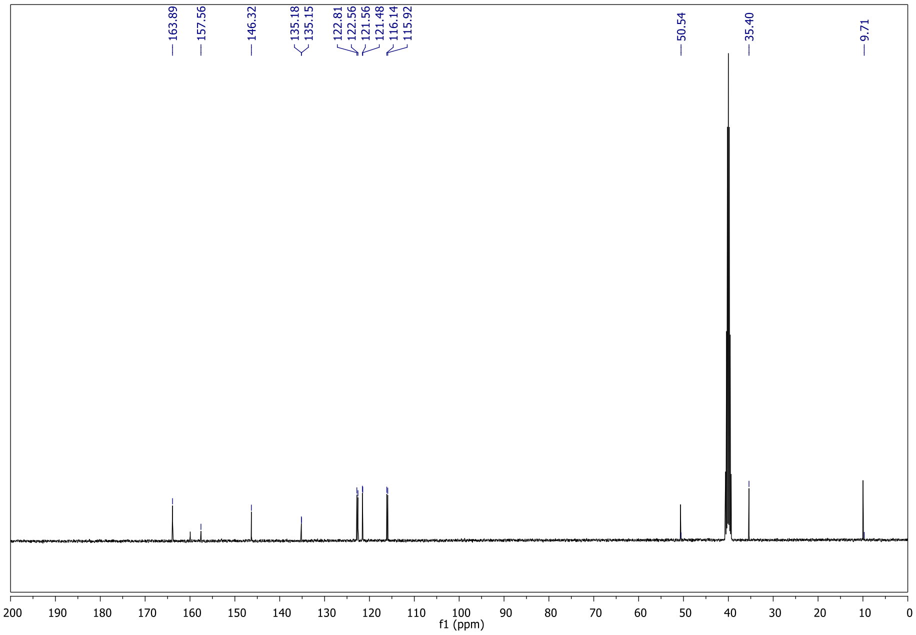

Figure S49. ^{13}C NMR of Compound **17**.

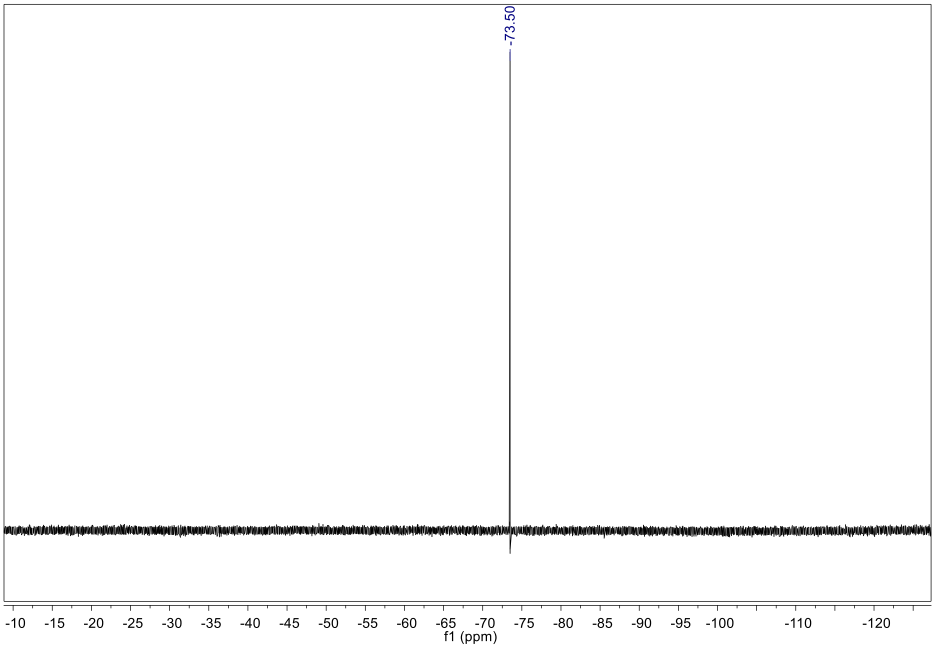

Figure S50. ^{19}FNMR of Compound **17**.

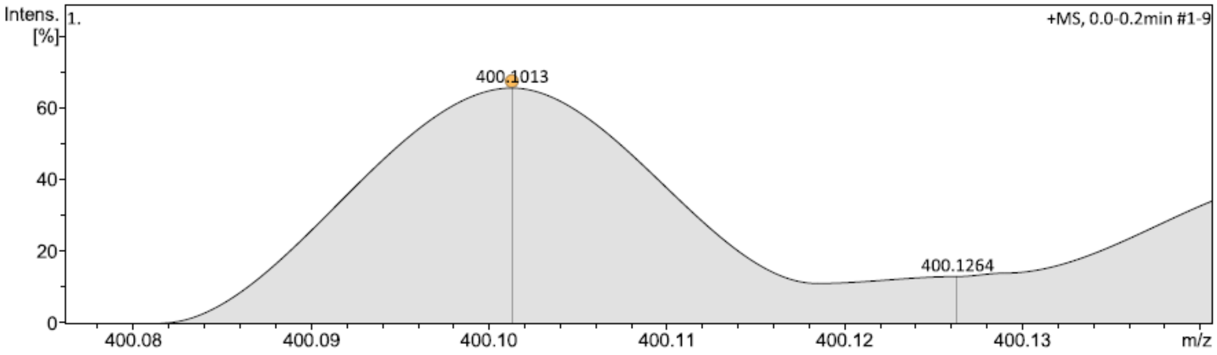

Figure S51. HRMS (ESI) of Compound **17**.

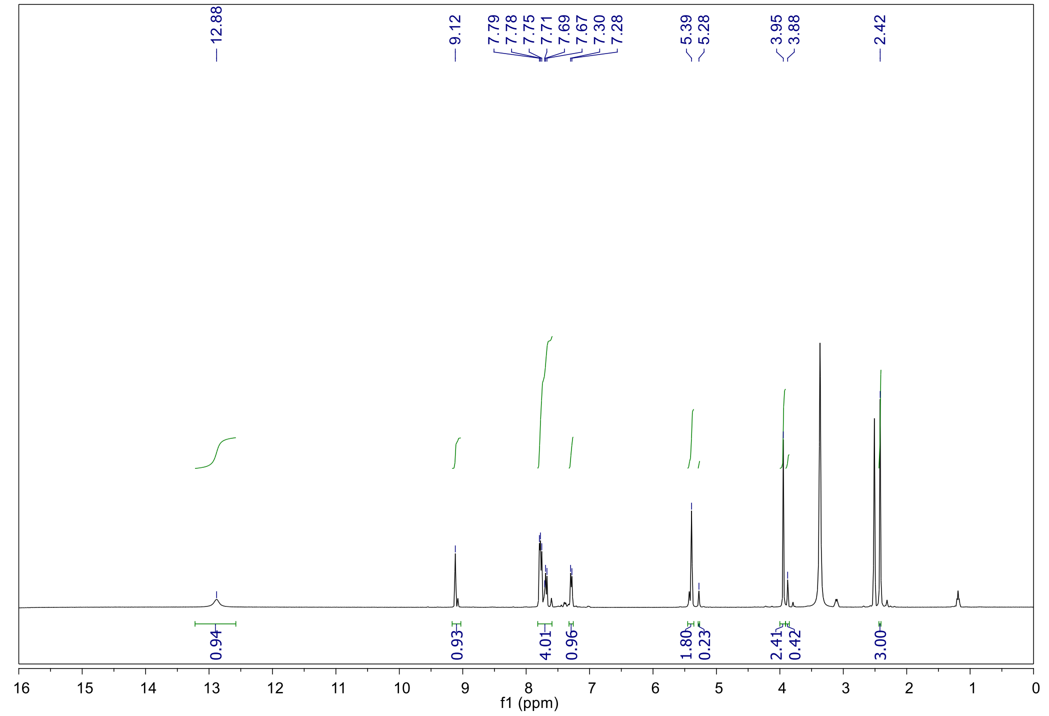

Figure S52. ¹H NMR of Compound **18**.

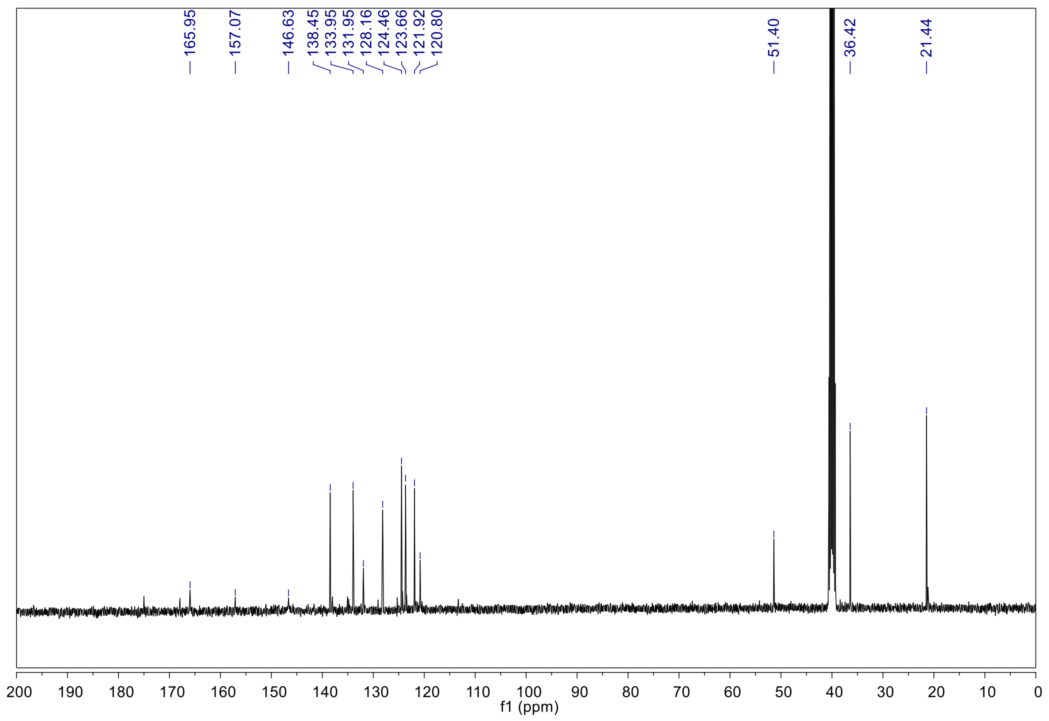

Figure S53. ^{13}C NMR of Compound **18**.

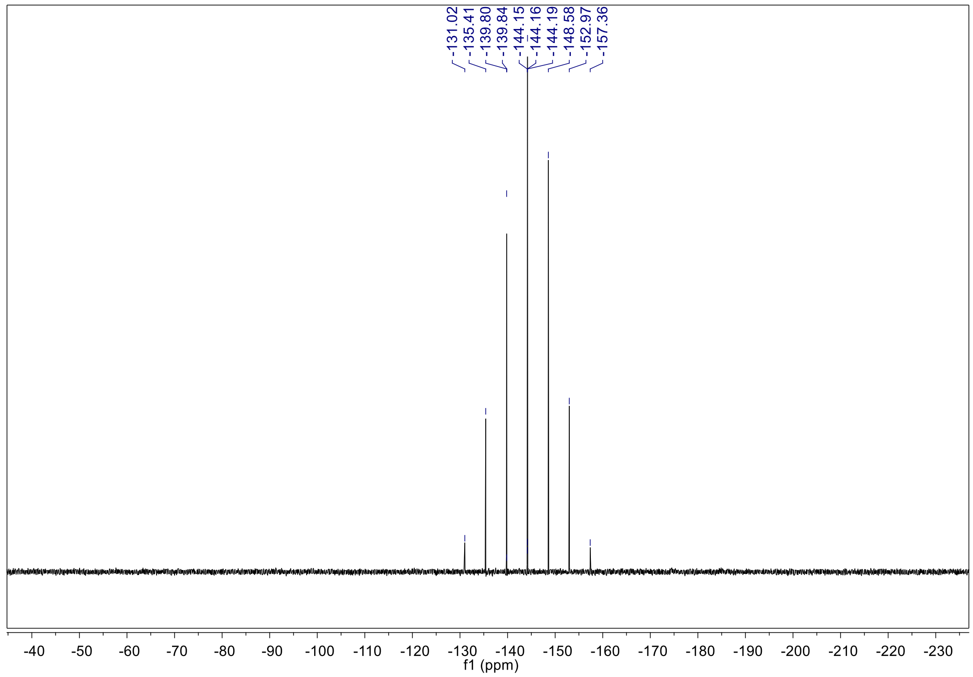

Figure S54. ^{31}P NMR of Compound **18**.

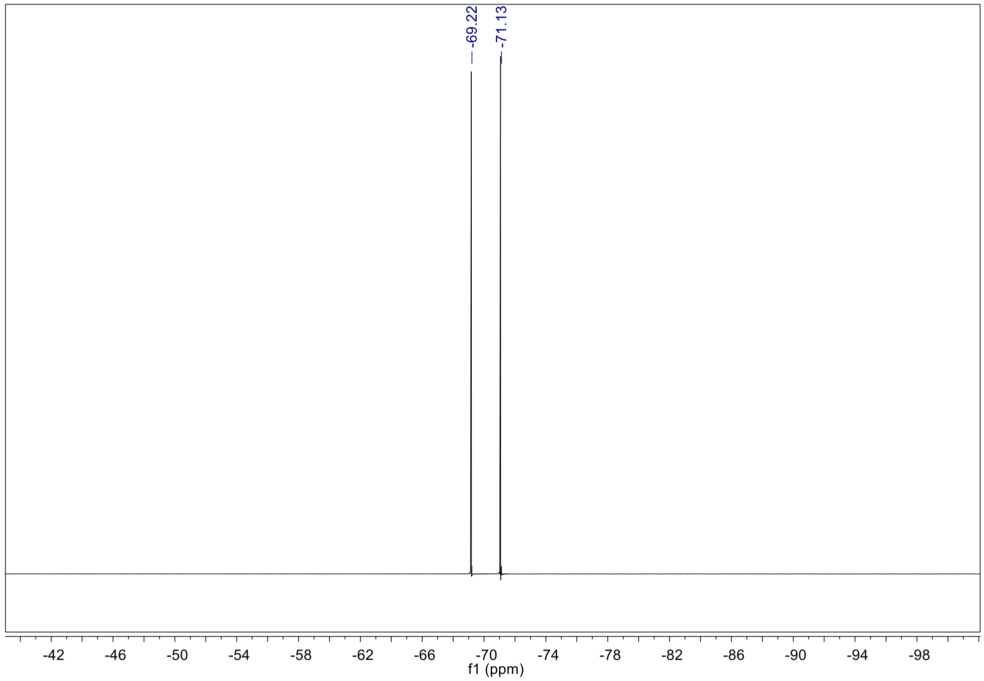

Figure S55. ^{19}F NMR of Compound **18**.

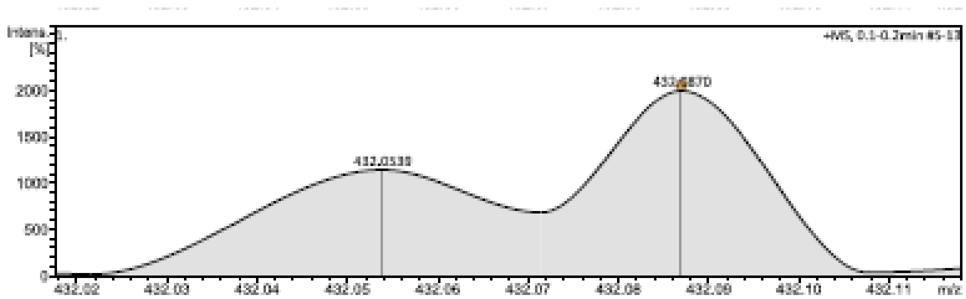

Figure S56. HRMS (ESI) of Compound **18**.

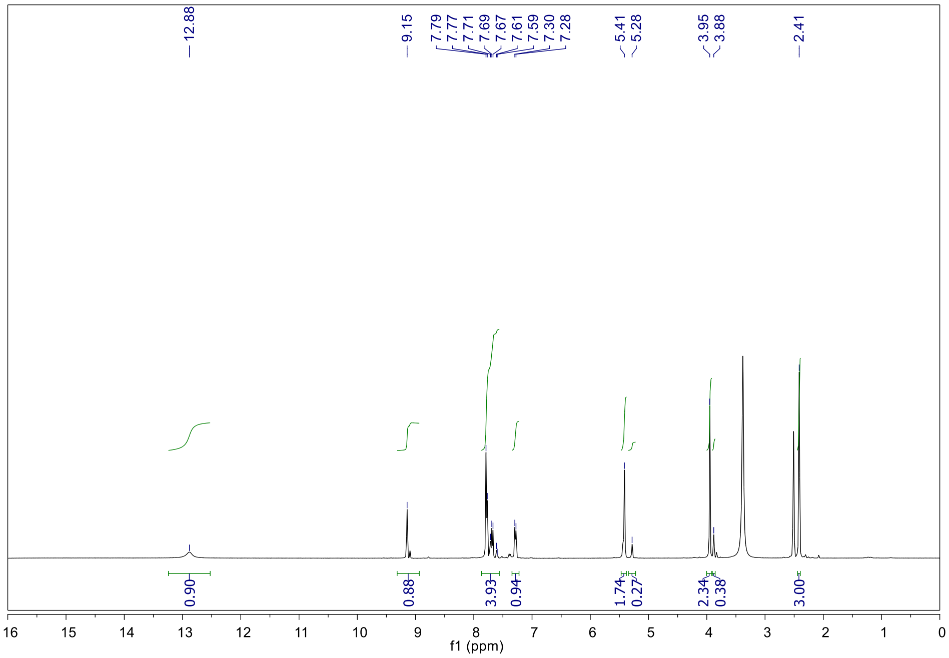

Figure S57. ^{1}H NMR of Compound **19**.

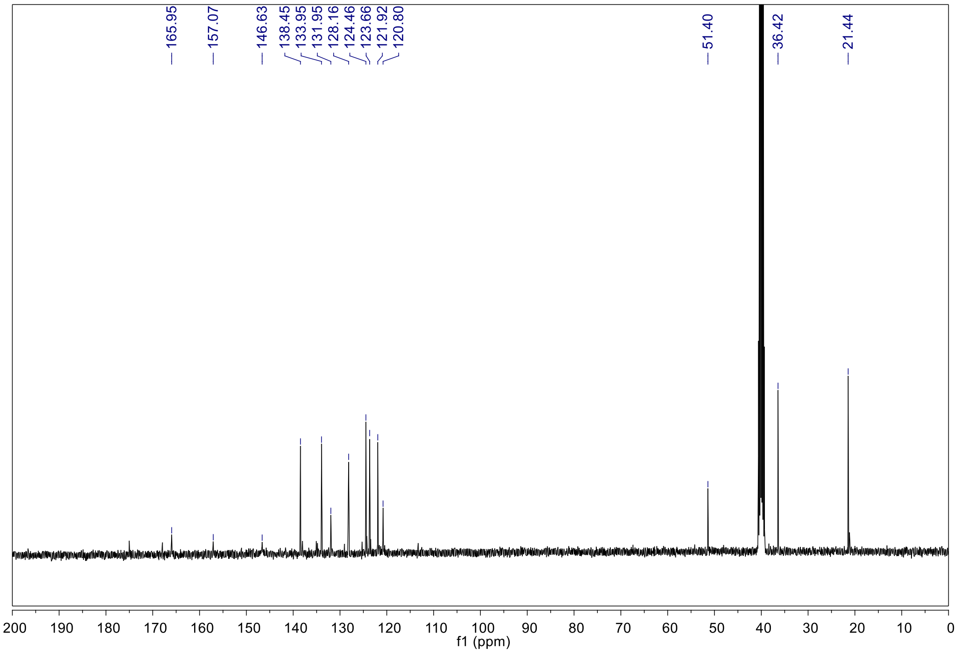

Figure S58. ^{13}C NMR of Compound **19**.

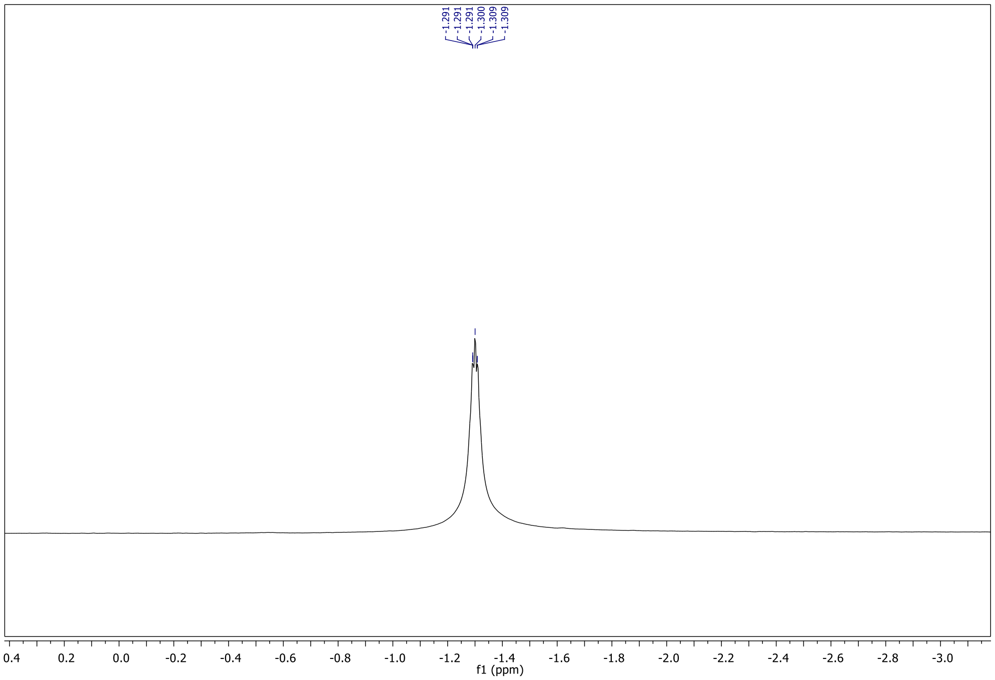

Figure S59. ^{11}B NMR of Compound **19**.

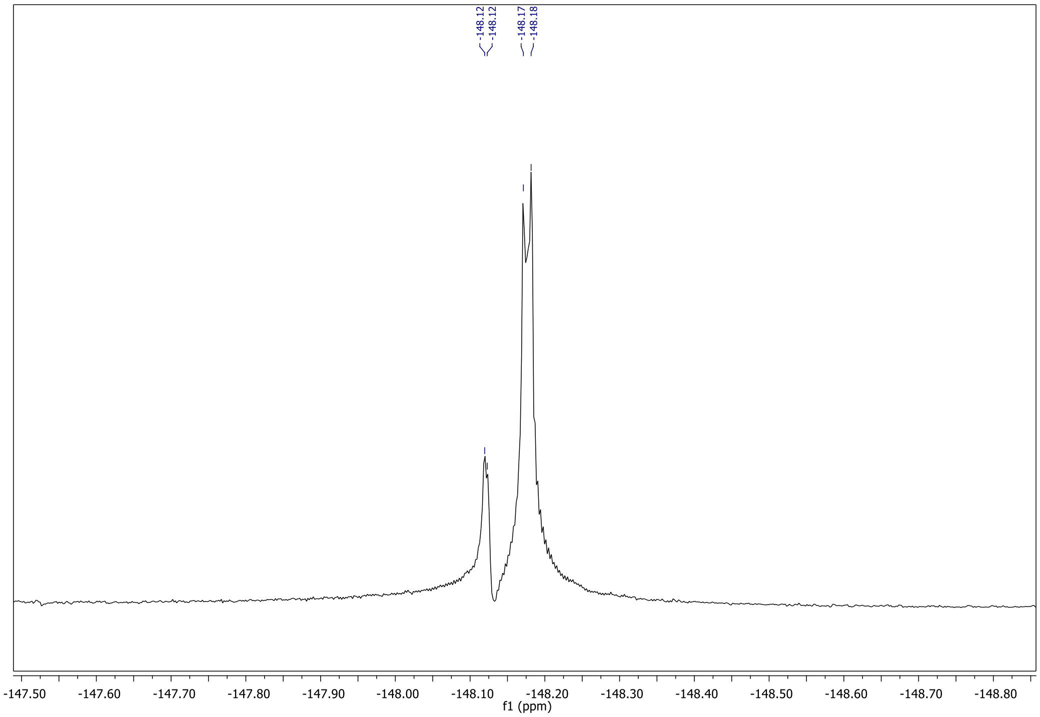

Figure S60. ^{19}F NMR of Compound **19**.

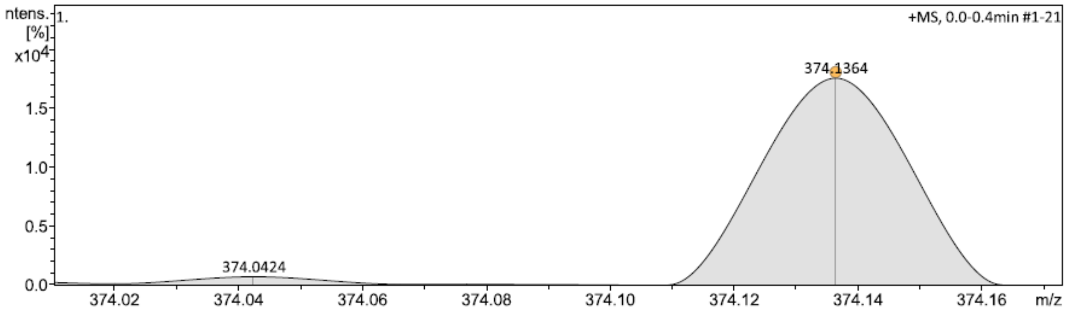

Figure S61. HRMS (ESI) of Compound **19**.

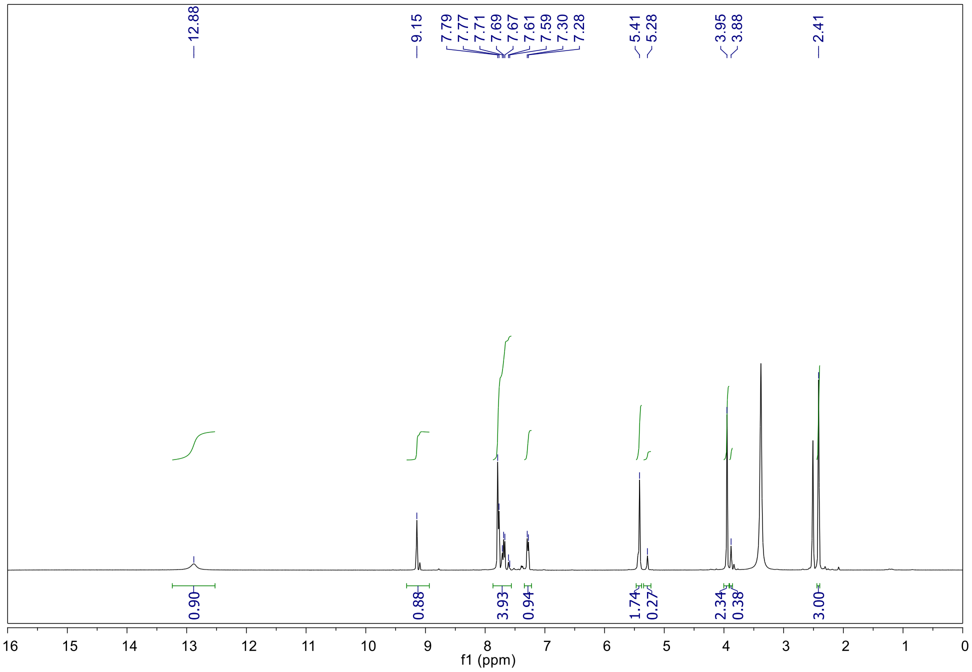

Figure S62. ^{1}H NMR of Compound **20**.

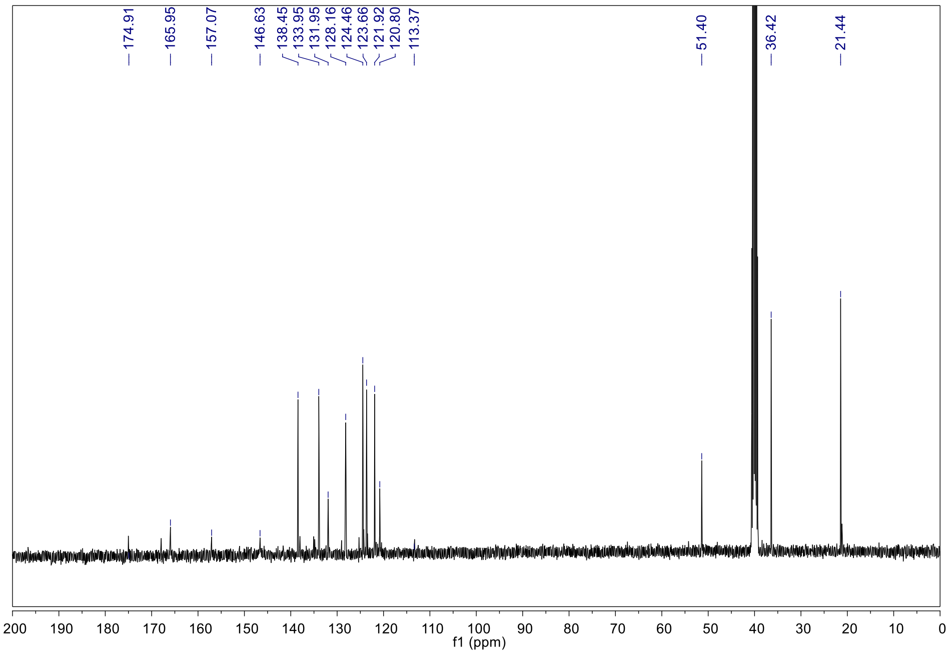

Figure S63. ^{13}C NMR of Compound **20**.

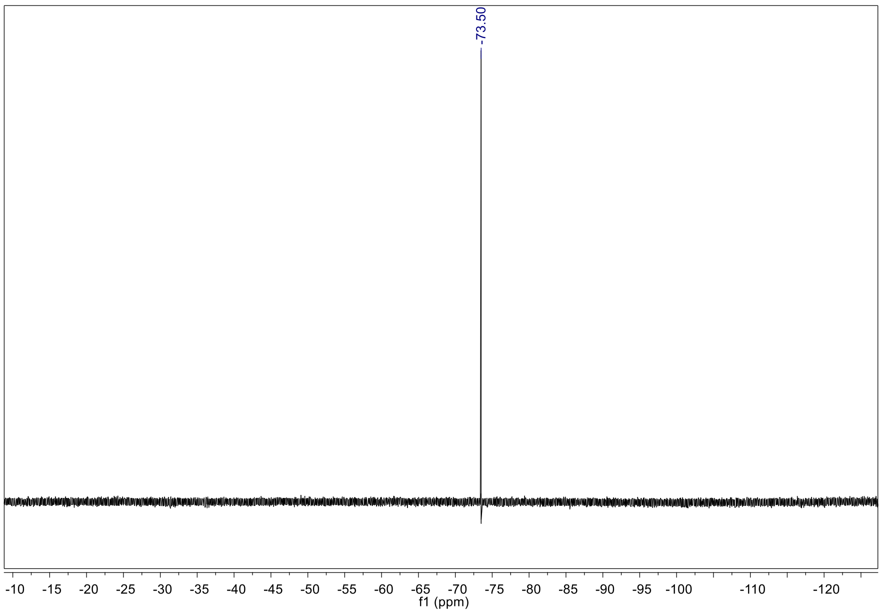

Figure S64. ^{19}F NMR of Compound **20**.

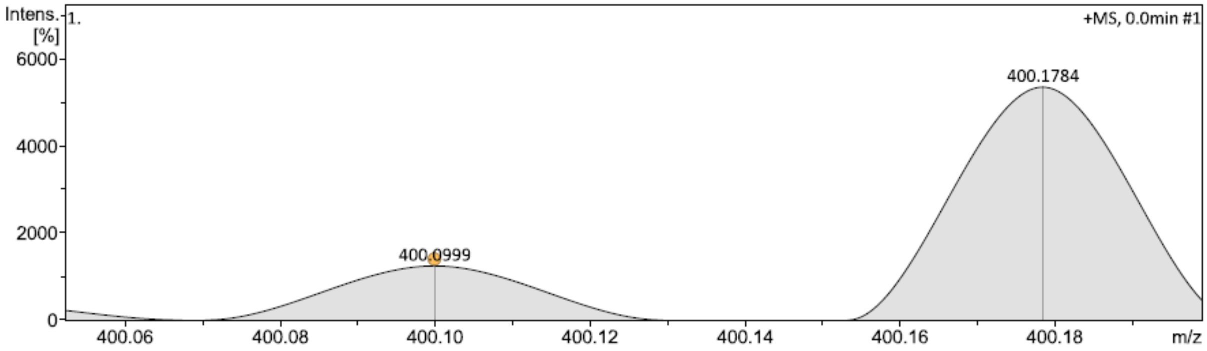

Figure S65. HRMS (ESI) of Compound **20**.

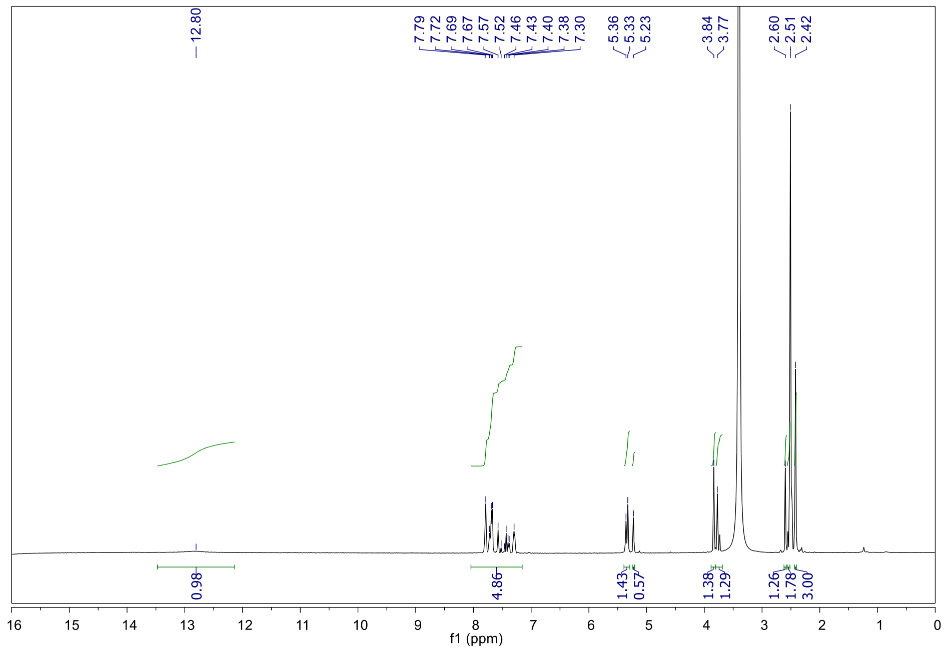

Figure S66. ^{1}H NMR of Compound **21**.

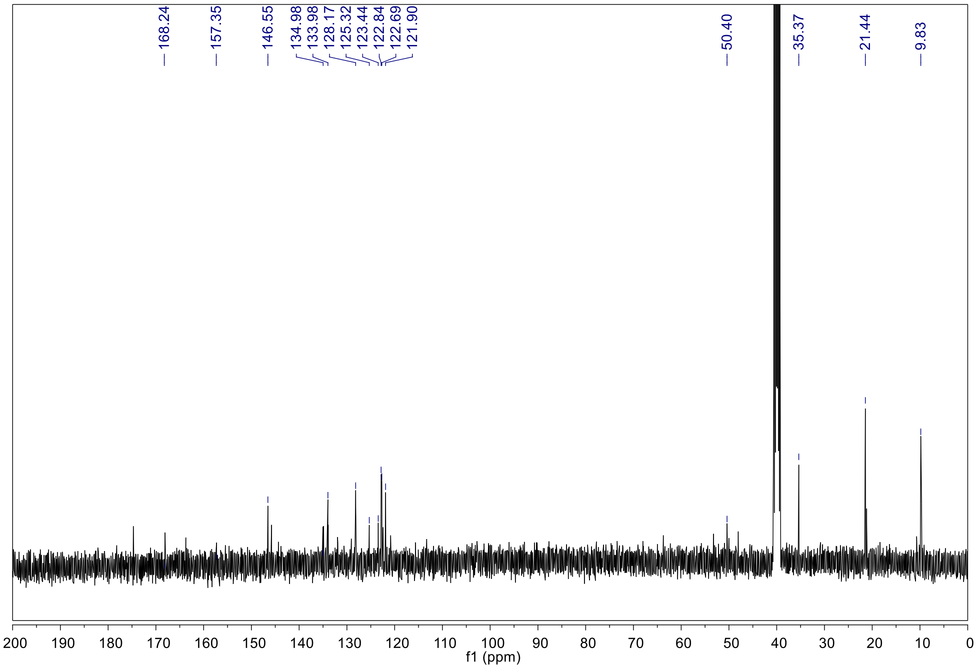

Figure S67. ^{13}C NMR of Compound **21**.

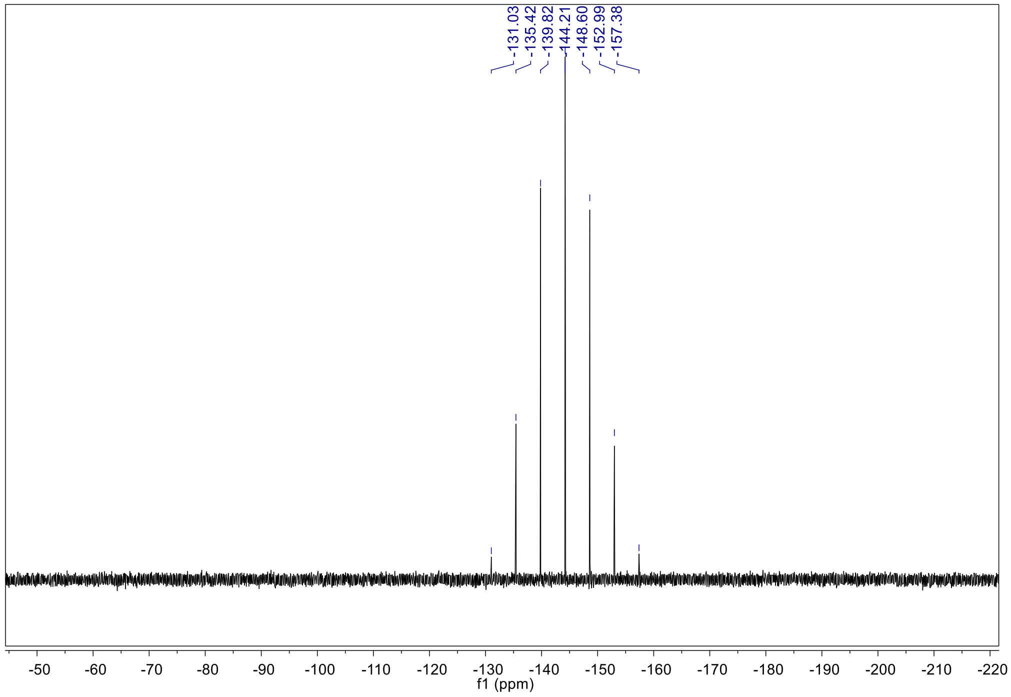

Figure S68. ^{31}P NMR of Compound **21**.

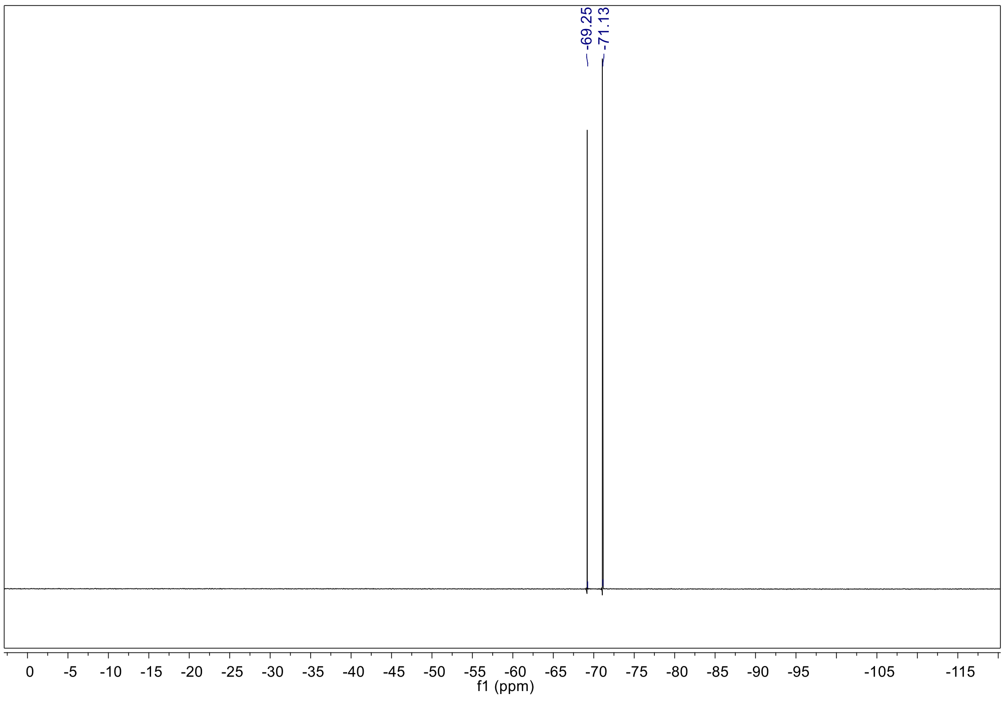

Figure S69. ^{19}F NMR of Compound **21**.

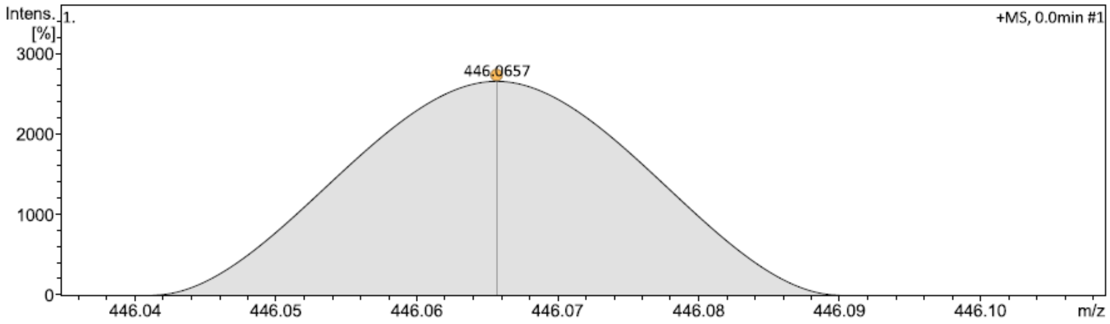

Figure S70. HRMS (ESI) of Compound **21**.

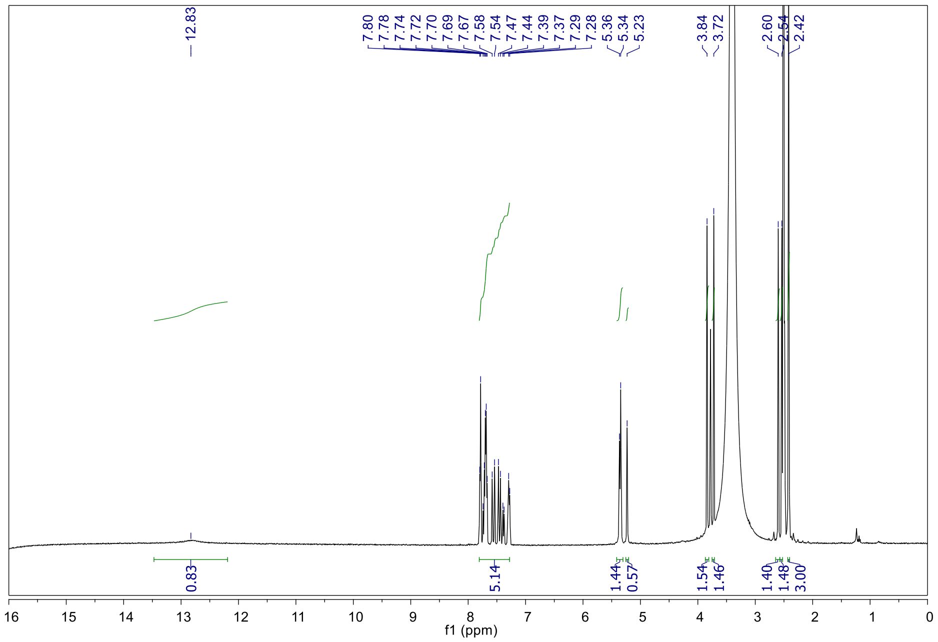

Figure S71. ^{1}H NMR of Compound **22**.

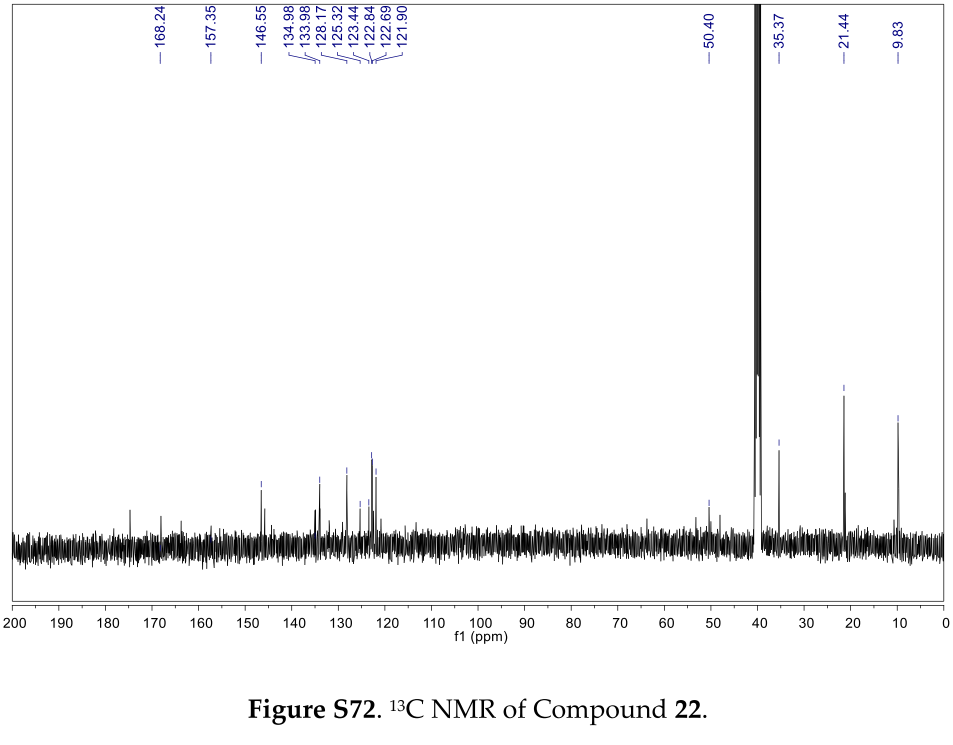

Figure S72. ^{13}C NMR of Compound **22**.

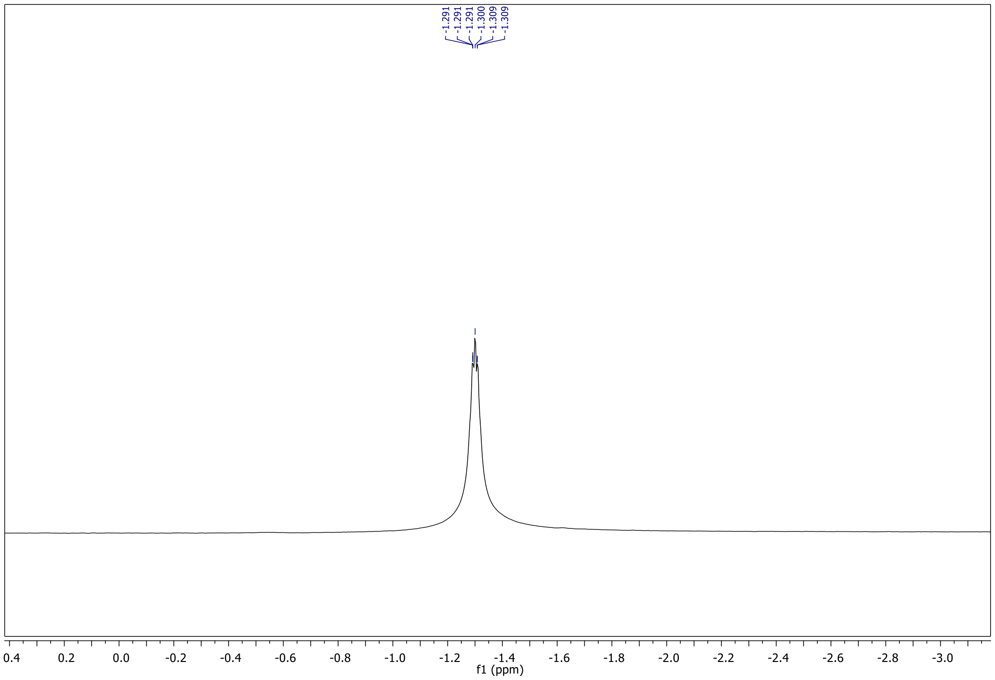

Figure S73. ^{11}B NMR of Compound **22**.

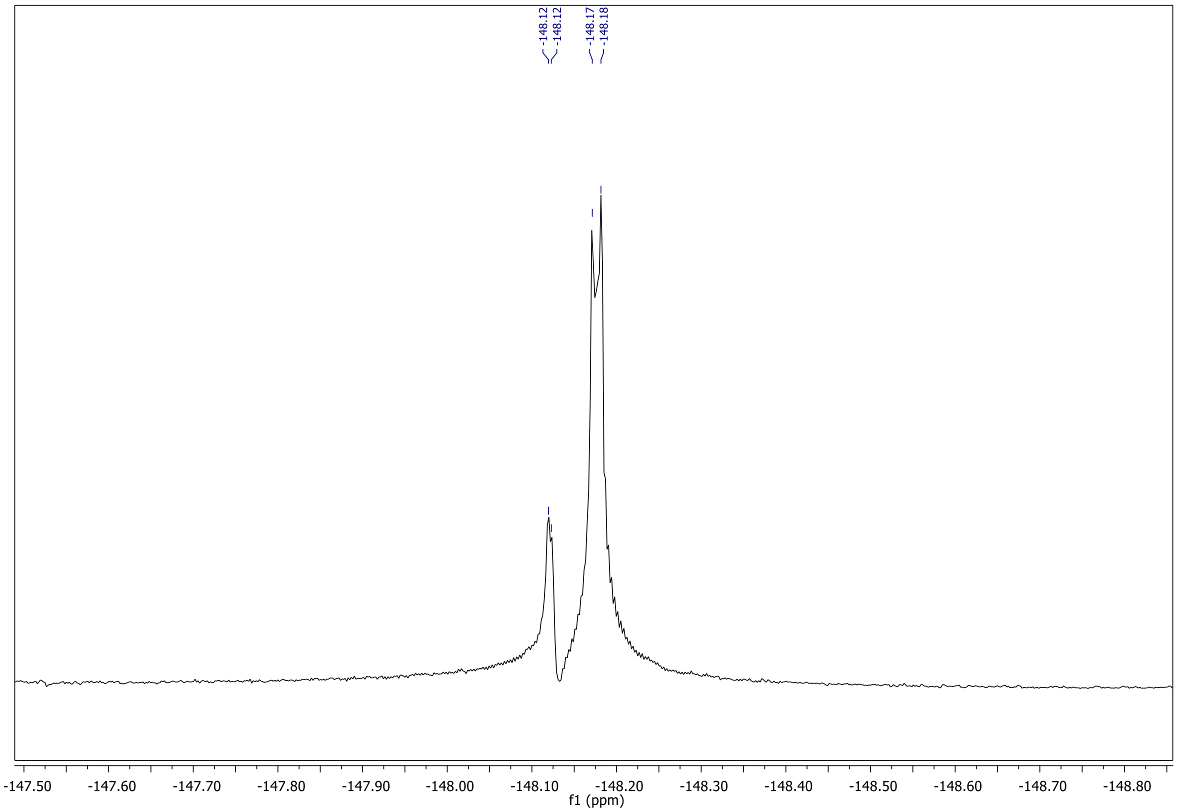

Figure S74. ^{19}F NMR of Compound **22**.

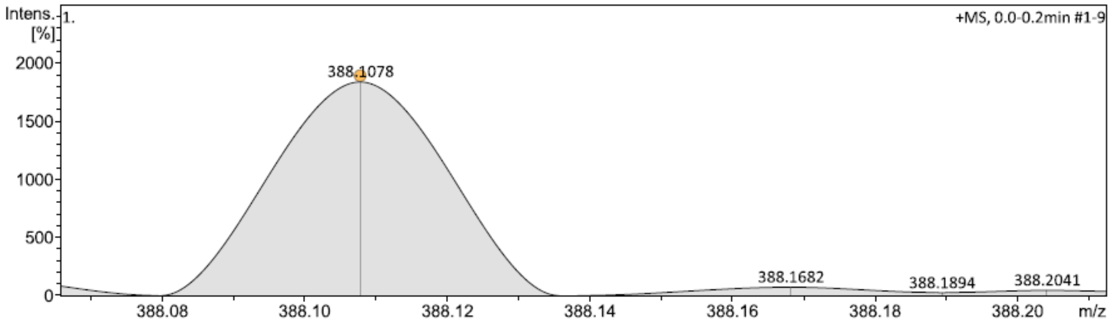

Figure S75. HRMS (ESI) of Compound **22**.

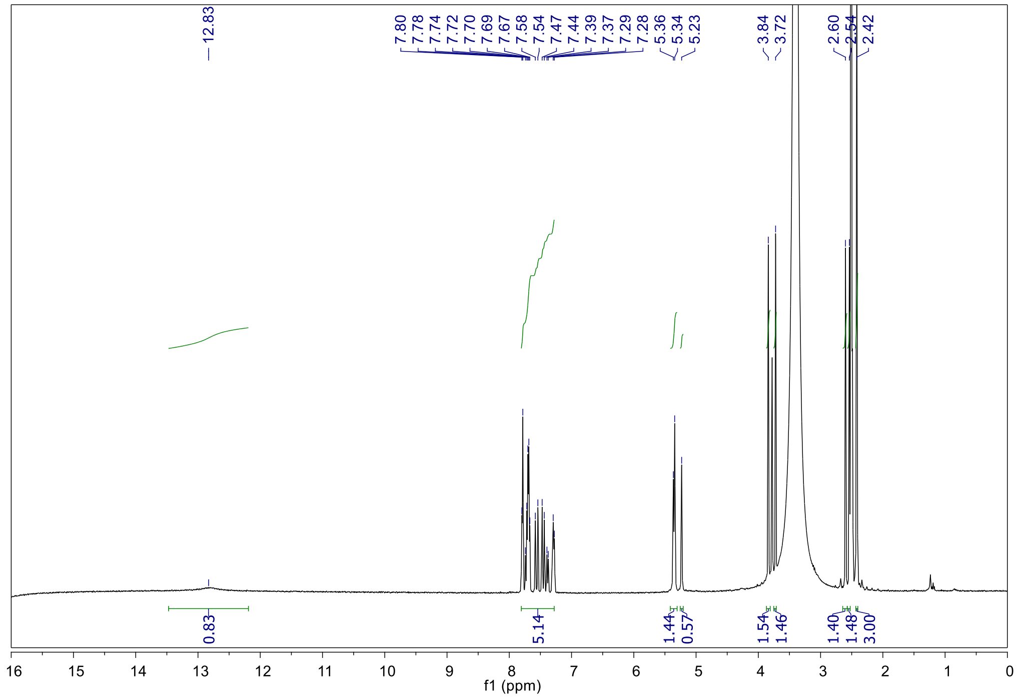

Figure S76. ^{1}H NMR of Compound **23**.

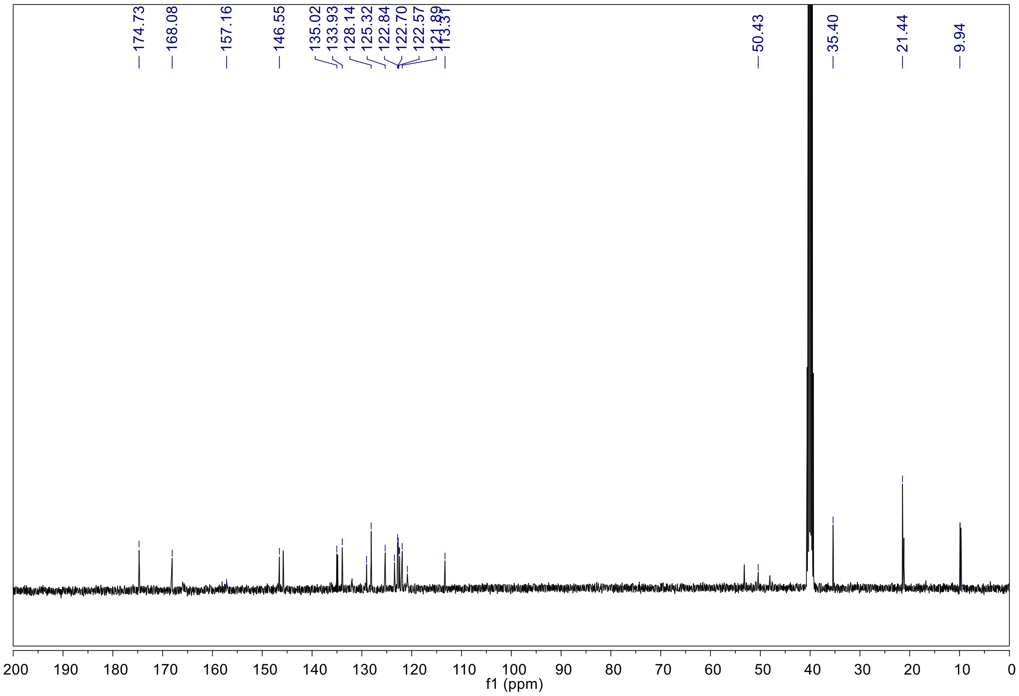

Figure S77. ^{13}C NMR of Compound **23**.

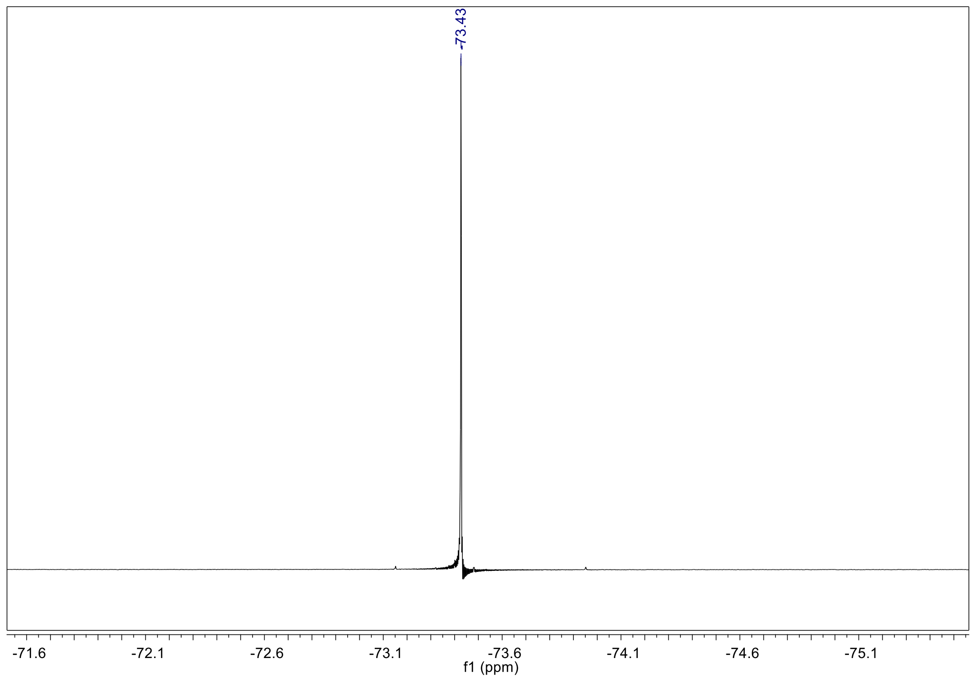

Figure S78. ^{19}F NMR of Compound **23**.

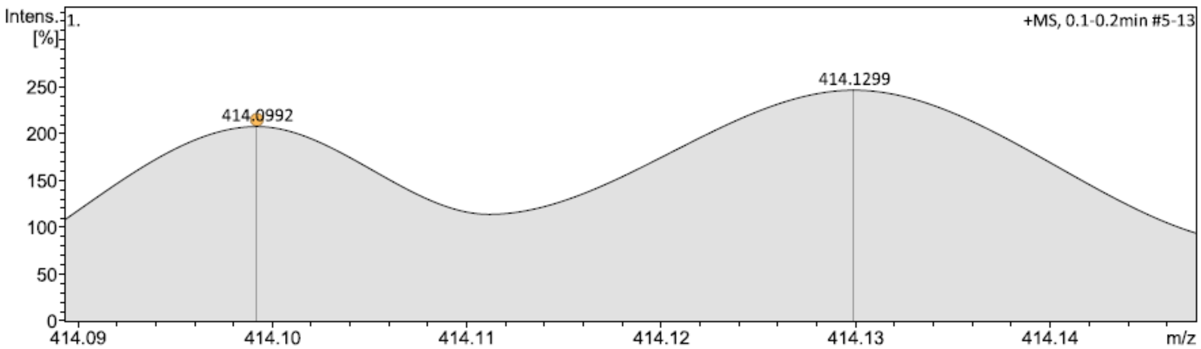

Figure S79. HRMS (ESI) of Compound **23**.

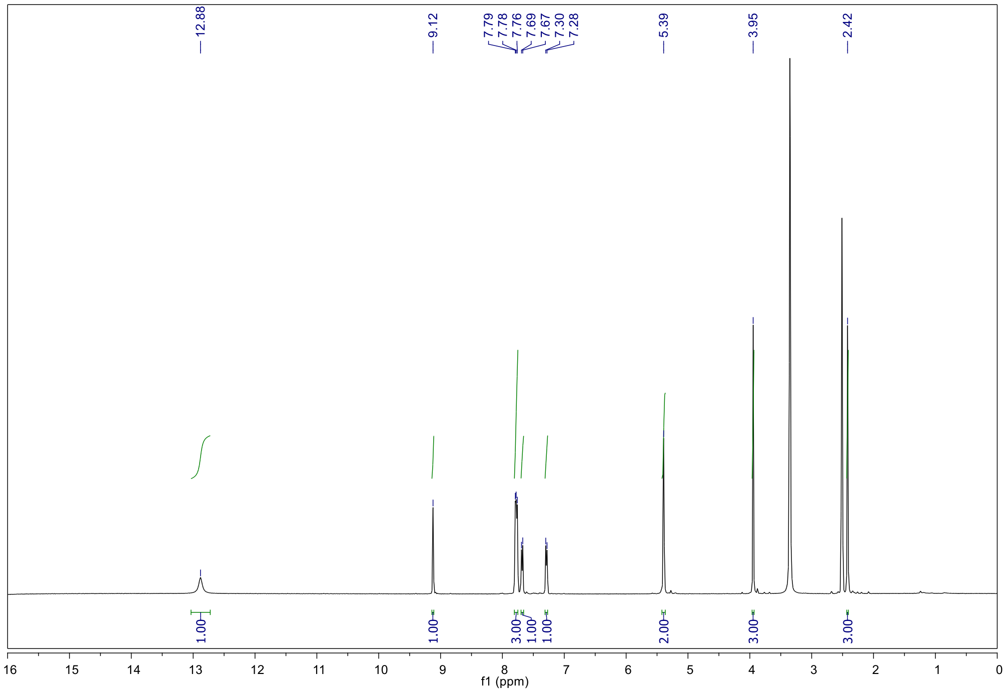

Figure S80. ^{1}H NMR of Compound **24**.

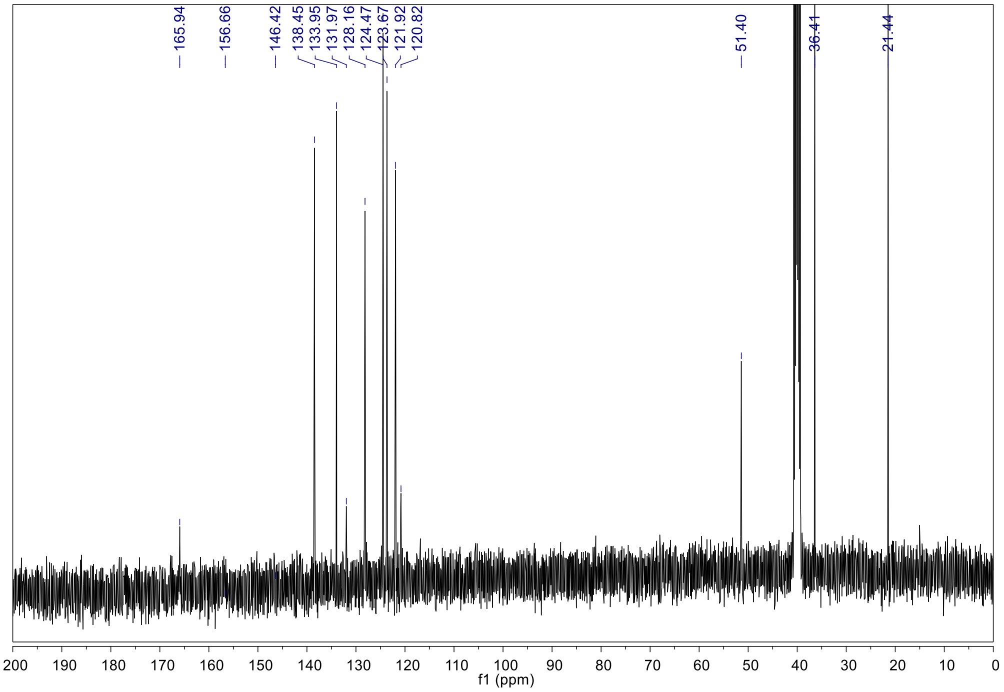

Figure S81. ^{13}C NMR of Compound **24**.

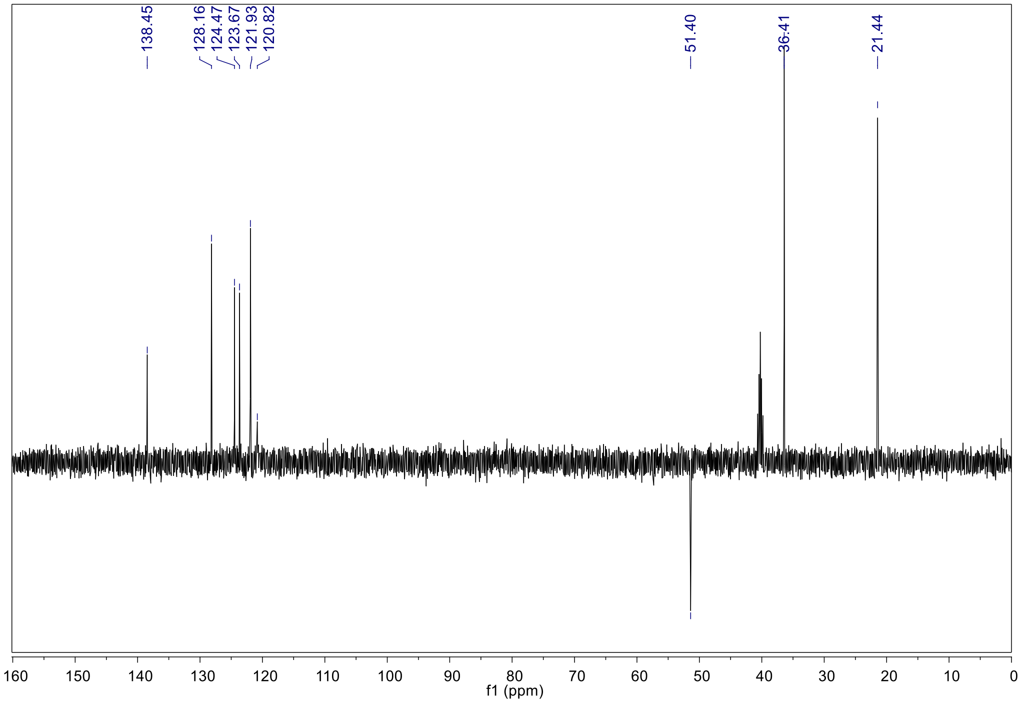

Figure S82. Dept-135 NMR of Compound **24**.

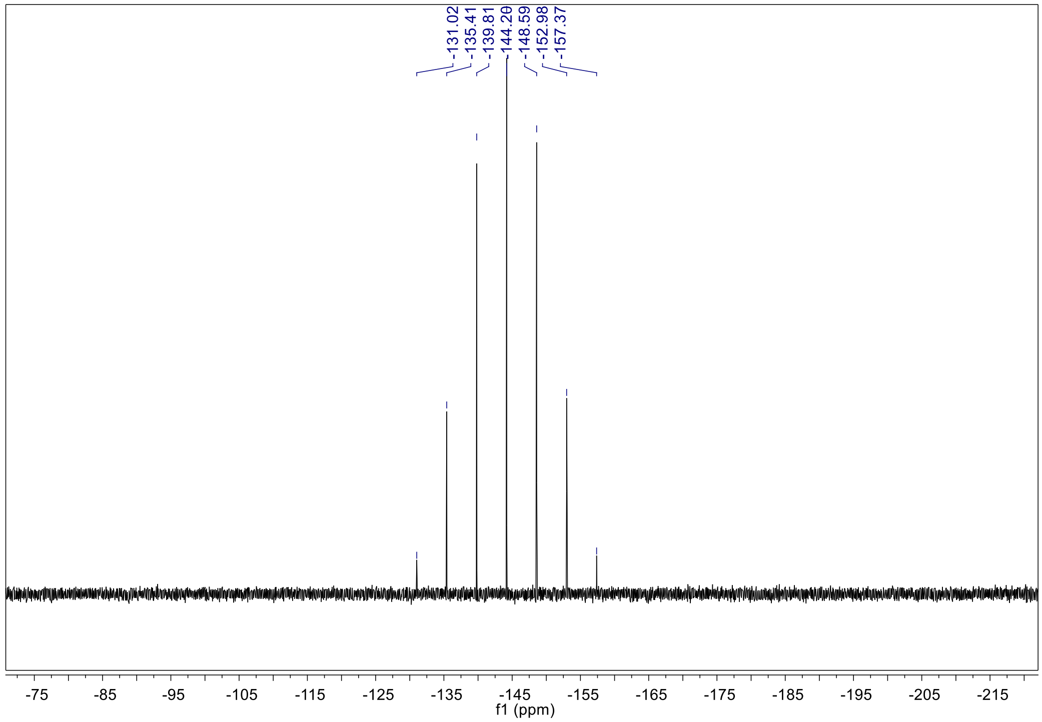

Figure S83. ^{31}P NMR of Compound **24**.

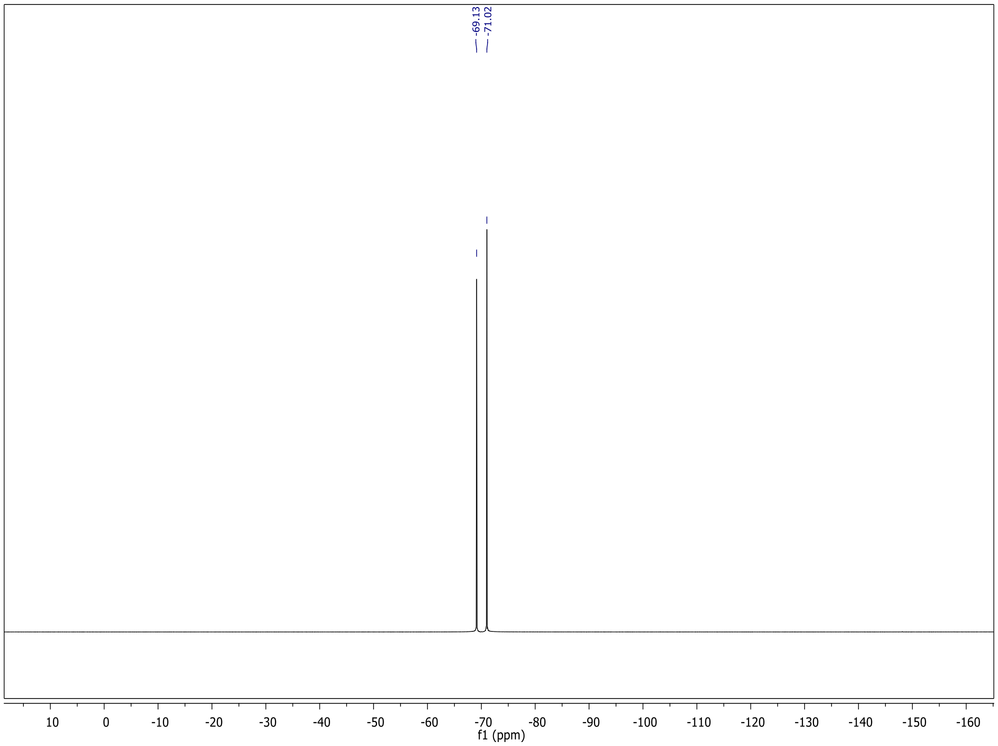

Figure S84. ^{19}F NMR of Compound **24**.

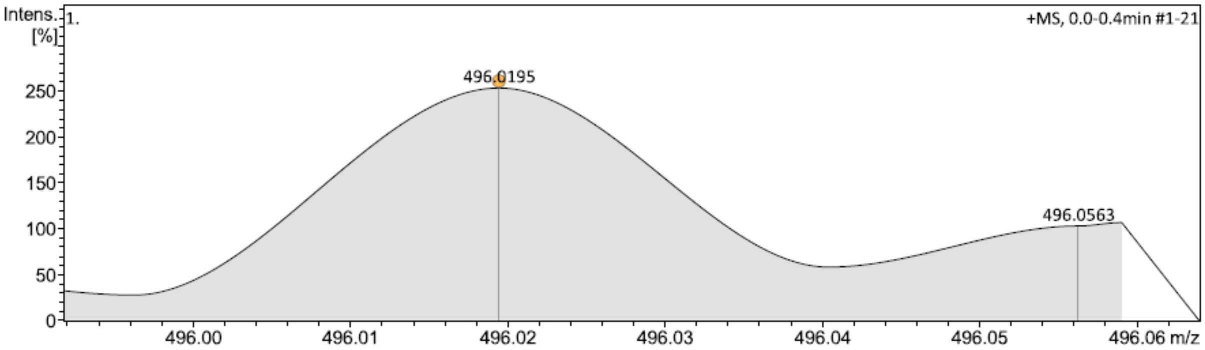

Figure S85. HRMS (ESI) of Compound **24**.

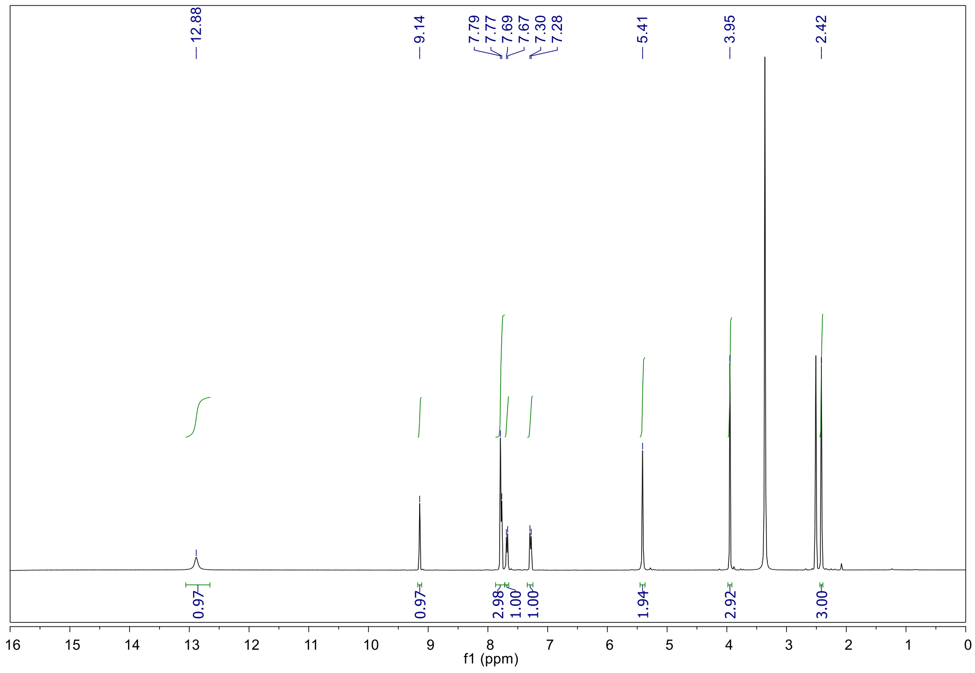

Figure S86. ^{1}H NMR of Compound **25**.

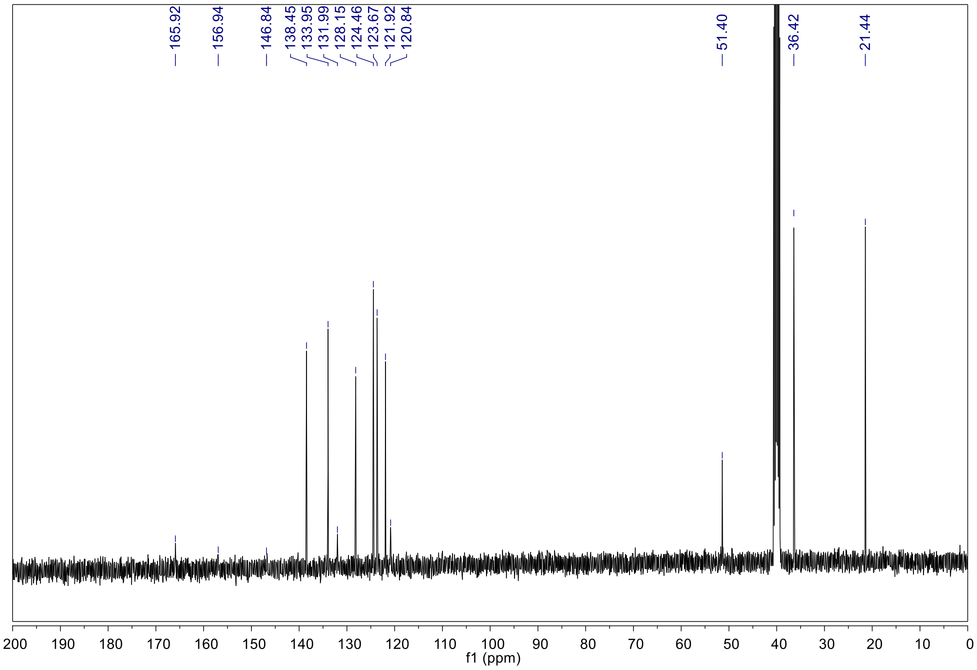

Figure S87. ^{13}C NMR of Compound **25**.

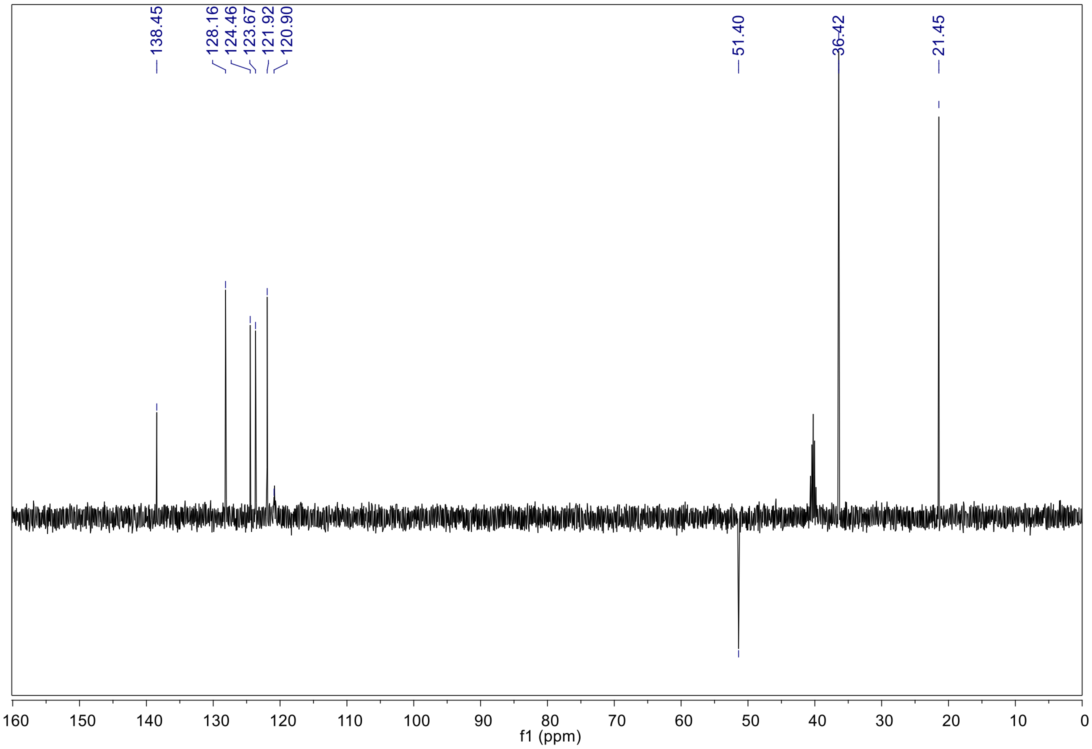

Figure S88. Dept-135 NMR of Compound **25**.

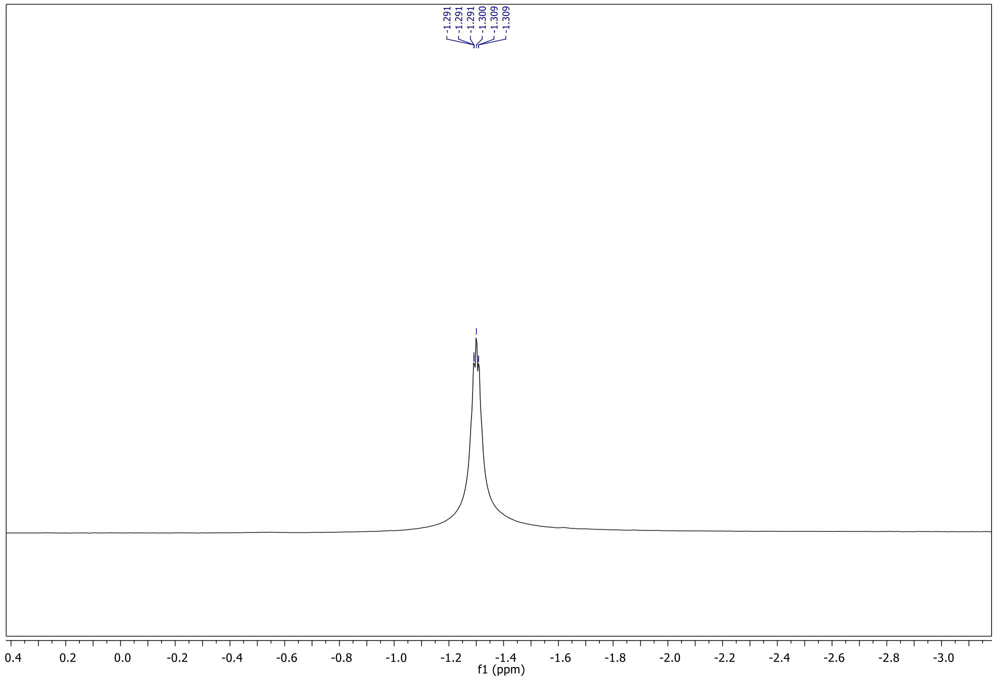

Figure S89. ^{11}B NMR of Compound **25**.

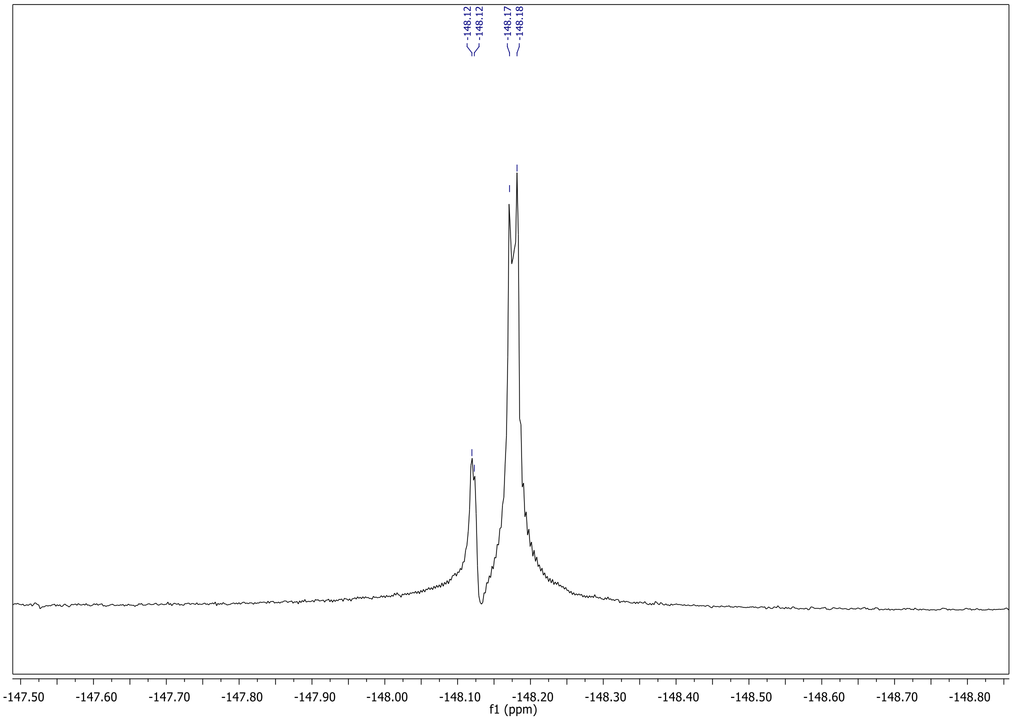

Figure S90. ^{19}F NMR of Compound **25**.

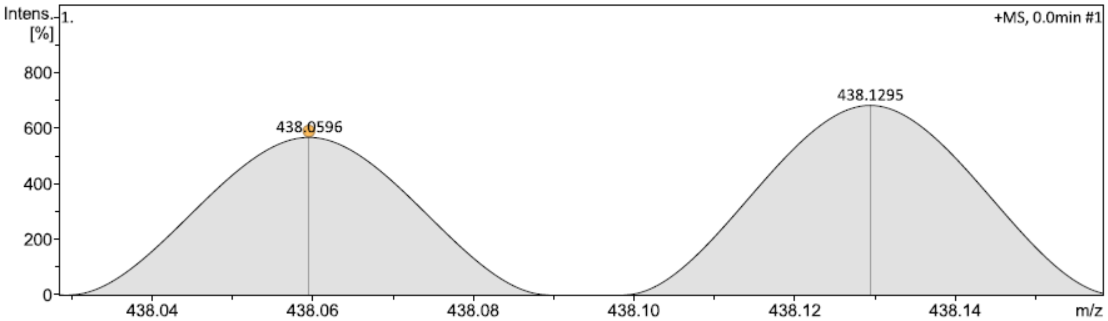

Figure S91. HRMS (ESI) of Compound **25**.

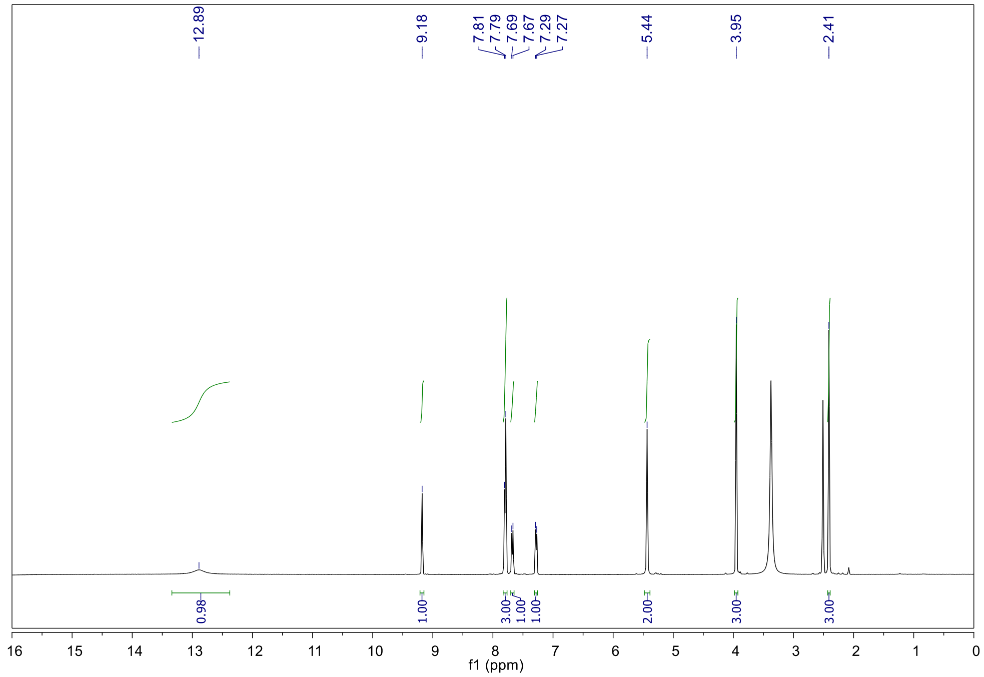

Figure S92. ^{1}H NMR of Compound **26**.

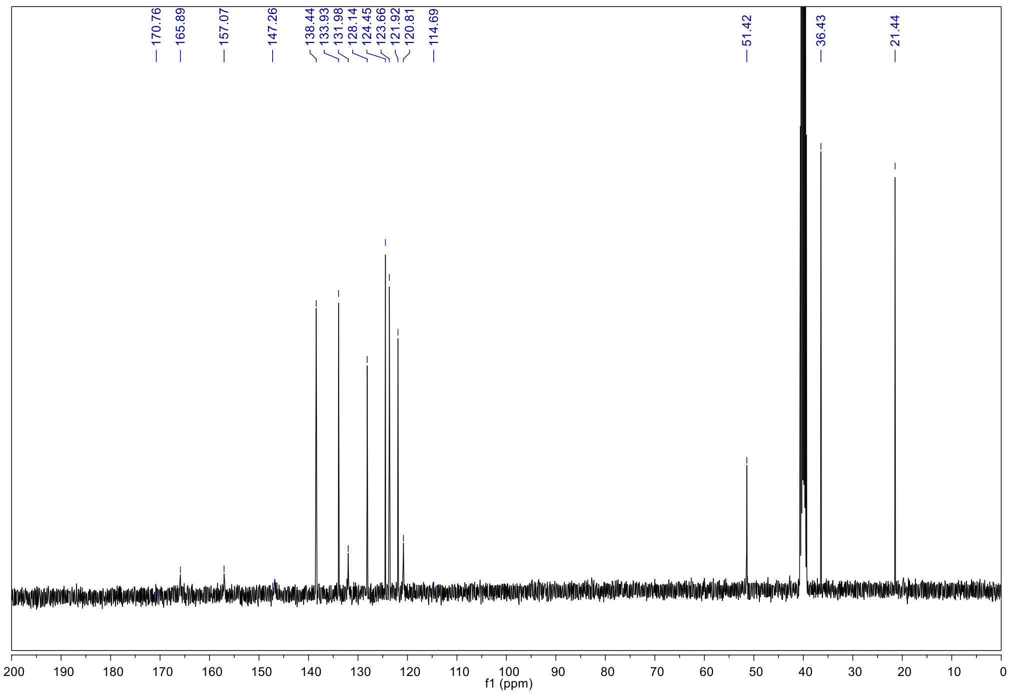

Figure S93. ^{13}C NMR of Compound **26**.

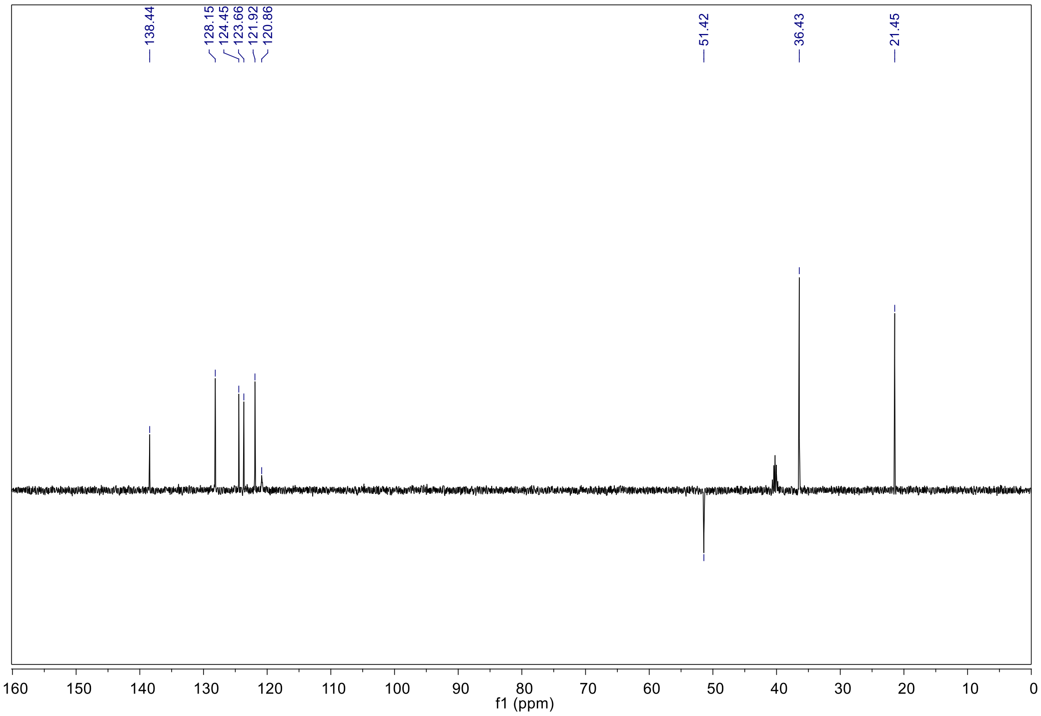

Figure S94. Dept-135 NMR of Compound **26**.

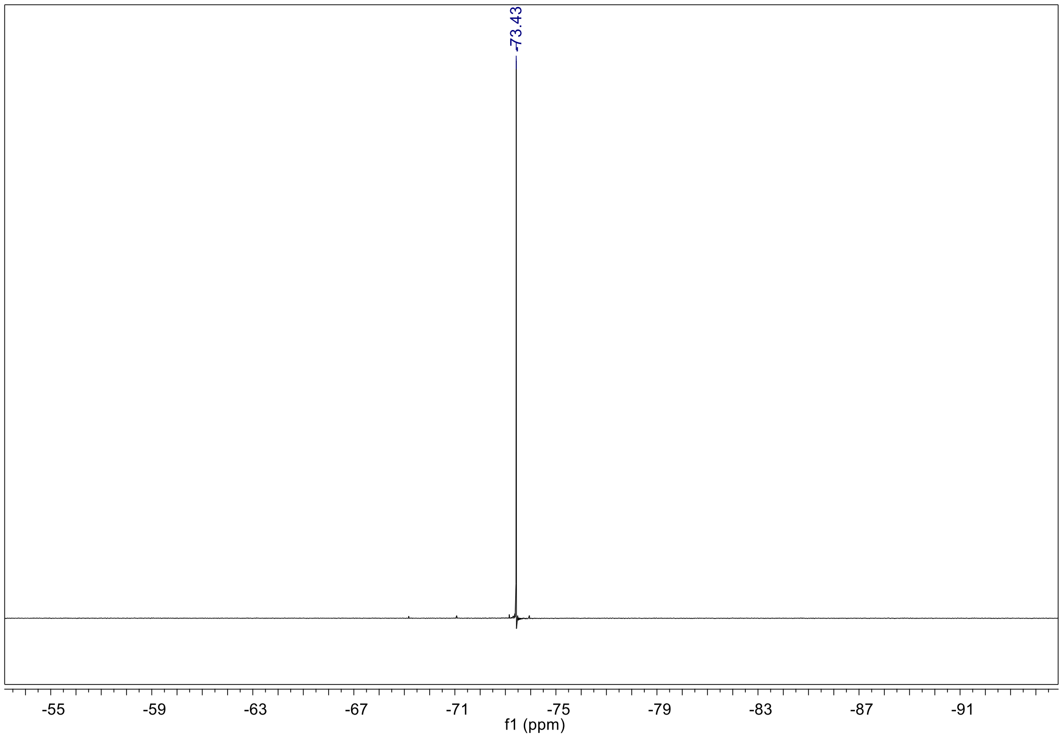

Figure S95. ^{19}F NMR of Compound **26**.

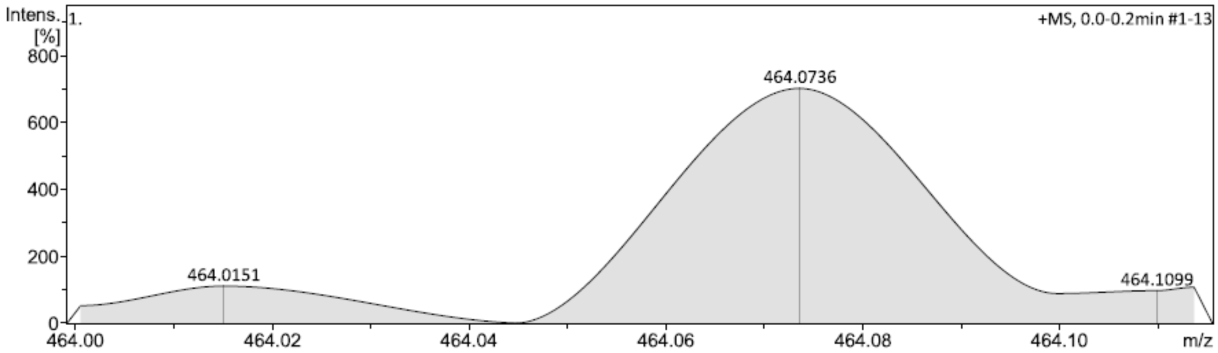

Figure S96. HRMS (ESI) of Compound **26**.

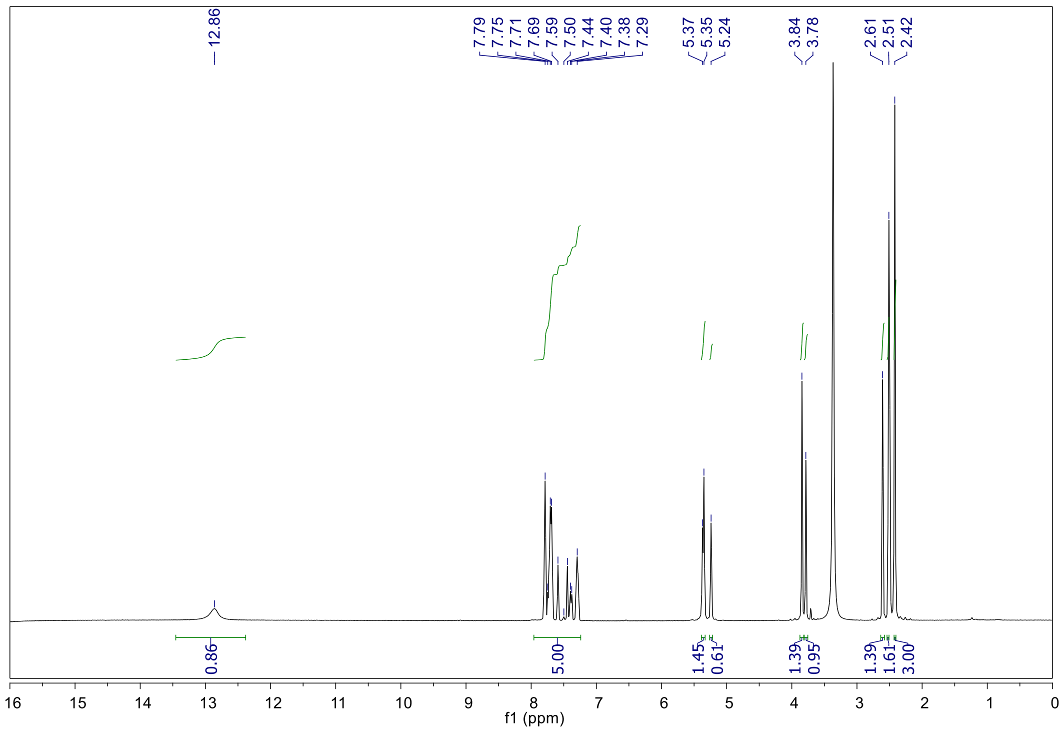

Figure S97. ^{1}H NMR of Compound **27**.

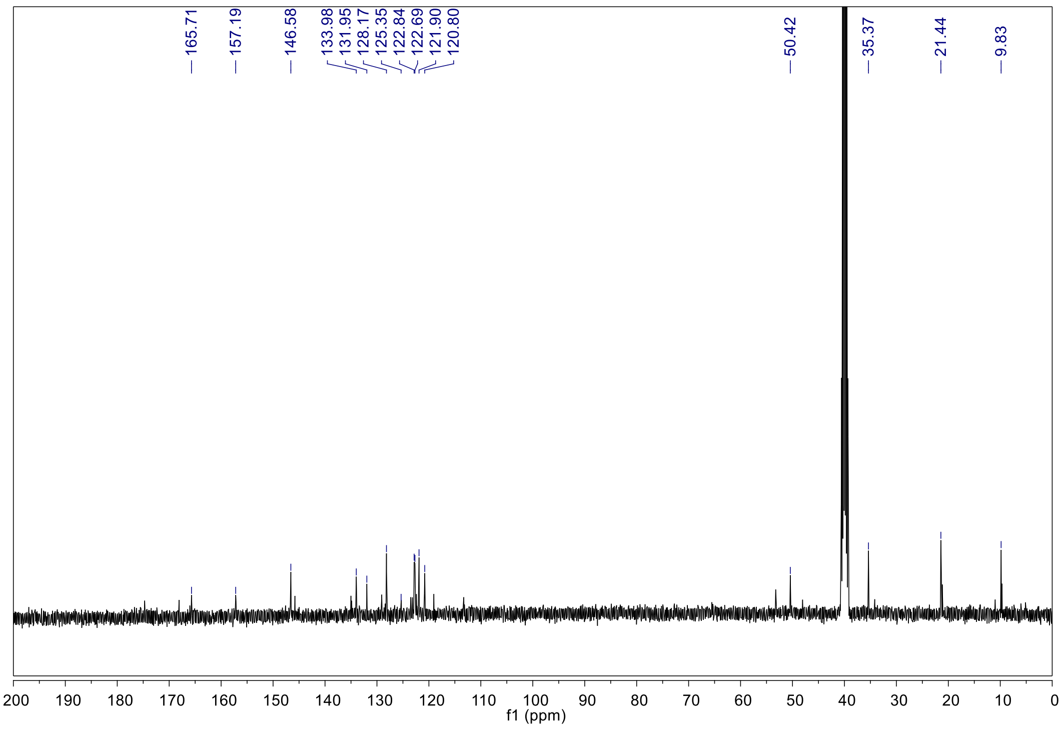

Figure S98. ^{13}C NMR of Compound **27**.

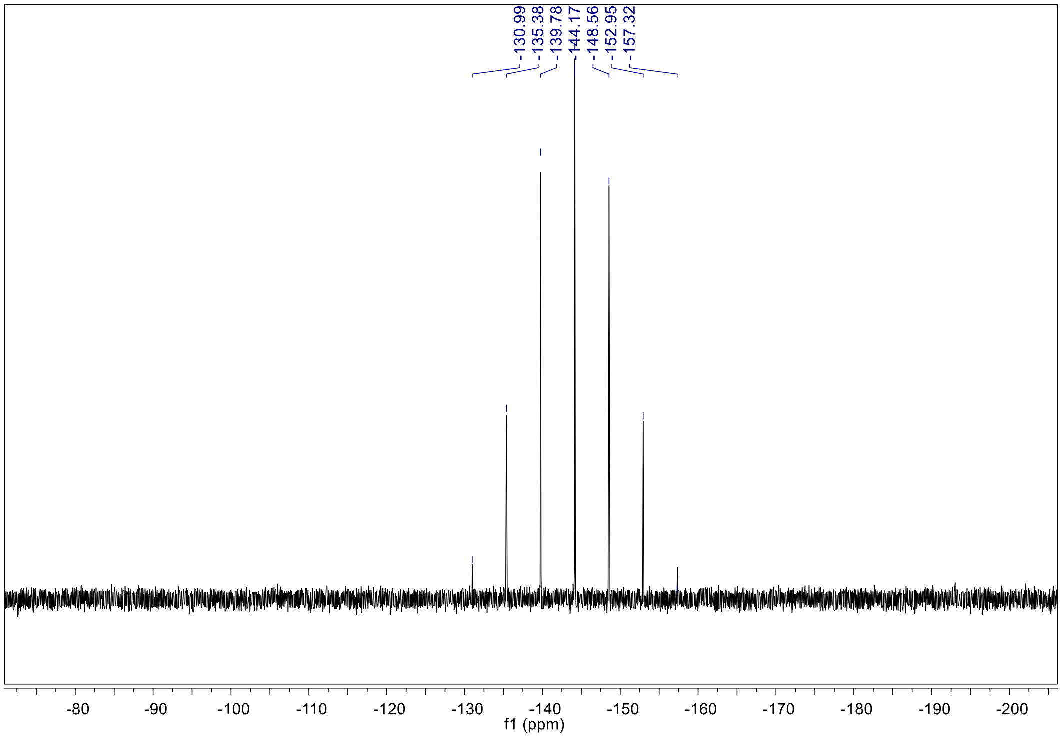

Figure S99. ^{31}P NMR of Compound **27**.

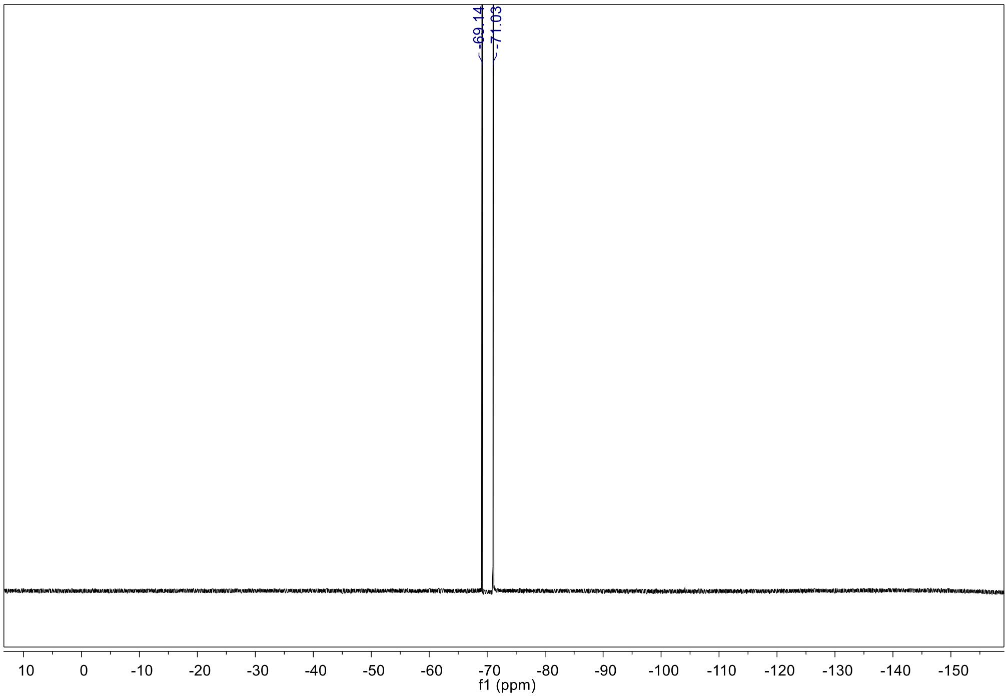

Figure S100. ^{19}F NMR of Compound **27**.

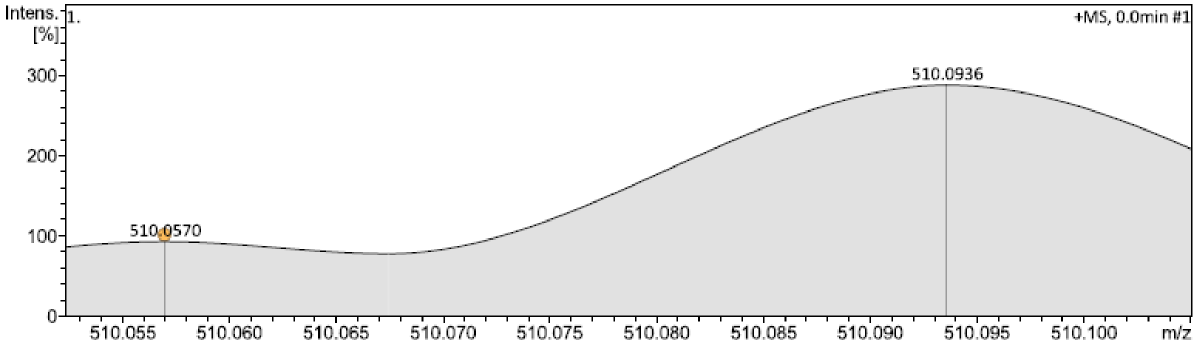

Figure S101. HRMS (ESI) of Compound **27**.

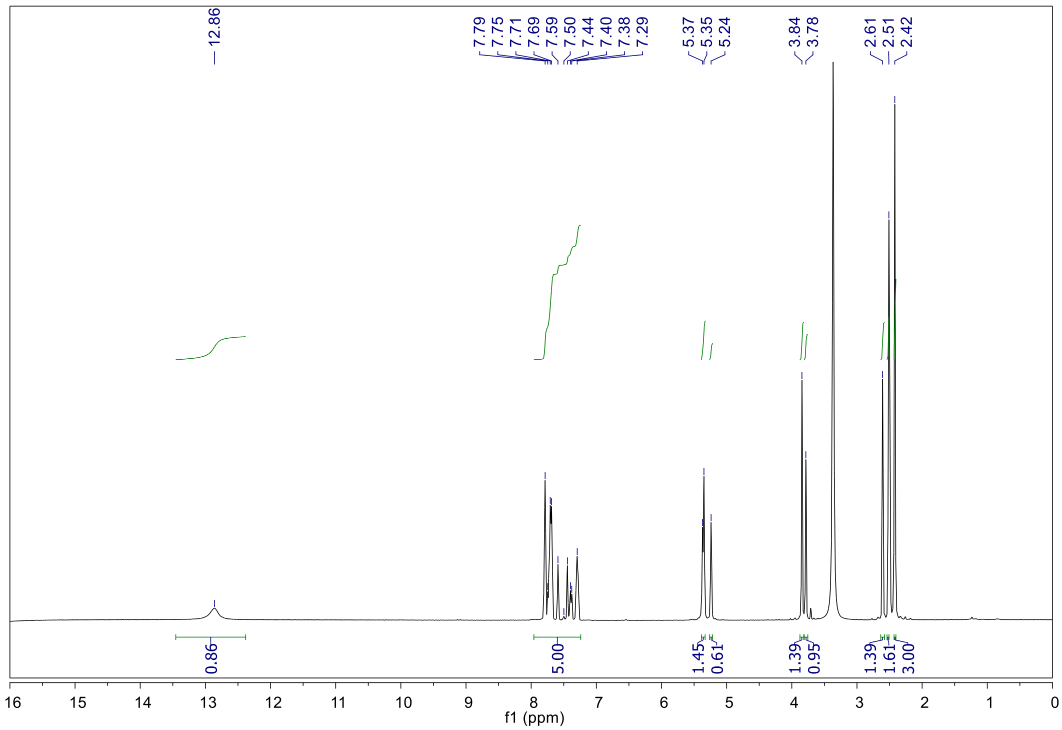

Figure S102. ^{1}H NMR of Compound **28**.

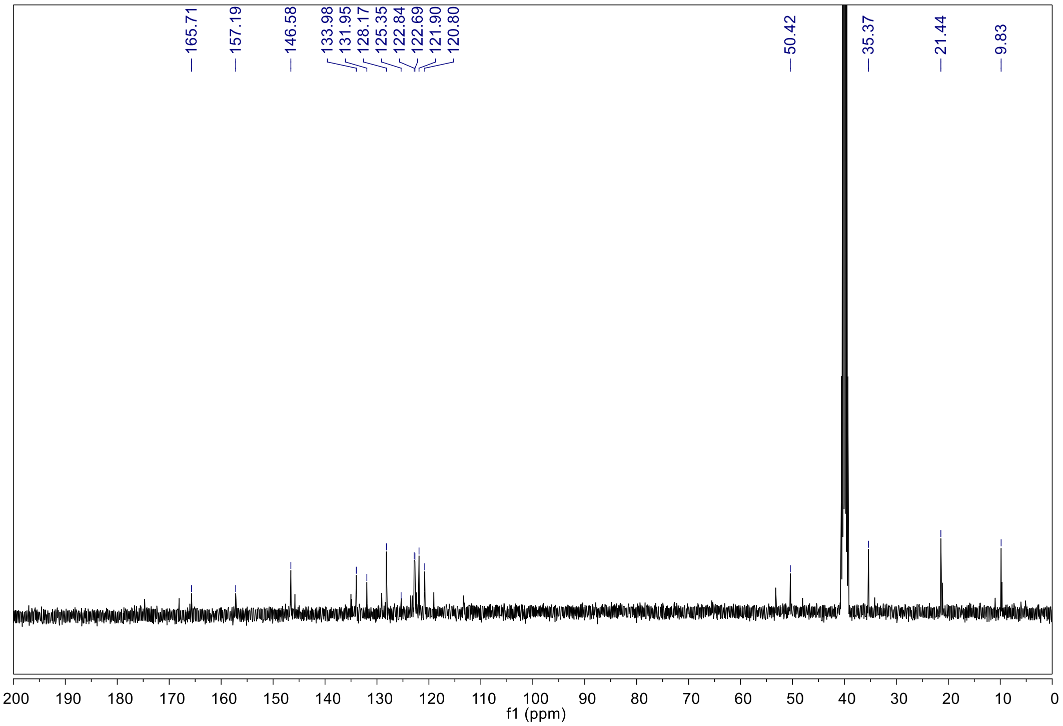

Figure S103. ^{13}C NMR of Compound **28**.

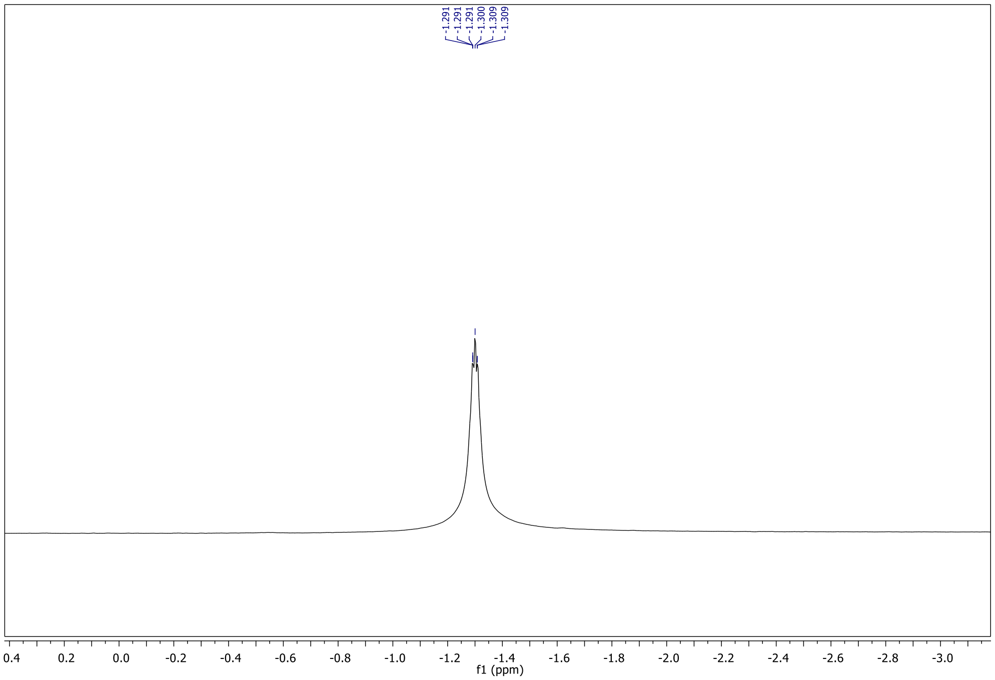

Figure S104. ^{11}B NMR of Compound **28**.

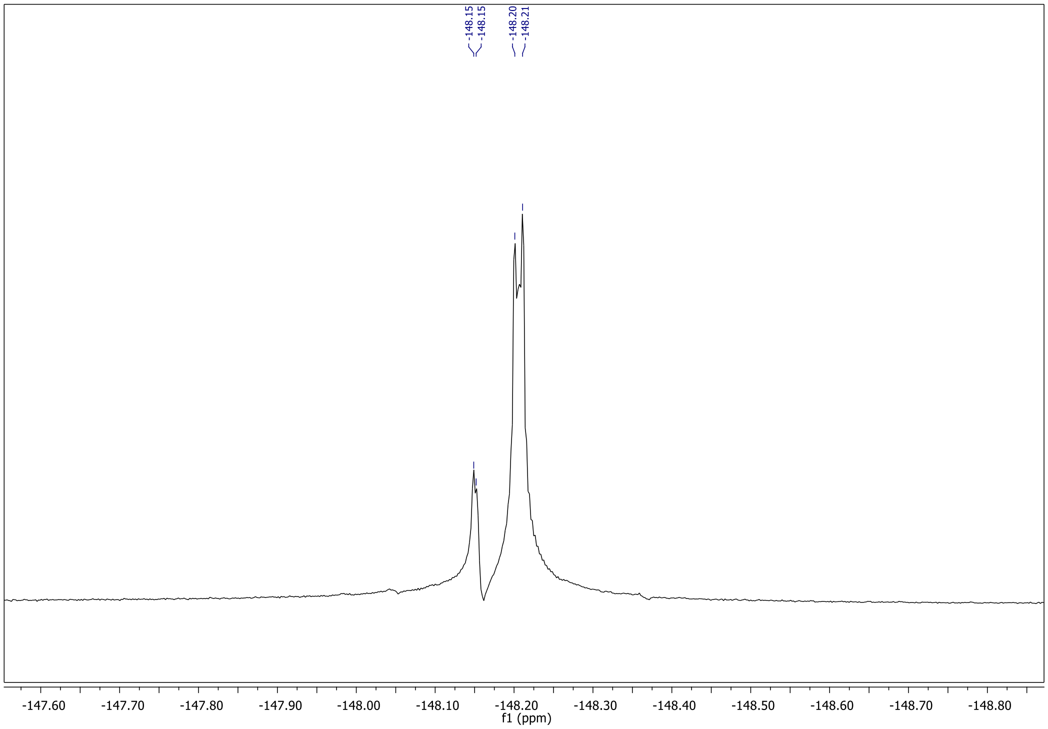

Figure S105. ^{19}F NMR of Compound **28**.

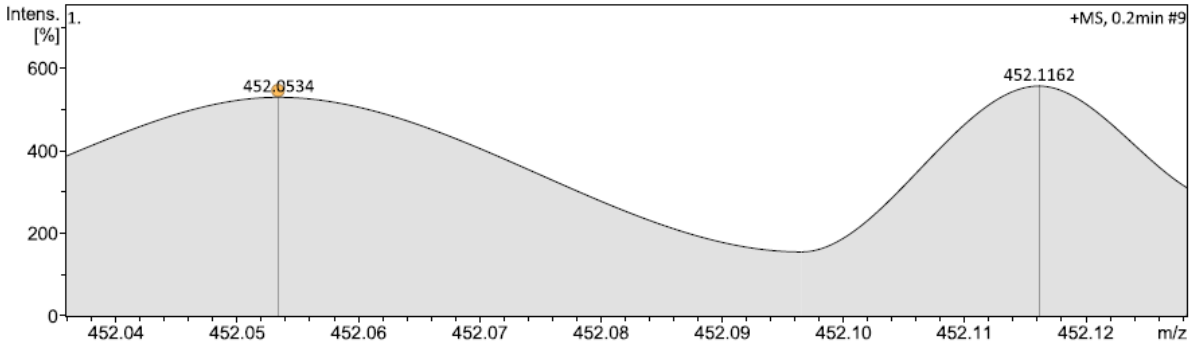

Figure S106. HRMS (ESI) of Compound **28**.

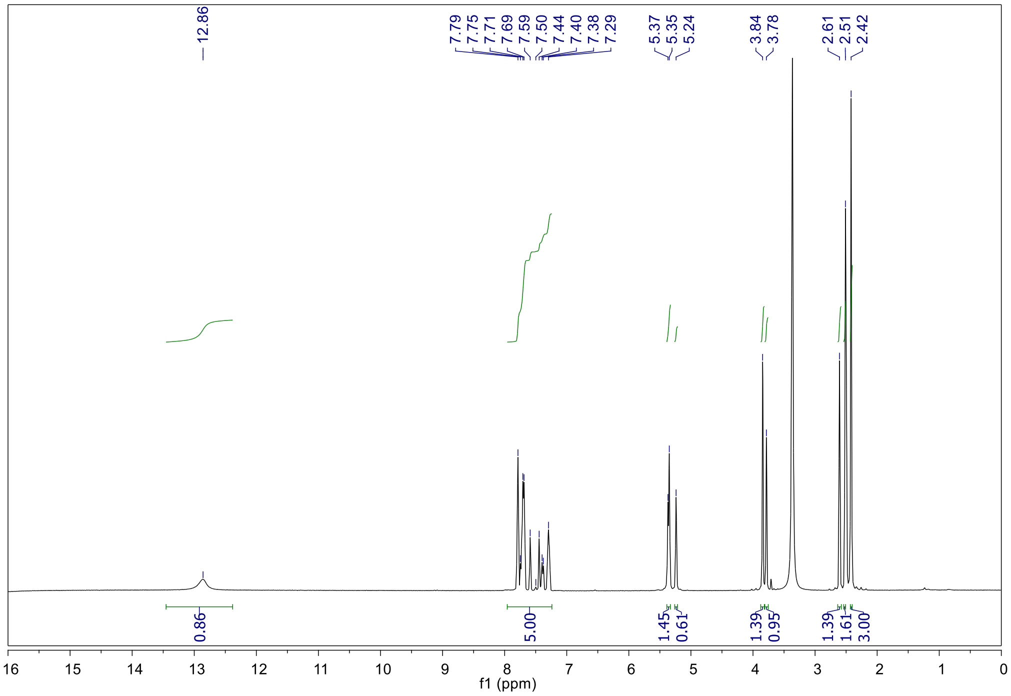

Figure S107. ^{1}H NMR of Compound **29**.

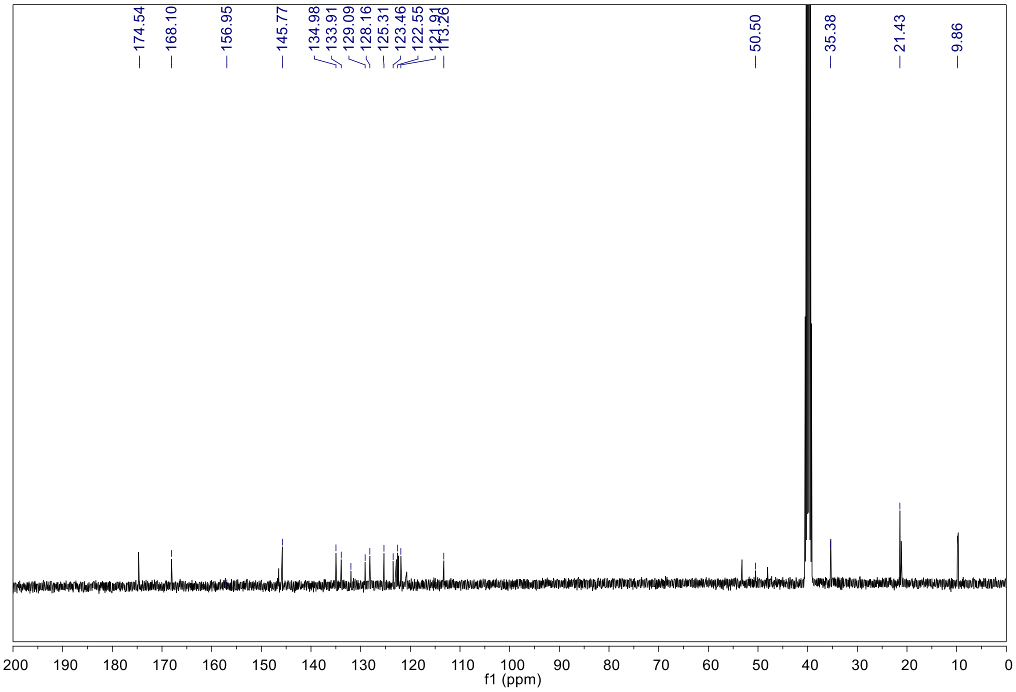

Figure S108. ^{13}C NMR of Compound **29**.

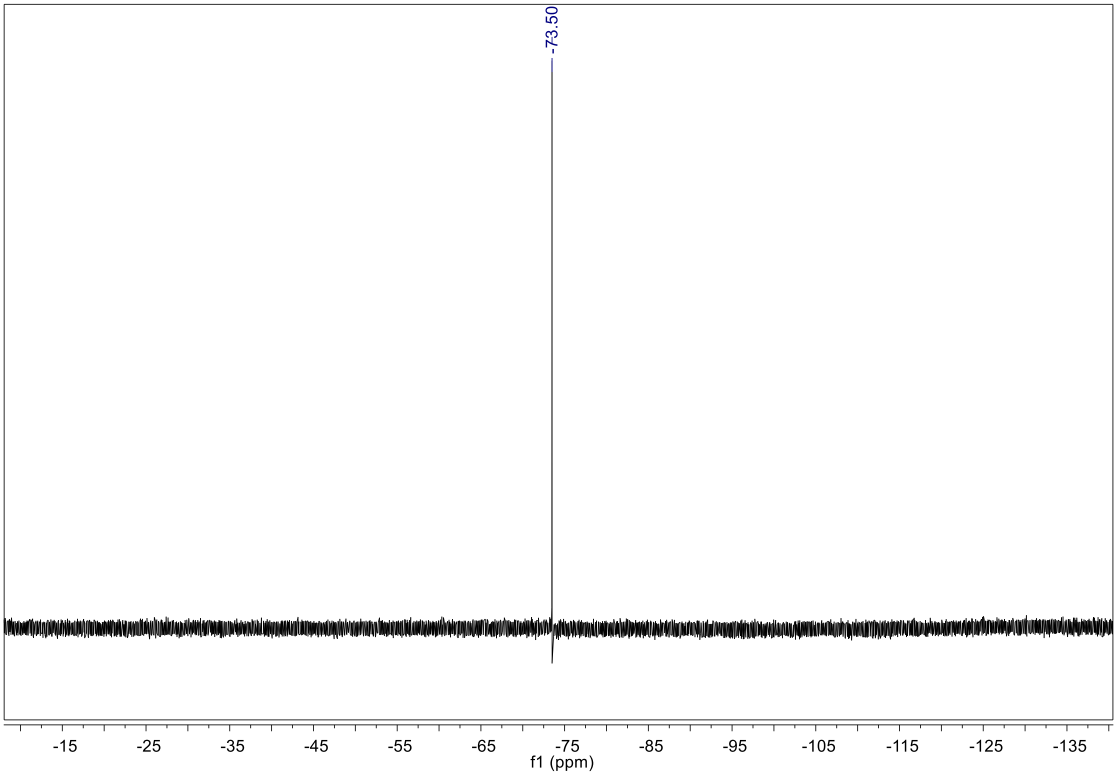

Figure S109. ^{19}F NMR of Compound **29**.

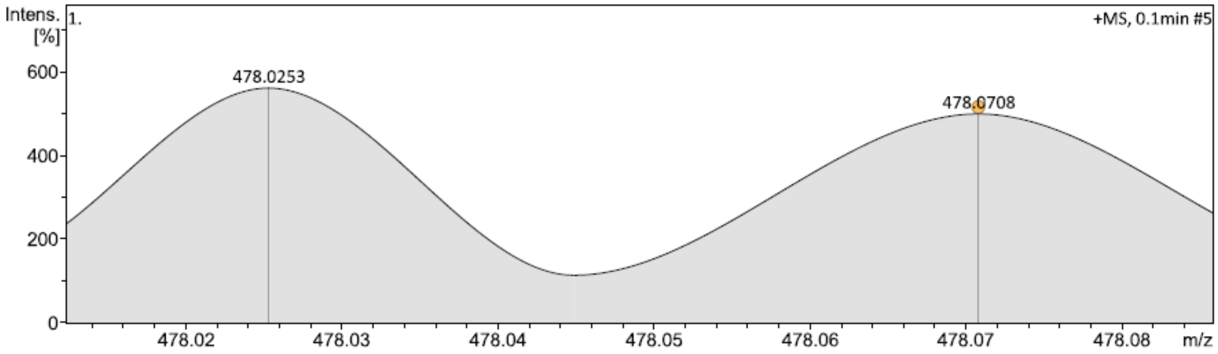

Figure S109. HRMS (ESI) of Compound **29**.
